# Phase I Safety and Immunogenicity Evaluation of MVA-CMDR, a Multigenic, Recombinant Modified Vaccinia Ankara-HIV-1 Vaccine Candidate

**DOI:** 10.1371/journal.pone.0013983

**Published:** 2010-11-15

**Authors:** Jeffrey R. Currier, Viseth Ngauy, Mark S. de Souza, Silvia Ratto-Kim, Josephine H. Cox, Victoria R. Polonis, Patricia Earl, Bernard Moss, Sheila Peel, Bonnie Slike, Somchai Sriplienchan, Prasert Thongcharoen, Robert M. Paris, Merlin L. Robb, Jerome Kim, Nelson L. Michael, Mary A. Marovich

**Affiliations:** 1 United States Military HIV Research Program (MHRP), Rockville, Maryland, United States of America; 2 Armed Forces Research Institute for Medical Sciences (AFRIMS), Bangkok, Thailand; 3 International AIDS Vaccine Initiative (IAVI), New York, New York, United States of America; 4 Laboratory of Viral Diseases, National Institute of Allergy and Infectious Diseases (NIAID), National Institutes of Health, Bethesda, Maryland, United States of America; 5 Mahidol University, Bangkok, Thailand; University of Sao Paulo, Brazil

## Abstract

**Background:**

We conducted a Phase I randomized, dose-escalation, route-comparison trial of MVA-CMDR, a candidate HIV-1 vaccine based on a recombinant modified vaccinia Ankara viral vector expressing HIV-1 genes *env*/*gag*/*pol*. The HIV sequences were derived from circulating recombinant form CRF01_AE, which predominates in Thailand. The objective was to evaluate safety and immunogenicity of MVA-CMDR in human volunteers in the US and Thailand.

**Methodology/Principal Findings:**

MVA-CMDR or placebo was administered intra-muscularly (IM; 10^7^ or 10^8^ pfu) or intradermally (ID; 10^6^ or 10^7^ pfu) at months 0, 1 and 3, to 48 healthy volunteers at low risk for HIV-1 infection. Twelve volunteers in each dosage group were randomized to receive MVA-CMDR or placebo (10∶2). Volunteers were actively monitored for local and systemic reactogenicity and adverse events post vaccination. Cellular immunogenicity was assessed by a validated IFNγ Elispot assay, an intracellular cytokine staining assay, lymphocyte proliferation and a ^51^Cr-release assay. Humoral immunogenicity was assessed by ADCC for gp120 and binding antibody ELISAs for gp120 and p24. MVA-CMDR was safe and well tolerated with no vaccine related serious adverse events. Cell-mediated immune responses were: (i) moderate in magnitude (median IFNγ Elispot of 78 SFC/10^6^ PBMC at 10^8^ pfu IM), but high in response rate (70% ^51^Cr-release positive; 90% Elispot positive; 100% ICS positive, at 10^8^ pfu IM); (ii) predominantly HIV Env-specific CD4^+^ T cells, with a high proliferative capacity and durable for at least 6 months (100% LPA response rate by the IM route); (iv) dose- and route-dependent with 10^8^ pfu IM being the most immunogenic treatment. Binding antibodies against gp120 and p24 were detectable in all vaccination groups with ADCC capacity detectable at the highest dose (40% positive at 10^8^ pfu IM).

**Conclusions/Significance:**

MVA-CMDR delivered both intramuscularly and intradermally was safe, well-tolerated and elicited durable cell-mediated and humoral immune responses.

**Trial Registration:**

ClinicalTrials.gov NCT00376090

## Introduction

Globally, an estimated 33.4 million people currently live with HIV/AIDS and in 2008 alone, an estimated 2.7 million new infections occurred [1]. Controlling the global HIV pandemic will require a successful AIDS vaccine [2], [3], [4]. HIV vaccine development was invigorated recently by the modest level of protective efficacy observed in the low incident Thai heterosexual population in the ALVAC-HIV/AIDSVAX B/E Phase III trial (RV144) [5]. Though the correlates of protection for RV144 remain under active investigation, attempts to improve upon the current levels of protection afforded by the prime-boost strategy used in RV144 are underway. In the absence of an immune correlate, HIV vaccine development is currently directed towards the quantitative and qualitative improvement of vaccine-induced responses through the use of novel vectors alone, or in prime-boost configurations [2], [4]. The inclusion of ALVAC, a poxvirus-based vector, as a component of the Thai Phase III trial suggests that improved poxvirus vectors may be effective components of a realistic strategy for vaccination against HIV infection.

One such promising attenuated poxvirus vector is modified vaccinia Ankara (MVA). The potential of MVA as a safe and effective vector for vaccine development was demonstrated by its use during the smallpox eradication campaign in Germany where over 120, 000 people were immunized without adverse effects [6], [7]. We report here a phase I safety and immunogenicity study with a recombinant MVA-HIV vaccine expressing *env*/*gag*/*pol* inserts derived from a CRF01_AE HIV-1 isolate from Chiang Mai, Thailand, referred to here as MVA-CMDR (Chiang Mai Double Recombinant). The construction details and pre-clinical testing of this vaccine were published earlier [8]. MVA-CMDR has been used as a heterologous boost at the Karolinska Institute, Stockholm, Sweden and the Muhumbili University, Dar es Salaam, Tanzania in previous multigenic and multiclade DNA-prime/MVA-boost trials [9]. This phase I trial was conducted in the US and Thailand focusing on dose escalation and route comparisons of MVA-CMDR alone for the induction of cellular and humoral immunogenicity in a vaccinia naïve population.

## Materials and Methods

### Study vaccine candidate

The MVA-CMDR was developed through collaboration between the Laboratory of Viral Diseases (LVD)/National Institute of Allergy and Infectious Diseases (NIAID) and the Walter Reed Army Instiutite of Research (WRAIR)/US Military HIV Research Program (MHRP) [8]. This multigenic vaccine contains *env/gag/pol* inserts derived from CRF01_AE isolates from Chiang Mai (CM), Thailand (HIV-1 CM235 *env*/CM240 *gag*/*pol*) [10]. The product was produced and vialed under Good Manufacturing Practice (GMP) at the WRAIR Pilot Bioproduction Facility (Forest Glen, Silver Spring, MD). The placebo formulation was PBS with 7.5% lactose, pH7.4 and was identical to the vaccine diluent.

### Ethics statement, protocol authorization and regulatory approval

The clinical trial protocol and all related documents were approved by the following independent Institutional Review Boards (IRBs): Division of Human Subject Protection, Walter Reed Army Institute of Research; Ethical Review Committee for Research in Human Subjects, Ministry of Public Health, Thailand; and Siriraj Institutional Review Broad, Faculty of Medicine, Siriraj Hospital Mahidol University. The MVA-CMDR vaccine product was evaluated in an IRB approved phase I study (RV158) under WRAIR Protocol #1143. The study was registered with www.clinicaltrials.gov, NCT00376090, and was conducted under FDA-IND #12267 by the MHRP with the sponsorship of the Office of the Surgeon General, Department of the Army, U.S. Army Medical Materiel Development Activity (USAMMDA). The study was conducted in accordance with the International Conference on Harmonization, Good Clinical Practice guidelines (ICH-GCP). All volunteers provided written informed consent following discussion and counseling by the clinical study team prior to enrollment and before any trial related procedures were performed. The protocol for this trial and supporting CONSORT checklist are available as supporting information; see Checklist S1 and Protocol S1.

### Study design, vaccination regimen and recruitment

RV158 was conducted in Rockville, MD (MHRP Vaccine Research Clinic) and in Bangkok, Thailand (Siriraj Hospital and AFRIMS Clinical Trial Center). A total of 48 healthy, HIV-negative, volunteers (18–49 years of age) were enrolled and randomized to receive vaccine or placebo (10∶2 per group). As shown in **Table 1**, subjects were vaccinated intradermally (ID) with either 10^6^ or 10^7^ plaque-forming units (pfu) of MVA-CMDR (0.1 ml ID into the volar aspect of the forearm), or intramuscularly (IM) with either 10^7^ or 10^8^ pfu (1 ml IM into the deltoid muscle). The vaccines were administered at 0, 1 and 3 months for a total of 3 doses. The low dose Part A was conducted in the US to assess safety and then Part B enrollment was split equally between the US and Thailand sites.

**Table 1:** RV158 Study Design.

Part	Group	Randomization (Vaccine∶Placebo)	Dose/Route	Schedule (months)**
PART A	I*	10∶2	10^7^ pfu IM	0, 1, 3
(Low Dose)	II	10∶2	10^6^ pfu ID	0, 1, 3
PART B	III	10∶2	10^8^ pfu IM	0, 1, 3
(High Dose)	IV*	10∶2	10^7^ pfu ID	0, 1, 3

*Route comparison groups receiving the same dose.

**All participants followed for 6 months after final vaccination.

### Safety assessment and clinical laboratory evaluations

Eligibility was assessed over two visits (V1, V2). Eligible volunteers were healthy, aged 18–49 years, HIV uninfected, vaccinia naïve by serology and absence of physical evidence of variolation or history of smallpox vaccination, and exhibited a normal baseline electrocardiogram (ECG). Eligible, consenting volunteers were enrolled and randomized to the different Groups: 1, 2, 3 or 4. They were block randomized within each group to receive vaccine versus placebo (5∶1 ratio). Volunteers were observed in the clinic for 45 minutes after vaccination for early post-injection reactions. During the observation period the volunteer was taught how to complete a 6-day diary card for any symptoms that might develop later. Within 24–48 hours post-vaccination, volunteers received a phone call inquiring of adverse reactions. Volunteers returned to the clinic two weeks post-vaccination for a safety visit. This safety visit included solicitation and review of adverse events, physical examination, ECG, and blood for safety labs and immunogenicity studies. Two other immunogenicity and associated follow-up visits for laboratory results completed the trial schedule of 11 visits over a 12-month period (9 month follow up post-vaccination).

### HIV testing strategy

HIV testing was conducted day -90, 42, 98, and 252 using Genetic Systems rLAV HIV-1 Enzyme Immunoasssay (EIA)(Bio-Rad Laboratories, Hercules, CA), Genetic Systems HIV-1 Western Blot (Bio-Rad Laboratories) and the Amplicor HIV-1 RNA Test version 1.5 (Roche Diagnostics, Indianapolis, IN). All assays were performed in parallel. Results of the nucleic acid tests were used to determine HIV infection status regardless of serology. Test results were reported to the clinic staff as either “HIV positive” or “HIV negative” since vaccine induced sero-reactivity (VISR) to *in vivo* expressed vaccine antigens may lead to false-positive HIV EIA and/or WB results and thus unblind staff to a volunteer's allocation (placebo or vaccine). Pre- and post-test HIV counseling was performed at each visit.

### Vaccinia exposure testing

Pre-vaccination sera were sent to V-Bio (St. Louis, MO) for Vaccinia ELISA testing. The enzyme-linked immunosorbent assay (ELISA) for vaccinia measures the level of vaccinia specific antibody (IgG) in serum samples. The immunoenzymatic method allows quantification of the virus specific antibody based on a capture technique and subsequent color development measurement by a spectrophotometer. The vaccinia IgG ELISA procedure has been described previously and was modified as described [11]. Briefly, plates were coated with vaccinia antigen or negative (mock-infected) cell culture lysate. Serial 2-fold dilutions of sera were placed on both antigen-coated and mock-antigen coated wells and incubated for two-hours at 37°C. After washing horseradish peroxidase-conjugated anti-human IgG was added to the plate followed by a two hour incubation at 37°C. After the incubation period, the plates were washed and ABTS substrate (Kirkegaard and Perry, Gaithersburg, MD) was added. Following a 30-minute incubation at room temperature, stopping solution (1%SDS) was added to the plates and the plates were read at 405/492nm dual wavelength. Linear regression plots were prepared and endpoint titers were determined based on an optical density (OD) cut-off of 0.30 using UnitWin software.

### Cellular Immunogenicity Assessment

#### Blood Collection

Peripheral blood mononuclear cells (PBMC) for cellular immunogenicity assays were isolated from whole blood collected in acid-citrate dextrose anti-coagulant using standard procedures [12]. PBMC were either used fresh or cryopreserved in RPMI media containing 20% fetal calf serum and 10% dimethyl sulphoxide (DMSO) in the vapor phase of liquid nitrogen or electric freezers (Revco) at ≤−130°C. All PBMC processing was undertaken within 6 hours of blood collection, and post-thaw PBMC viability was greater than 80% for all samples tested.

#### Chromium (^51^Cr)-release cytotoxic T lymphocyte (CTL) assay

A standard chromium-release assay for CTL function was performed. Effector cells were generated following a 2-week *in vitro* stimulation co-culture of 16×10^6^ freshly isolated PBMC and 4×10^6^ PBMC infected with 5 pfu/cell of MVA-CMDR. The culture was supplemented with 3.3 µg/ml of rIL-7 at the time of initiation and was further supplemented with 20 U/ml of rIL-2 after 1 week. Target cells were autologous EBV-transformed B cells (TBC) infected overnight with single recombinant MVA constructs expressing either CM240 Gag/Pol or CM235 Env (matching the MVA-CMDR insert sequences) and loaded with ^51^Cr sodium chromate. Lytic activity of the effector cells was determined at E∶T ratios of 50∶1 and 25∶1, with CD4 or CD8 dependence verified using immunomagnetic bead depletion. Specificity of the response for the insert sequences was further verified using cold target quenching with MVAp581 infected TBC (30∶1 cold∶hot target cells) of the vector-specific responses. A positive response was defined as ≥10% specific lysis for at least one E∶T ratio and at least a 50% reduction of lytic actvity using immunomagnetic bead depletion. Vector-specific responses were verified by the requirement of at least 50% quenching of lysis by cold target addition. All reported data are based upon CD8-dependence of the responses. CD4-dependent responses were rare, and no difference between vaccine and placebo groups was observed (data not shown).

#### Interferon-gamma (IFNγ) Elispot assay

A validated IFNγ Elispot assay was performed using cryopreserved PBMC and pools of synthetic peptides (15-mers overlapping by 11 amino aicds, of >80% purity), or direct addition of MVA passage 581 (whole virus at 5 pfu/cell) to determine the anti-insert and anti-vector responses, respectively. Staphylococcal enterotoxin B (SEB) was used as a positive control. Peptides for stimulation were synthesized by New England Peptides (Gardner, MA) and Sigma-Aldrich (St. Louis, MO) and pooled in a quality-controlled laboratory. Briefly, 2×10^5^ PBMC were added to each well of a Millipore MAIP Elispot plate that was pre-coated with anti-IFNγ mAb 1-D1K (Mabtech-AB, Sweden) and incubated with the various stimuli for 18–20 hours. The presence of spot forming cells was determined by detection with anti-IFNγ mAb 7-B6-1 (Mabtech-AB, Sweden) and color development using the VectorStain kit 2-hour incubation. All tests were performed in triplicate and the negative control performed in replicates of six wells. Determination of a positive Elispot value derived from validation of the assay and required the following criteria to be met: 1) the spot forming cell (SFC) count of the “media only” wells (3–4 per plate) was <10 SFC/10^6^ PBMCs; 2) the mean background SFC count (PBMC only) had to be <100 SFC/10^6^ PBMCs; 3) the SFC count for the peptide pool wells was>27 SFC/10^6^ PBMCs; and 4) the mean SFC count was >4 times the mean background SFC count. Applying these criteria to 49 pre-immunization samples tested with each of the three peptide pools representing Gag, Pol and Env, yielded a false positive rate of <1% (1/147 positive responses).

#### Tritiated (^3^H)-thymidine antigen-specific lymphocyte proliferation assay (LPA)

The proliferative responses of volunteer PBMC were measured by incubating 1×10^5^ cells per well in 96-well U-bottom polystyrene plates with serial dilutions (200 and 100 ng/ml) of Whole aldrithiol-2 Inactivated Virus isolates CM235 (CM235_WIV_) and MN (MN_WIV_) (courtesy of Jeff Lifson, NCI Frederick, MD), serial dilutions (5 and 1 µg/ml) of TH023 gp140 (a modified soluble form of gp160, courtesy of Sanofi-Pasteur) and serial dilutions (5 and 1 µg/ml) of LAI p24 (ABL Inc., Kensington, MD). To measure the lymphoproliferative response to the MVA vector psoralen-inactivated vaccinia virus Lister strain (Advanced Biotechnologies, Columbia, MD) was used (1 µg/ml final). Tetanus Toxoid (TT) (Staten Serum Institute, Copenhagen, Denmark) was used at 5 µg/ml as a recall antigen control, and in a separate plate PBMC were cultured with control mitogens: 2 µg/ml of PHA, 1.25 µg/ml of pokeweed mitogen and 20 µg/ml of concanavalin A (Sigma-Aldrich, St Louis, MO, USA). After 3 days of incubation with mitogens and 6 days with the antigens, cells were pulsed with 1 µCi/well of [^3^H]-thymidine for 6 hr then harvested, counted and assessed for [^3^H]-thymidine incorporation. The data are expressed as a lymphocyte stimulation index (LSI = (PBMC cpm with antigen)/(PBMC cpm with medium)), to define antigen specificity. Individuals were designated as responders to a given antigen if the LSI in response to that antigen ≥5.

#### Multi-functional flow cytometry (MFC) assay

Cryopreserved PBMC were thawed, washed and resuspended in RPMI1640 with v/v 10% NHS and then co-incubated for 16–20 hrs in the presence of peptide pools (1 µg/peptide/ml) representing Gag, Pol or Env (MVA-CMDR insert vaccine matched), the positive control SEB (10 ng/ml) or 5 pfu/cell of MVA (passage 581, MVA vector-backbone). Anti-CD107a-FITC and anti-CD28/CD49 MAbs (BD Pharmingen) were included in the assay mix at set-up, while the protein transport inhibitors Monensin (GolgiStop, BD Pharmingen) and Brefeldin A (Sigma-Aldrich) were added 2 hours after set-up for the peptide and SEB stimulations, and 6 hours after assay set-up for the MVA stimulation. The following day, plates were washed, stained with Aqua Live/Dead (Invitrogen Inc.), washed and resuspended in FACSwash buffer (0.5% BSA, 0.1% azide), followed by surface staining with anti-CD14/CD19-Alexa700 (BD Pharmingen), and then simultaneous surface/intracellular staining with anti-CD4-ECD (Coulter), anti-IFNγ-PB (eBioscience), anti-TNFα-PE-Cy7 and anti-MIP1β-PE (BD Pharmingen), anti-CD3-APC-H7, anti-CD8-PerCPCy5.5, and anti-IL2-APC (BD Biosciences). Cells were acquired on a custom built BD LSR II cytometer (Becton Dickenson, San Jose, CA). At least 250,000 total events were acquired in the lymphocyte gate and the data analyzed using the following software packages: FlowJo Version 8 (Treestar Inc., Ashland, OR, USA), and PESTLE and SPICE (courtesy Mario Roederer, Vaccine Research Center, NIH, USA) software. The peptides used for this assay were synthesized and pooled by JPT Inc. Peptide pools consisted of 16-mers overlapping by 11 amino acids matched to the CRF01_AE sequences encoded in the vaccine, and spanned all gene-products. Each gene-product was represented in a single peptide pool. A positive response was defined as ≥0.025% gated positive cells and the test antigen response exceeding the un-stimulated control by >3 times.

#### Whole Blood ICS

Both sites used a standard protocol and results from the two sites were comparable. Whole blood was lysed and stimulated according to a protocol developed on the basis of previous reported data [13]. CM235_WIV_ and a Gag peptide pool that matched the vaccine were used as stimuli together with SEB and the CD8-restricted Cytomegalovirus/Epstein-Barr virus/Influenza virus (CEF) peptide pool [14] as positive controls. For the high dose groups a pool of HIV-1 92THO23 (a CRF01_AE isolate similar to CM 235) Env peptides (15-mers overlapping by 11 amino acids) was used as a stimulus. Un-stimulated cells were included in the panel and represent the background. Cells were then stained with the following panel: CD3-APC, CD4-FITC, CD8-PerCpCy5.5, IL2-PE and IFNγ-PE (combined). At least 60,000 CD3^+^ events were collected on a FACScaliber and data were analyzed using FlowJo software (Version 8). A positive response was defined as ≥0.05% gated positive cells and the test antigen response exceeding the unstimulated control by ≥3 times.

### Humoral Immunogenicity Assessment

#### ELISA measurement of serum IgG binding to Gag p24 or Env gp120

Proteins dissolved in phosphate-buffered saline (PBS; pH 7.4) containing 0.01% thimerosal were coated onto 96-well Immulon 2 microtiter plates (Fisher Scientific, Pittsburgh, PA, USA) and incubated overnight at 4°C. The protein concentrations were as follows: Baculovirus-derived p24 at 0.5 µg/ml (ImmunoDiagnostics, Inc., Woburn, MA, USA), *E. coli*-derived p24 at 0.5 µg/ml (generously provided by Venigala Rao, Catholic University of America, Washington, DC, USA) or Baculovirus-derived gp120 from clade CRF01_AE strain CM243, at 0.25 µg/ml (Protein Sciences Corp. Meridian, CT, USA). Both sources of p24 were from the clade B IIIB strain. After coating, plates were washed three times with wash buffer (PBS with 0.1% Tween 20 [pH 7.4]) and incubated for 1 hour at 37°C with two-fold dilutions of serum diluted in serum wash buffer with 5% skim milk (pH 7.4). The plates were then washed and horseradish peroxidase-conjugated goat anti-human IgG diluted 1∶16,000 in serum diluent (Goat anti-human IgG: Kirkgaard & Perry, Gaithersburg, MD, USA) was added for 1 hour at 37°C. The plates were again washed and substrate (p24-ABTS, gp120-TMB; Kirkgaard & Perry) was added for 30 min. Plates were read by spectrophotometry at 405nm, 490nm reference filter (for ABTS substrate) or at 410nm, 570nm reference filter (for TMB substrate) to determine OD values. For all assays, the endpoint titers were determined as the reciprocal of the highest serum dilution yielding OD values that were greater than the negative control cut-off value. The cut-off value was calculated as two times the average OD (+2 SD) for HIV-negative normal human sera. Each sample was run by two independent operators and the concordant values (values that were within 4-fold of each other) were averaged to yield the reported reciprocal titer. Quantitative results are presented as geometric mean titers (GMT) for each group.

#### Antibody dependent cellular cytotoxicity (ADCC) assay

The assay has been described in detail elsewhere [15], [16]. Briefly, PBMC from Thai HIV seronegative donors were used as effector cells. ^51^Cr labeled CEM.Nk^r^ cells coated with either CRF01_AE gp120 (CM243) or subtype B gp120 (MN) were used as target cells at an E∶T ratio of 100∶1 in the presence of plasma at 10-fold dilutions ranging from 1∶100 to 1∶1000000. Each dilution was performed in triplicate. Effectors and targets were incubated for 6 hours at 37°C. HIV-specific ADCC activity was calculated for each serum dilution as % Specific lysis (SL): [Mean cpm (HIV antigen)- Mean cpm (spon)/Mean cpm (max)-Mean cpm (spon)] where the max and spon were the mean cpm released by the target cells in the presence of 10% SDS or media, respectively. Inter-assay variability was reduced by converting the SL to relative lysis (RL): [%SL (max) post-vaccination - %SL (max) pre-vaccination/%SL (max) positive control-%SL (max) negative control)] where % SL (max) represents the maximum % SL obtained from a participant's post-vaccination serum over the range of plasma dilutions, % SL pre-vaccination (max) represents %SL at the same dilution as the post-vaccination sample, %SL (max) positive control represents maximum %SL obtained from the positive plasma control at one dilution and %SL (max) negative control represents %SL at the same dilution as the positive plasma control. Additionally, we compared groups using a dichotomous outcome (positive or negative) based on the 90^th^ percentile of the % RL for the placebo group for each corresponding antigen as a cut-off for a positive response.

### Data Analysis

All laboratory staff remained blinded as to placebo and vaccine status of the samples during assay performance and analysis. Data were analyzed from all participants, including those not completing the vaccination series and assessed statistically using non-parametric statistical tests where appropriate. For the qualitative assessment of response rates, proportions were compared in 2 by 2 contingency tables using Fisher's exact test. The χ^2^ trend test was used to investigate trends in increasing response and event rates in the dosage groups. Quantitative comparisons between two groups were performed using the Mann-Whitney U test (for unmatched pairs) or Wilcoxon signed rank test (for matched pairs). For comparisons of multiple groups the Kruskal-Wallis test was performed with correction using Dunn's multiple comparison test. A *p*-value of <0.05 was considered statistically significant.

## Results

### Enrollment, participant flow and demographics

**Figure 1:**
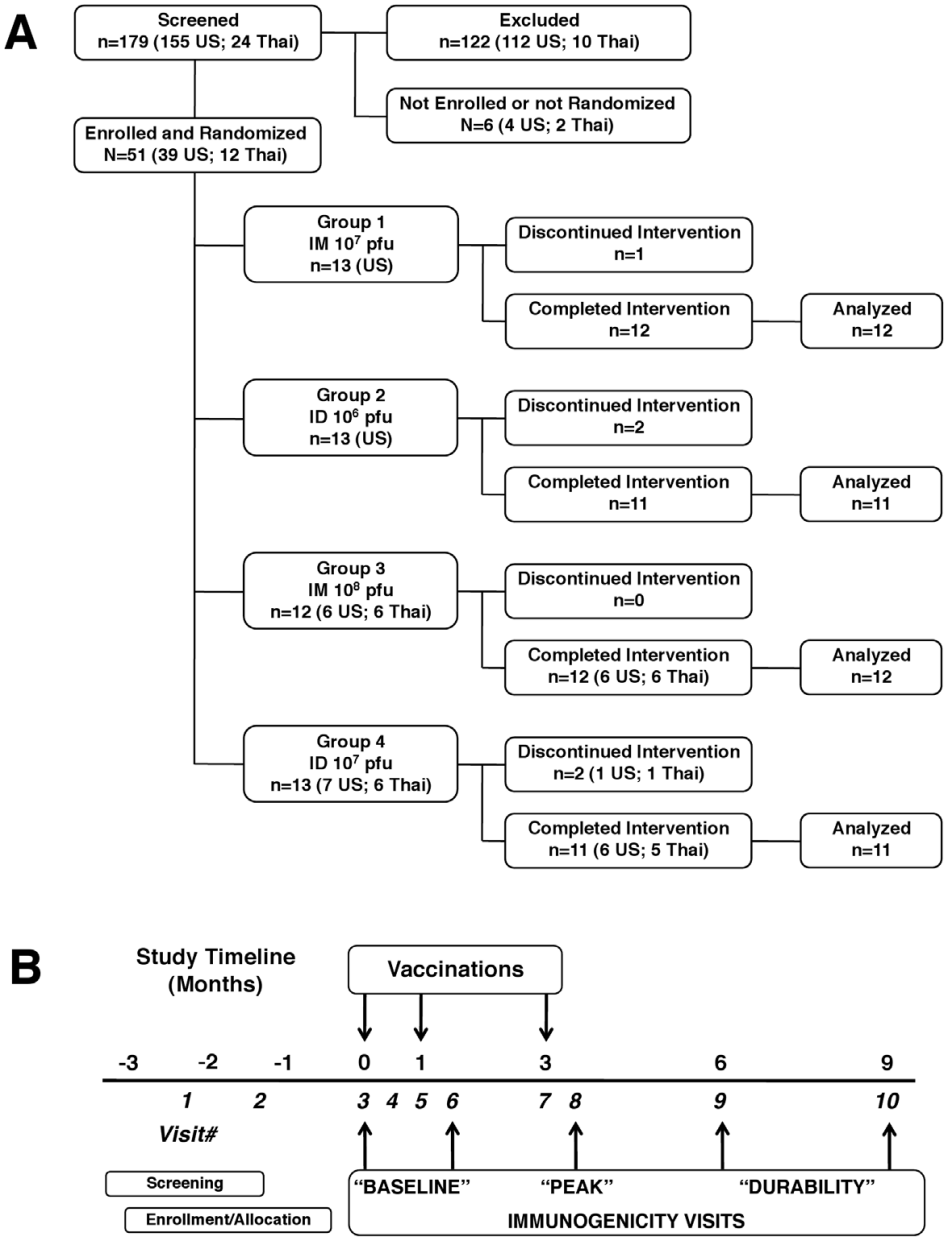
Consort clinical trial participant flow diagram (panel A). Chronological schematic diagram showing all pre-enrollment, vaccination and blood collection visits for RV158 (panel B). Immunogenicity testing visits 3, 6, 8, 9 and 10 correspond to days 0, 42, 98, 168 and 252 post-vaccination initiation respectively.

As shown in the trial participant Consort diagram (**Figure 1A**), 179 subjects (155 US, 24 Thai) were screened for this study, of whom, 51 (39 US, 12 Thai) were eligible enrollment. Due to the open enrollment nature of the study we were able to enroll three additional participants to replace three subjects who dropped out of the study prior to completion of the enrollment phase. The last study visit was completed on 25 November 2008. Demographics were similar between vaccine and placebo recipients. Mean volunteer age was between 30–32 years with a predominately male (66%) and African-American (61%) representation at the US site. The Thai sites only enrolled Thai citizens. Most potential volunteers aged >35 or over were excluded because of pre-existing vaccinia immunity. Five individuals did not complete the vaccination series in the vaccine group; four were in the ID group (2 low dose, 2 high dose) and one was in the low dose IM group. Reasons for discontinuation of vaccination after a first dose included the development of pruritis without rash, social stigma from community, non-cardiac chest pain, after missed visits due to elective kidney donation and flu-like symptoms. All volunteers missing vaccination were followed up for safety and did well for the remainder of the study. All volunteers in the placebo groups completed the vaccination series (8/8). Figure 1B shows the study timeline, highlighting the screening visits, the vaccination schedule and follow-up visits for safety and immunomonitoring.

### Safety and tolerability

**Figure 2:**
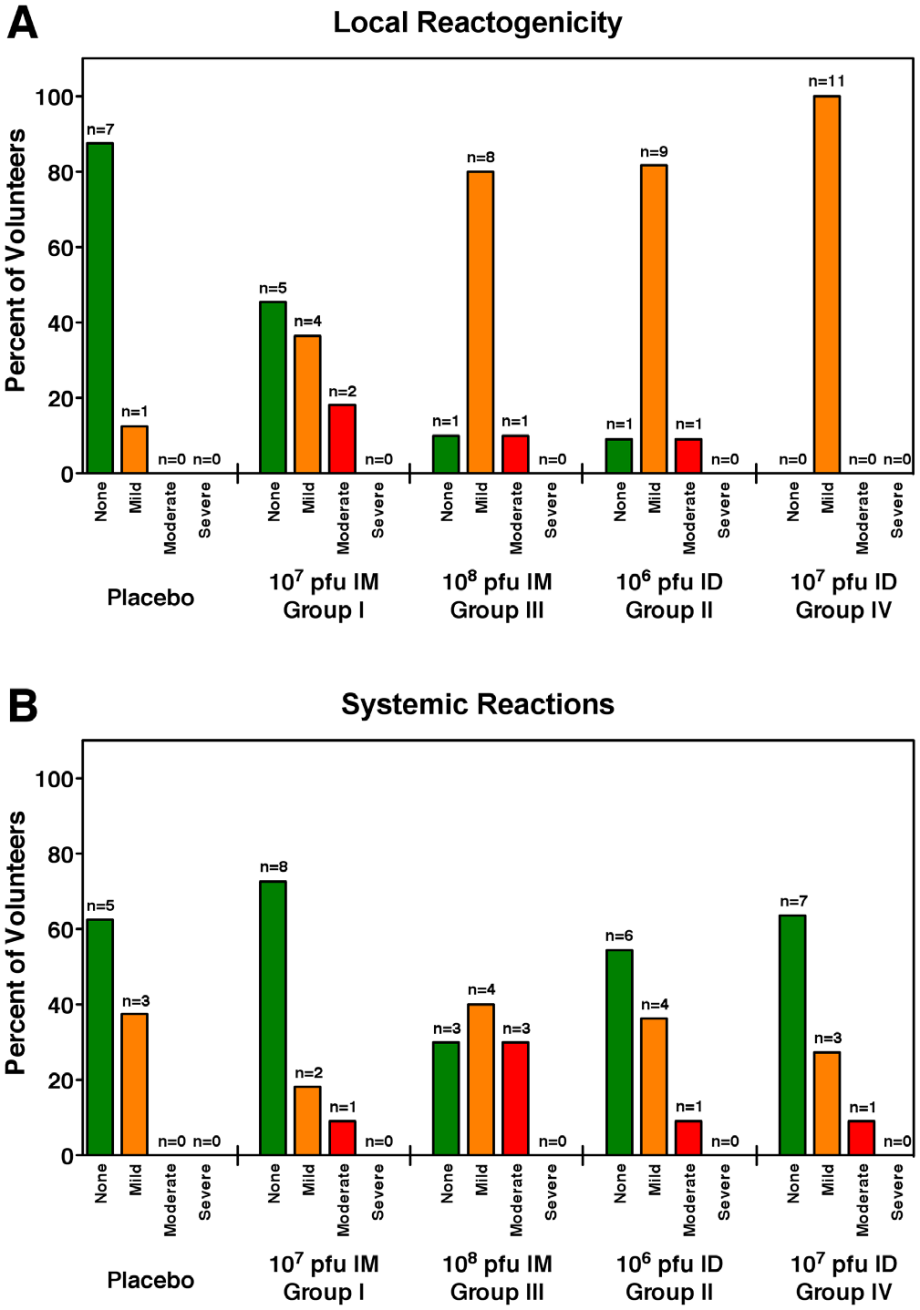
Systemic and local vaccine related reactogenicity for each dose and route of vaccination. The number and percent of subjects experiencing one or more local (panel A) or systemic (panel B) reactions is shown for each group after stratification by severity. The most severe reaction experienced by a volunteer determined the stratification into none, moderate, mild or severe categories. No serious or life threatening adverse events were reported.

The vaccine had an excellent safety profile. No related serious adverse events occurred during the study. There were no deaths, pregnancies or HIV infections during the study. The highest grade AE experienced was a Grade 2. The vaccine arm of the study had more local and systemic adverse events than the placebo arm. Vaccine recipients in the high-dose ID (Group 4) reported more local reactogenicity such as injection site tenderness, pain, erythema, induration, swelling, and itching than the low-dose IM group (Group 1). Most systemic reactions were of mild or moderate severity, such as fatigue, headache, nausea, myalgia, arthralgia, diarrhea, pruritis, loss of appetite and chills. There was no statistical difference in the rate of systemic reactions among the treatment groups. There was one individual who reported chest pain associated with fatigue, dizziness, and headache. An evaluation, which included cardiac enzymes and ECG, determine the chest pain was non-cardiac in nature. One volunteer in Group 4 had a temperature of 38.7°C (Grade 2) that resolved in 24 hours. There was no pattern of laboratory abnormalities and no significant differences in laboratory evaluations between vaccine and placebo recipients. **Figure 2** summarizes the reported adverse events for systemic and local reactogenicity.

### Cellular Immunogenicity

#### Chromium (^51^Cr) release CTL assay

Results of the chromium release CTL assay are shown in **Table 2**. Responses were assessed pre-vaccination and at all 4 post-vaccination immunogenicity visits. Cumulative CD8-dependent CTL responses against any HIV gene product were detected in 63% (24/38) of all vaccinees and were predominantly Env-directed (22 Env versus 8 Gag/Pol responders). Overall, the response rate to Env was significantly greater than Gag/Pol (*p* = 0.001; Fisher's exact test) with only 2 vaccinees exhibiting Gag/Pol responses in the absence of an Env response. There was no statistically significant dose- or route-dependence of the HIV-specific responses with 56–70% of subjects responding in any group of the trial. Env responses were predominant in all groups (50–67%) with Gag/Pol responses observed most frequently in the high-dose IM group (40%). While the Gag/Pol responses did show a significant trend (*p* = 0.047; χ^2^ trend test) towards a higher positive rate with increasing dose of vaccine (**Table 2**), these data should be viewed with caution given the low overall rate of Gag/Pol CTL responses. No positive responses were detected pre-vaccination, and one placebo was positive at a single time for an Env response (1/7). Anti-vector (MVA) CD8-dependent CTL responses were detected in 73% (28/38) of all vaccine recipients, with no vector-specific responses detected pre-immunization or in the placebo group. A route-dependence of the anti-vector response was evident with both IM groups exhibiting a significantly higher response rate (100% for high-dose; 80% for low-dose) than either ID group (56% for both doses) (*p* = 0.027, Fisher's exact test). No pre-vaccination or placebo recipient CD8-dependent positive CTL responses were detected against the vector (**see supplementary Figure S1**).

**Table 2:** Cross-sectional frequency of positive CD8 CTL responses as measured in the ^51^Cr-release assay.

Dose/Route**	Antigen***	Pre-Vacc	Day 42	Day 98	Day 168	Day 252	Cumulative
(N)		0	(2 Wks post 2^nd^)	(2 Wks post 3^rd^)	(12 Wks post 3^rd^)	(24 Wks post 3^rd^)	Any Post-Vacc
	Env	0/9	3/10	2/10	2/6	4/9	6/10 (60%)
10^8^ pfu IM	Gag	0/9	1/10	1/10	1/6	1/9	4/10 (40%)
(10)	Any	0/9	3/10	2/10	2/6	5/9	7/10 (70%)
	Env	0/5	4/8	1/8	0/10	1/9	5/10 (50%)
10^7^ pfu IM	Gag	0/5	0/8	1/8	2/10	1/9	2/10 (20%)
(10)	Any	0/5	4/8	2/8	2/10	1/9	6/10 (60%)
	Env	0/6	1/9	1/9	3/6	3/8	5/9 (56%)
10^7^ pfu ID	Gag	0/6	0/9	0/9	2/6	1/8	2/9 (22%)
(9)	Any	0/6	1/9	1/9	4/6	3/8	5/9 (56%)
	Env	0/5	2/8	1/8	1/6	2/7	6/9 (67%)
10^6^ pfu ID	Gag	0/5	0/8	0/8	0/6	0/7	0/9 (0%)
(9)	Any	0/5	2/8	1/8	1/6	2/7	6/9 (67%)
	Env	0/5	0/5	0/6	1/4	0/4	1/7 (14%)
Placebo	Gag	0/5	0/5	0/6	0/4	0/4	0/6 (0%)
(7)	Any	0/5	0/5	0/6	1/4	0/4	1/7 (14%)

*The number of positive responders for each antigen at each time-point tested for each vaccination group (by route and dose) is shown. The determination of a positive response used is outlined in the Materials and Methods section. Cumulative analysis represents a positive response at any time-point post-vaccination. Responses were measured pre-vaccination, 2 weeks post-2^nd^ vaccination, 2 weeks post-3^rd^ vaccination and 12 and 24 weeks post-3^rd^ vaccination.

**Numbers in parentheses represent the total subjects tested per group.

***Responses are shown for single recombinant MVA vectors expressing the Env and Gag/Pol inserts in the double recombinant MVA-CMDR vaccine product.

#### IFNγ Elispot assay

**Table 3** summarizes the IFNγ Elispot results. Responses were assessed at one pre-vaccination time-point and all four post-vaccination immunogenicity visits. IFNγ Elispot responses against insert gene products were detected in 55% (22/40) of all vaccinees at least once post-vaccination. While there was a predominance of Env-directed Elispot responses (20 Env, 13 Gag and 2 Pol responders), the difference between Env and Gag response rates was not statistically significant (Fisher's exact test). Both Env and Gag response rates were significantly greater than the Pol response rate (*p* = 0.001 and *p* = 0.003 respectively; Fisher's exact test). Eleven of the Gag responders and all of the Pol responders were also Env responders. While a dose-dependence of the response was evident in both routes with the high-dose IM group response rate (90%) > the low-dose IM group response rate (40%), and the high-dose ID group response rate (60%) > the low-dose ID group response rate (30%), these differences were not statistically significant. The durability of the Elispot responses was reflected in the observation that 6/22 responding subjects had positive Elispot responses at all 4 post-vaccination time-points and 15/22 were still positive at 252 days post-vaccination. The magnitude of the HIV-specific IFNγ Elispot response was low (corrected SFC/10^6^ PBMC range = 27–628) (**see supplementary Figure S2**). The highest median HIV-specific Elispot response for any time-point post-vaccination was detected in the high-dose IM group at 252 days post-vaccination (median corrected SFC/10^6^ PBMC = 78). For the route comparison groups (IM and ID 10^7^ pfu) there was no difference statistically in the median Elispot responses at any time-point post-vaccination, although the IM group response exceeded the ID group at all time-points. However, greater Elispot responses were detected at days 42 and 98 post-vaccination initiation (Mann-Whitney test; *p* = 0.027 and *p* = 0.005 respectively) in the combined IM groups with respect to the combined ID groups, but this difference was not significant at days 168 and 252 post-vaccination. One pre-vaccination sample and no placebo recipients had detectable IFNγ Elispot positive responses. The anti-vector (MVA backbone) IFNγ Elispot responses showed a 90% cumulative positive response rate in the IM groups, while the ID groups showed 90% and 30% in the high-dose and low-dose vaccine recipients respectively.

**Table 3:** Cross-sectional vaccine responsiveness as measured by the Interferon-γ Elispot assay*.

Dose/Route**	Antigen***	Pre-Vacc	Day 42	Day 98	Day 168	Day 252	Cumulative
(N)		0	(2 Wks post 2^nd^)	(2 Wks post 3^rd^)	(12 Wks post 3^rd^)	(24 Wks post 3^rd^)	Any Post-Vacc
	Quantitative analysis: Median[Range] SFC/10^6^ PBMC****						
	Env	0	5	6	6	5	9 (90%)
10^8^ pfu IM	Gag	0	2	3	2	2	4 (40%)
(10)	Any	0	6	6	7	6	9 (90%)
	Quan.	3 [0–20]	57 [0–337]	71 [5–279]	66 [24–270]	78 [0–317]	
	Env	0	2	3	3	4	4 (40%)
10^7^ pfu IM	Gag	0	2	3	3	2	4 (40%)
(10)	Any	0	3	4	4	4	4 (40%)
	Quan.	4 [0–11]	19 [1–241]	20 [3–224]	19 [3–511]	19 [1–632]	
	Env	1	2	2	1	2	4 (40%)
10^7^ pfu ID	Gag	0	0	1	2	3	4 (40%)
(10)	Any	1	2	2	2	4	6 (60%)
	Quan.	11 [0–30]	17 [0–55]	12 [0–90]	53 [1–97]	44 [0–138]	
	Env	0	1	1	2	1	3 (30%)
10^6^ pfu ID	Gag	0	0	1	1	1	1 (10%)
(10)	Any	0	1	1	2	1	3 (30%)
	Quan.	2 [0–8]	2 [0–38]	7 [0–119]	4 [0–145]	7 [0–99]	
	Env	0	0	0	0	0	0 (0%)
Placebo	Gag	1	0	0	0	0	0 (0%)
(8)	Any	0	0	0	0	0	0 (0%)
	Quan.	6 [0–82]	3 [0–23]	1 [0–21]	5 [0–48]	6 [0–57]	

*The number of positive responders for each antigen at each time-point tested for each vaccination group (by route and dose) is shown. The cut-off for a positive response was 27 SFC/10^6^ PBMC. Cumulative analysis represents a positive response at any time-point post-vaccination. Responses were measured pre-vaccination, 2 weeks post-2^nd^ vaccination, 2 weeks post-3^rd^ vaccination and 12 and 24 weeks post-3^rd^ vaccination.

**Numbers in parentheses represent the total subjects tested per group.

***Responses are shown for Env and Gag peptide pools representing the vaccine inserts and also for responders to any insert-derived peptide pool. Only 2 responders were detected using the polymerase (Pol) peptide pool (1 in the 10^8^ pfu IM group and 1 in the 10^7^ pfu ID group).

****Values for the quantitative analysis represent the corrected data values (test antigen - background). The median value [with range] for the summed responses (Env+Gag+Pol) is shown as SFC/10^6^ PBMC.

#### Lymphocyte proliferation assay

**Figure 3:**
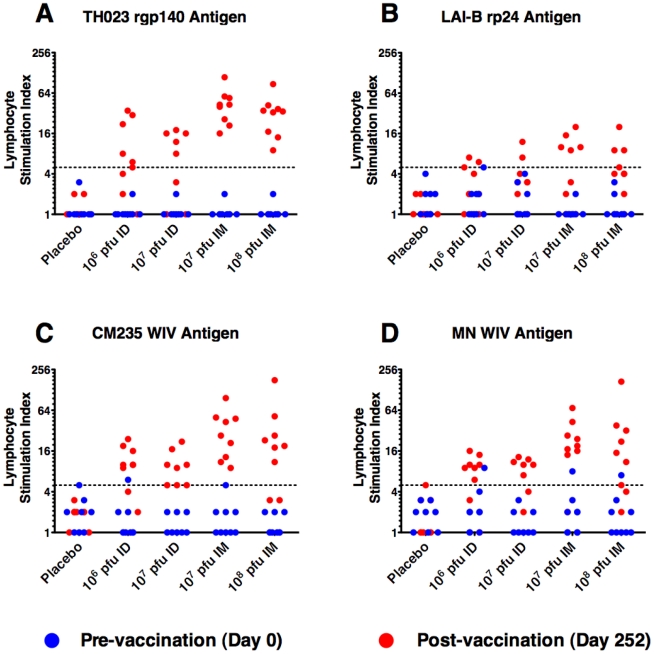
Lymphocyte proliferation responses for each dose and route. Lymphocyte proliferation responses against recombinant proteins (TH023 gp140 and LAI p24) are shown for all doses and routes of vaccination (panels A and B). HIV whole inactivated virus (WIV) antigens CM235_WIV_ (CRF01_AE vaccine matched isolate) and MN_WIV_ (subtype B heterologous isolate) are shown for all doses and routes of vaccination in panels C and D respectively. The Y-axis represents the LSI (scale = log_2_) and the dotted line designates an LSI of 5 (cut-off for positive responses). Blue and red circles represent pre- and post-vaccination (day 0 and day 252) samples respectively.

LPA responses are shown in **Figure 3**. Responses were assessed at one pre-vaccination time-point and one post-vaccination time-point (Day 252; 6 months post the 3^rd^ and final vaccination). Lymphoproliferative responses against the HIV antigens were detected in all study groups with 100% (9/9) of vaccinees responding to rgp140 in both IM groups. Responses were predominantly Env-directed with a significantly higher response rate to rpg140 compared with rp24 in the combined IM groups (*p* = 0.001; Fisher's exact test), and a non-significant trend for higher rgp140 response rates in the combined ID groups (**Figure 3** **, panels A and B**). While there were no differences in response rates or magnitude between doses within the two routes, there was a route-dependence of the response. Comparing the rgp140 responses between the two routes a statistically significant higher response rate (*p* = 0.003; Fisher's exact test) and magnitude (p = 0.001; Mann-Whitney test) was observed for the combined IM groups versus the combined ID groups. This statistical difference in magnitude of response (*p* = 0.001, Mann-Whitney test) was retained in a direct comparison of the high-dose ID and low-dose IM groups (both received 10^7^ pfu per vaccination). Aldrithiol-2 inactivated WIV isolates CM235_WIV_ and MN_WIV_ were used to test for responses against the total insert gene products and for cross-reactivity of the responding cells (**Figure 3** **, panels C and D**). Response rates against CM235_WIV_ were greatest in the IM groups (9/9 and 7/9 for low- and high-dose respectively) and lower in the ID groups (7/9 and 5/8 for low- and high-dose respectively), although this difference was not statistically significant. Again, a route-dependence in the magnitude of the proliferative response against CM235_WIV_ was observed with the low-dose IM LSI significantly higher than the high-dose ID LSI (*p* = 0.012, Mann-Whitney test). The MN_WIV_ responses mirrored the CM235_WIV_ responses in both frequency and magnitude with no statistically significant differences in the frequency or magnitude of responses. Collectively, these data demonstrate that vaccination with MVA-CMDR induces a potent insert-directed proliferative response that is route-dependent and cross-reactive between CRF01_AE and subtype B HIV-1 isolates.

#### Whole blood ICS

The whole blood ICS assay results are shown in **Table 4** including data from one pre-vaccination time-point and all 4 post-vaccination immunogenicity visits. There were no responses to the HIV antigens pre-vaccination. Most volunteers responded to the HIV antigens at one time point and in only a few cases were the responses sustained (positive at 2–3 different time points). Responses were predominantly CD4^+^ T cells and directed against the Env peptide pool (range for CD4^+^ Env responses 0.051–0.400, CD8^+^ Env 0.058–0.180, CD4^+^ Gag 0.054–0.170, CD8^+^ Gag 0.08–0.210). The data shown in **Table 4** represent any responses to the 92THO23 Env peptide pool (Env) and CM240 Gag peptide pool (Gag) at any time post-vaccination. There was a significant route-dependence of the CD4^+^ T cell response with a higher rate of responders in the combined IM groups versus the combined ID groups (*p* = 0.01, Fisher's exact test). No significant dose-dependence of the response was observed, although a trend toward a higher number of positive responders was observed in the higher dose of both routes. CD8^+^ T cell responses were much more sporadic among the four groups, with too few responses to discern a route- or dose-dependence of the response. These data show that MVA-CMDR was immunogenic eliciting predominantly CD4^+^ responses as measured by IL-2 and IFNγ gene up-regulation and protein production.

**Table 4:** Cross-sectional vaccine responsiveness by Whole Blood ICS assay*.

Dose/Route**	Antigen***	Pre-Vacc		Day 42		Day 98		Day 168		Day 252		Cumulative	
(N)		0		(2 Wks post 2^nd^)		(2 Wks post 3^rd^)		(12 Wks post 3^rd^)		(24 Wks post 3^rd^)		Any Post-Vacc	
		CD4	CD8	CD4	CD8	CD4	CD8	CD4	CD8	CD4	CD8	CD4	CD8
10^8^ pfu IM	Env	0	0	5	0	6	0	3	1	5	3	9 (90%)	3 (30%)
(10)	Gag	0	0	3	0	2	0	2	0	0	0	3 (30%)	0 (0%)
10^7^ pfu IM	Env	0	0	5	0	2	0	1	1	1	0	6 (60%)	1 (10%)
(10)	Gag	0	0	3	2	3	1	2	1	0	2	4 (40%)	4 (40%)
10^7^ pfu ID	Env	1	0	1	0	2	1	3	1	1	1	5 (62%)	3 (33%)
(9)	Gag	0	0	0	0	1	0	1	1	0	0	2 (22%)	1 (11%)
10^6^ pfu ID	Env	0	0	0	0	1	0	0	0	0	0	1 (11%)	0 (0%)
(9)	Gag	0	0	1	0	1	0	0	1	0	0	1 (11%)	1 (11%)
Placebo	Env	1	0	0	0	0	0	2	1	1	0	2 (25%)	1 (13%)
(8)	Gag	0	0	0	0	0	0	0	0	0	1	0 (0%)	1 (13%)

*The cut-off for a positive response was 3× over background and > or equal to 0.05%.

**Numbers in parentheses represent the total subjects tested per group. One volunteer in the 10^6^ pfu ID group did not have data for 2Wks post 2nd and 2 Wks post 3rd and one placebo is missing data for 24 Wks post 3rd. For this reason the subject count may vary from visit to vist.

***Responses are shown for Env and Gag peptide pools representing the vaccine inserts. See material and methods for more details.

#### Qualitative analysis of the combined cellular assays

**Figure 4:**
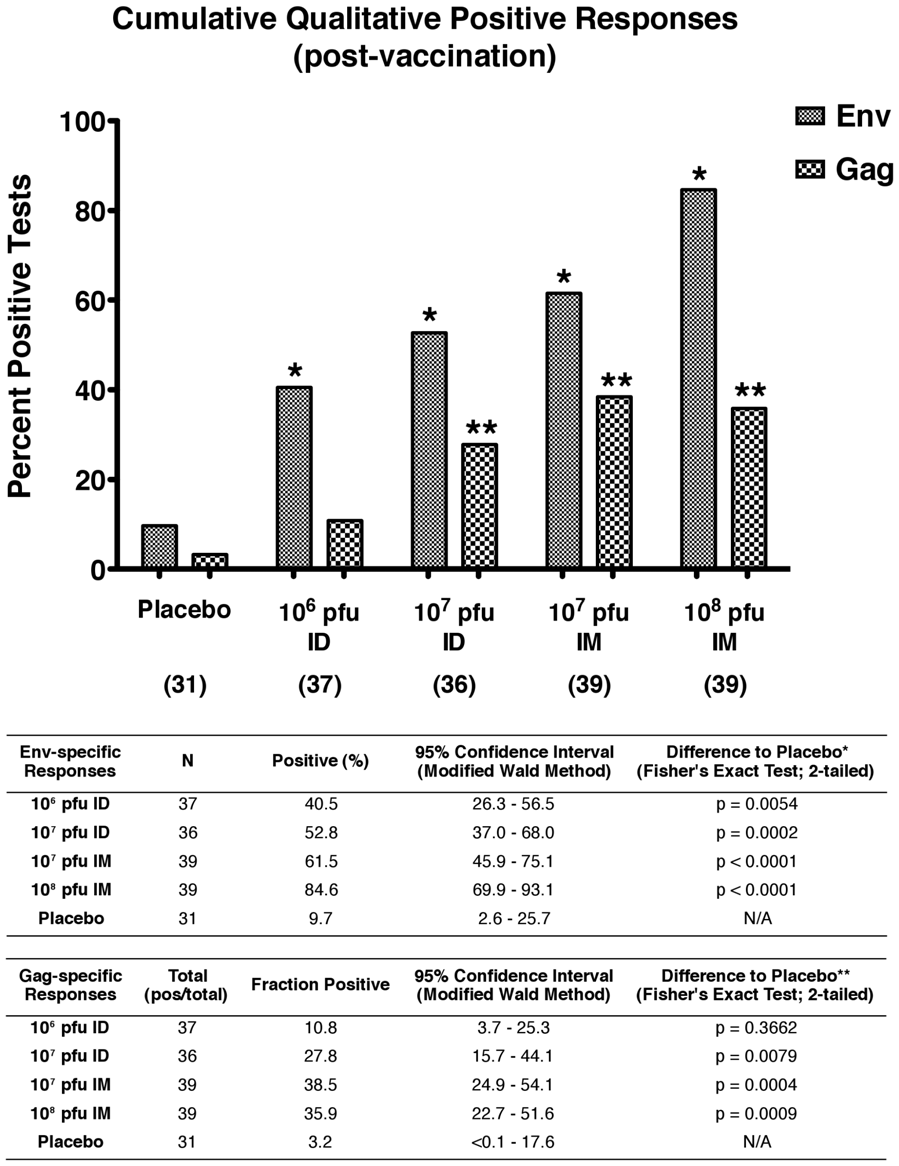
Cumulative analysis of all qualitative cellular immune response assays. The number of positive responders for HIV antigens (Env or Gag) in the ^51^Cr-release, Elispot, lymphocyte proliferation and whole blood ICS assays was tallied for all groups and compared with the placebo recipients. The figure graphically displays the summed cumulative positive responders for each of the 4 assay platforms. The total number of tests performed in each group is denoted below the dose and route. The top and bottom tables summarize the statistical analyses for Env and Gag respectively.

In order to perform a statistically robust analysis of the dose- and route-dependence of the responses generated by MVA-CMDR, the qualitative response data for the ^51^Cr-release, Elispot, lymphocyte proliferation and whole blood ICS assays were combined and analyzed for differences to placebo and trends in dose and route responsiveness. For this analysis the cumulative number of post-vaccination responders for each of the four assays were summed for each dose and route group. **Figure 4** shows the cumulative qualitative post-vaccination response data stratified by route and dose for Env and Gag (or Gag/Pol responses for the ^51^Cr-release assay). Compared with the placebo group all routes and doses showed a significantly increased response to Env antigens post-vaccination, while all but the lowest dose ID group showed a significantly increased Gag response (Fisher's exact test). For both Env and Gag a significant trend was observed (*p* = 0.001 for Env; *p* = 0.009 for Gag/Pol; χ^2^ trend test) with the qualitative response rates following the ensuing ranking: high-dose IM (10^8^ pfu)>low-dose IM (10^7^ pfu)>high-dose ID (10^7^ pfu)>low-dose ID (10^6^ pfu). Collectively, these data demonstrate that 10^8^ pfu IM is the most immunogenic dose and route of administration for induction of cellular immune responses by MVA-CMDR.

#### Multi-functional flow cytometry (MFC) assay

**Figure 5:**
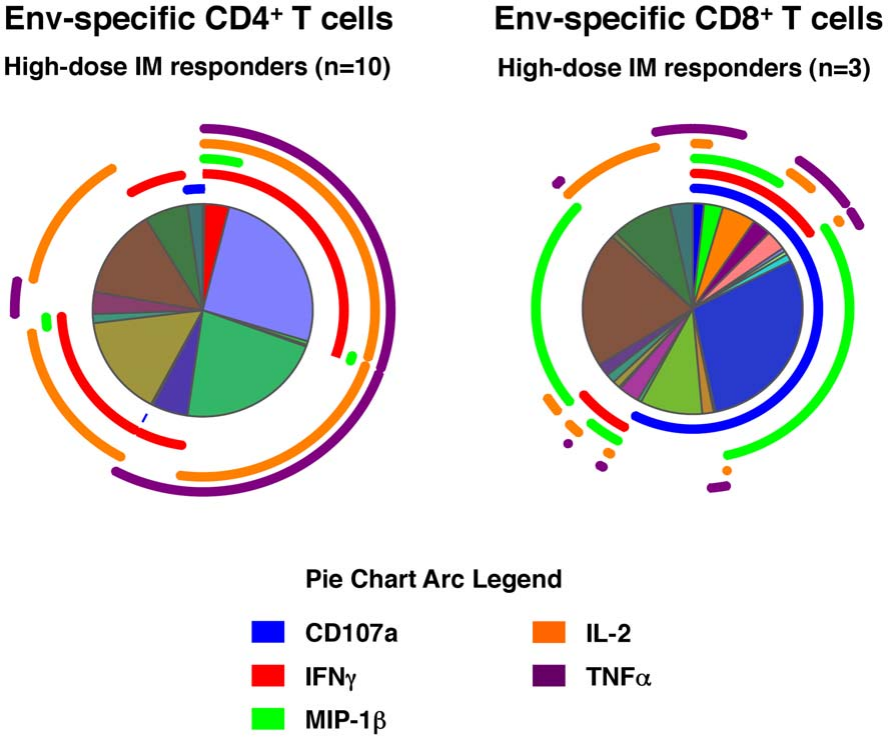
Multifunctional flow cytometry analysis for HIV Env-specific T cells. Analysis was based on the cumulative positive cells in all Boolean subsets for all volunteers who were scored as positive by single cytokine analysis in the high-dose (10^8^ pfu) intra-muscular vaccine recipients (**Table 5**). The legend shows the Pie Chart arcs representing each cytokine (or function). Pie Chart wedges show the relative sizes of the subsets of cells expressing the combination of functions represented in the surrounding Pie chart arcs.

Responses were evaluated at one pre-vaccination time-point and one post-vaccination time-point (the predicted peak of immunogenicity Day 98; 2 weeks post the 3^rd^ vaccination) in the high- and low-dose IM groups and the high-dose ID group (30 vaccinees and 6 placebos). The gating strategy for the multiparameter flow cytometry is shown in **supplementary Figure S3**. Overall responses were predominantly to the Env peptide pool and CD4^+^ T cell mediated (22/30 Env responders versus 11/30 Gag responders by any single cytokine/function). **Table 5** summarizes the responses against the Env peptide pool in each of the three groups tested. Responses against the Env peptides were dose and route-dependent: high-dose IM (10/10)>low-dose IM (7/10)>high-dose ID (5/10). In the high-dose IM group 100% of vaccine recipients exhibited CD4^+^ T cells producing IFNγ in response to the Env peptide pool. Eight of these 10 volunteers also responded to Env peptides by producing IL-2, and of these 4 volunteers also produced TNFα (1 volunteer exhibited production of MIP-1β in addition to the three cytokines). In the IM vaccine recipients IFNγ, TNFα and IL-2 dominated the CD4^+^ T cell response. Env-specific CD8^+^ T cells were detected in 3 volunteers – all in the high-dose IM group - and all exhibited CD107a surface translocation. Gag-specific CD4^+^ T cells were detected in 3 volunteers in each of the IM vaccine groups and 2 volunteers in the high-dose ID group. No Gag-specific CD8^+^ T cell responders were identified with this assay. A single false-positive response to IL-2 was detected in 1 of 6 placebo subjects who were tested. **Figure 5** shows the cumulative multifunctional analysis of the T cell responses detected against the Env peptide pool in the high-dose IM group. Responding CD4^+^ T cells were mono-functional (23%), dual functional (45%) or tri-functional (28%) producing IL-2, TNFα and/or IFNγ, while the CD8^+^ T cells were mono-functional (41%) and dual functional (39%), producing MIP-1β and translocated CD107a and/or produced IFNγ. The profiles of the Env- and Gag-specific CD4^+^ T cells were similar.

**Table 5:** Multi-Functional Flow Cytometry Results (Env peptide pool responses)*.

Dose/Route**	N		CD4^+^ T cells Env responses					CD8^+^ T cells Env responses				
			CD107a	IFNγ	MIP-1α	IL-2	TNFα	CD107a	IFNγ	MIP-1β	IL-2	TNFα
10^7^ pfu IM	10	Pre	0	0	1	0	0	2	0	0	0	0
		Day 98	1	3	0	6	6	1	0	0	1	1
		Median%	0.02	0.03		0.06	0.05	0.02			0	0.04
		[Range]	[0.02]	[0.02–0.09]		[0.05–0.24]	[0.03–0.24]	[0.02]			[0.03]	[0.04]
10^8^ pfu IM	10	Pre	0	0	1	1	0	0	0	1	0	0
		Day 98	1	10	2	8	4	3	3	2	1	1
		Median%	0.02	0.06	0.025	0.09	0.09	0.05	0.04	0.26	0	0.04
		[Range]	[0.02]	[0.03–0.14]	[0.02–0.03]	[0.06–0.17]	[0.05–0.16]	[0.03–0.23]	[0.02–0.07]	[0.07–0.46]	[0.04]	[0.03]
10^7^ pfu ID	10	Pre	0	1	1	0	1	1	1	0	0	0
		Day 98	0	4	1	4	2	2	1	0	0	0
		Median%		0.03	0.04	0.04	0.03	0.09	0.03			
		[Range]		[0.02–0.05]	[0.04]	[0.02–0.04]	[0.03–0.04]	[0.02–0.15]	[0.03]			

*The number of positive responses for each function is shown (bolded) as measured at 2 weeks after the third vaccation (day 98). The quantitative values (median and range) represent the corrected value (test antigen - background) for the responses scored as positive.

**6 placebo recipients were tested in the same assay procedure with a two false positives scored for CD107a (one CD4^+^ and one CD8^+^) at a pre-vaccination visit.

### Humoral Immunogenicity

#### Binding antibodies

Volunteers were assessed for the presence of binding antibodies against HIV-1 CM243 gp120, HIV-1 IIIB p24 and the vector itself (anti-vaccinia antibodies) at three time-points – pre-vaccination, and 2 weeks and 6 months post-completion of the vaccination schedule (days 98 and 252; 2 weeks and 6 months post 3^rd^ vaccination respectively). **Table 6** summarizes the quantitative (GMT for each group) and qualitative analyses for the binding antibody responses. Binding antibodies against gp120 were generated in up to 90% of vaccine recipients (9/10 IM high dose) after 3 doses of MVA-CMDR. Binding antibodies against p24 were generated in 90–100% of vaccine recipients regardless of the route after 3 doses of MVA-CMDR. Quantitatively, the median GMTs for both gp120 and p24 antigens displayed a peak at 2 weeks post-completion of vaccination and a decline by 6 months post-vaccination. There was a statistically significant difference (*p*<0.05; Kruskal-Wallis test with Dunn's correction for multiple comparisons) in median GMT between both IM groups and the placebo group for gp120 and p24 at Day 98, but this difference was maintained only for the high-dose IM group at day 252. In the ID route this quantitative statistical difference held only for high-dose ID group at day 98. For both HIV antigens, the GMTs generated by the IM route exceeded the ID route quantitatively at both post-vaccination time-points. This difference was statistically different only when comparing the IM groups with low-dose ID group (*p*<0.05; Kruskal-Wallis test with Dunn's correction for multiple comparisons). Of note was the observation that all treatment groups showed a quantitative decrease in binding antibody GMTs at 6 months post-vaccination compared with 2 weeks post-vaccination (*p* = 0.02 for all groups; Wilcoxon ranked sign test). Qualitatively the diminution of antibody response rates by 6 months post-vaccination was most evident for gp120, but was also observed for vaccinia virus response rates. Anti-vaccinia binding antibodies were generated in 90–100% of vaccine recipients depending upon the dose and route of immunization. As expected base-line anti-vaccinia binding antibody responses were negative in all groups. At the 10^7^ pfu dose the IM and ID routes demonstrated qualitative and quantitative equivalency for generating anti-vaccinia binding antibodies. The anti-vaccinia binding antibodies showed a quantitative decrease by 6 months post-vaccination for all treatment groups as observed for the HIV antigens. Overall, the 10^8^ pfu dose delivered by the IM route was superior for generating binding antibodies against all three antigens tested.

**Table 6:** Geometric Mean Antibody Titers and Response Frequency to HIV Proteins and Vaccinia*.

Dose/Route	N	HIV-1 gp120 (CM243)			HIV-1 p24 (IIIB)			Vaccinia Virus		
		GMT (No. of Responders)			GMT (No. of Responders)			GMT (No. of Responders)		
		Pre-vacc	2 weeks post 3^rd^ vacc	6 months post 3^rd^ vacc	Pre-vacc	2 weeks post 3^rd^ vacc	6 months post 3^rd^ vacc	Pre-vacc	2 weeks post 3^rd^ vacc	6 months post 3^rd^ vacc
10^6^ pfu ID	10	39 (0/10)	57 (2/10)	30 (0/10)	26 (1/10)	299 (9/10)	65 (5/8)	50 (0/10)	186 (9/10)	67 (2/10)
10^7^ pfu ID	10	57 (2/10)	208 (7/10)	43 (1/10)	31 (2/10)	1343 (9/10)	109 (9/10)	50 (0/10)	657 (9/10)	66 (2/10)
10^7^ pfu IM	10	38 (1/10)	354 (8/10)	54 (1/10)	29 (1/10)	2907 (10/10)	434 (10/10)	50 (0/10)	543 (9/10)	111 (6/10)
10^8^ pfu IM	10	69 (3/10)	787 (9/10)	102 (4/10)	26 (0/10)	3427 (10/10)	412 (10/10)	50 (0/10)	2071 (10/10)	195 (9/10)
Placebo	8	44 (1/8)	29 (0/8)	34 (1/7)	29 (1/8)	29 (1/8)	25 (0/7)	50 (0/8)	50 (0/8)	50 (0/8)

*The cut-off for determining a positive response was the upper 99% confidence limit of the end-point titers for all pre-vaccination samples for each antigen.

Note: Responses were measured at one pre-vaccination time-point (Pre-vacc), the presumed peak of immunogenicity (2 weeks post 3^rd^ vaccination; Day 98) and tested for durability (6 months post 3^rd^ vaccination; Day 252).

#### ADCC activity

Volunteers were assessed for ADCC activity at two time-points: pre-vaccination; and 2 weeks post-vaccination (day 98). MVA-CMDR vaccination elicited modest ADCC activity as shown in **Table 7**, with the greatest frequency of responders to both CRF01_AE (40%) and subtype B gp120 (30%) observed in vaccinees receiving the highest dose (10^8^ pfu IM). The median (inter-quartile range) %RL in this group to CRF01_AE and MN gp120 was 16.5% (5.0–42.6) and 8.8% (7.0–28.0), respectively, compared to 10.6% (3.6–19.3) and 2.8% (2.0–4.2) respectively, in the placebo group.

**Table 7:** Cumulative frequency of post-vaccination positive ADCC responses.

Dose/Route	N	CRF01_AE gp120*	Subtype B gp120*
10^6^ pfu ID	9	0 (0%)	1 (11%)
10^7^ pfu ID	9	1 (11%)	2 (22%)
10^7^ pfu IM	10	1 (10%)	2 (20%)
10^8^ pfu IM	10	4 (40%)	3 (30%)
Placebo	8	1 (12%)	1 (12%)

*Positive ADCC responses to CRF01_AE and subtype B gp120 proteins based on a cut-off of the 90th percentile relative lysis in the placebo group.

## Discussion

MVA-CMDR was safe and well tolerated at all dosages and routes tested. There were no serious adverse events related to the vaccine and there was no evidence of cardiac toxicity. MVA-CMDR elicits a modest, yet readily detectable cellular immune response against the HIV insert gene products in most vaccine recipients. Using traditional assay platforms, such as lymphocyte proliferation or chromium release assays, up to 100% of the volunteers had detectable lymphoproliferative responses (depending upon dose and route) and two-thirds (63%) had CD8-dependent CTL activity depending upon the dose and route. These data are consistent with the priming of both CD4^+^ and CD8^+^ T cells. Using more sophisticated flow cytometry-based assay techniques the balance and frequency of the cellular immune response is further elucidated. CD4^+^ T cells, in particular those targeting the Env protein, were most frequently detected with both whole blood ICS and multi-functional ICS. The high-dose IM vaccination regimen resulted in the induction of CD4^+^ T cells targeting Env in almost all volunteers using both ICS assays. However, CD8^+^ T cells specific for Env were detected in only 30% of the same volunteers using the two assay platforms. The whole blood ICS assay appeared to be more sensitive for detecting Gag-specific CD8^+^ T cells – 40% versus no positive responses with the multifunctional assay in the high-dose IM group. This observation may represent an important qualitative difference in sensitivity between the two assay platforms. The detection of Env-specific CD4^+^ T cells that synthesized IL-2, IFNγ and TNFα by flow cytometry is consistent with the lymphocyte proliferation data. The sporadic detection of insert-specific CD8^+^ T cells by ^51^Cr-release and flow cytometry is consistent with the induction of a low frequency of CD8^+^ T cells with high proliferative capacity. The durability of the cellular immune response is reflected in the 100% lymphoproliferative response rates in both IM route groups to the TH023 recombinant protein at 6 months after completion of the vaccination schedule and in the detection of Elispot positive responses at the same time-point. Overall, Env was consistently the predominant target of the cellular immune response and CD4^+^ T cells were the most frequently detected responder cell type. A dose and route effect of the vaccination procedure was observed with the high-dose (10^8^ pfu) IM route of vaccine delivery being the most immunogenic.

The observed trend towards elicitation of numerically greater CD4^+^ T cell responses targeting HIV-1 Envelope by the vaccine is an important observation. In humans, the use of recombinant multigenic MVA as a vector for both priming and boosting immune responses appears to be similar to multigenic DNA vaccines, which also induce primarily CD4^+^ T cell responses against Env-derived gene products [17], [18], [19], [20]. Both the present study and the EV02 study of a NYVAC-C vaccine product by the EuroVacc Consortium suggest that vaccination with vaccinia-derived poxvirus vectors may be equivalent in their ability to induce predominantly Env-specific CD4^+^ T cells with multigenic products [20]. While canarypox-based vaccines have been shown to induce CD4^+^ T cells responses against Envelope antigens in a multigenic canarypox-prime and protein-boost setting, it is as yet unclear whether the phenomenon of Env-specific CD4^+^ T cell dominance can be generalized to all multigenic poxvirus vectors [5], [21], [22]. In contrast, adenovirus-based vectors used either as a prime and boost, or as a boost for a DNA vaccine prime, appear to generate higher levels of cellular immune responses in general and generate a better balance of CD4^+^ and CD8^+^ T cell responses to multiple gene products in a multigenic setting [12], [23], [24]. While studies performed in the setting of HIV-1 infection have shown that robust, polyfunctional CD8^+^ T cell responses targeting multiple epitopes in the Gag (and in some cases Nef) protein are associated with better clinical outcome and protection from disease progression it is unclear whether such cells would also provide protection from infection [4], [25], [26], [27], [28]. In fact, in the setting of a vaccine that induced Gag- and Nef-specific CD4^+^ and CD8^+^ T cell responses no protection from HIV-infection was conferred [29], [30]. Hence, the functional relevance of Env (and Gag) specific CD4^+^ and CD8^+^ T cells in setting of vaccination for prevention of infection needs to be further explored. In particular, subtle differences in the qualities of T cells generated in response to different vector-based delivery combinations of multigenic gene products should be the focus of future studies. The ability to generate T cells that either help appropriate antibody class switching and affinity maturation, or act as direct effector cells, or both, will be a critical quality of any effective HIV vaccine.

Binding antibody responses were detected against both p24 and gp120. A clear dose- and route-dependence of the binding antibody response was evident with the IM routes being most immunogenic. Antibody titers peaked two-weeks after vaccination and waned, but were still detectable at 6 months post-vaccination. The diminution of binding antibody GMTs in the interim between 2 weeks and 6 months post-vaccination completion may be important with respect to poxvirus vectors in general. In the ALVAC-HIV/AIDSVAX B/E Phase III trial (RV144) the modest protective efficacy appeared be transient during the 6 months immediately post-vaccination and then waned. If induction of transient antibody responses is typical of non-replicating poxvirus vectors in general then strategies that increase the durability of such responses will need to be established. MVA-CMDR would therefore be an ideal candidate for testing different prime/boost strategies that will increase the durability of antibody responses. These antibodies likely had functional capacity as measured by ADCC activity in four volunteers in the high-dose IM group following three immunizations with MVA-CMDR. Previously, 4 immunizations with canarypox (vCP1521) expressing CRF01_AE gp120 had failed to induce ADCC activity [16], implying that MVA-CMDR is a more potent inducer of ADCC than canarypox vectors expressing CRF01_AE antigens. It is unknown whether such antibody responses would, on their own, be sufficient to mitigate transmission or to reduce viral set point and/or modulate disease progression.

Compared with other stand alone MVA-based HIV vaccine products MVA-CMDR would appear to be at least as immunogenic as the ADMVA [31] and the TBC-M4 [32] vaccine products (at a 2.5 fold lower dose than either) and substantially more immunogenic than the HIVA vaccine product [33]. MVA-CMDR also appears to be at least as immunogenic as the related attenuated poxvirus vector NYVAC product vP2010 [17]. For HIV and other infectious diseases, it has become increasing commonplace to use rMVA and NYVAC products to augment DNA primed cellular and humoral responses [20], [34], [35]. MVA-CMDR has been shown previously to be an effective boost for cellular immune responses generated by a heterologous DNA vaccine prime [9]. When used as stand-alone products recombinant poxvirus vectors induce quantitatively lower insert-directed cellular immune responses compared with recombinant Adenovirus-based vectors [12], [23], [30], [36]. Their continued use as HIV vaccine modalities is likely to be contingent upon: (i) successful pairing with other vectors in prime-boost regimens to increase the magnitude of the immune response; (ii) verification that the immune responses generated are qualitatively different to those generated by alternative vectors and (iii) successful demonstration of vaccine-induced protection in primate lentivirus challenge models.

MVA-CMDR was designed to be administered in combination with other vaccine products - either as the prime for a prime/boost poxvirus followed by recombinant protein vaccine schedule, as performed in the recently completed RV144 trial, or in combination with other vaccines such as DNA-based or Adenovirus-based HIV vaccines. Given that DNA-prime and MVA-boost vaccinations are superior to MVA alone vaccinations in animal models and in human trials [33], [34], [37], [38], [39], and the fact that MVA-CMDR has already been used successfully to boost a heterologous DNA-based vaccine product in humans [9] it is likely that this product will prove useful as a boost for cellular responses induced by other heterologous priming modalities. In addition, the versatility of MVA-CMDR may be further explored by exploiting its capacity to prime for antibody responses in prime/boost strategies that further test the concepts arising from the RV144 trial [5].

## Supporting Information

Figure S1 Cumulative CD8-dependent CTL responses. Cumulative responses determined by the ^51^Cr-release assay are shown for HIV-specific (any Env/Gag/Pol) responses (panel A) and for vector-specific (MVA) responses (panel B). The y-axis represents the cumulative response rate in percentage for each route and dose, while the time post-vaccination initiation (days) is shown on the x-axis. Red arrows denote the timing of the vaccination series.
(0.03 MB PDF)

Figure S2 Quantitative IFNγ Elispot responses for all doses and routes. Elispot counts are shown for the total peptide response (Env plus Gag plus Pol responses; panels A and B), for the Env peptide response (panels C and D) and the Gag peptide response (panels E and F). Panels A, C and E show the intra-muscular vaccination responses, while panels B, D and F show the intra-dermal vaccination responses. The y-axis represents the magnitude of the response (IFNγ SFC/10^6^ PBMC), while the time post-vaccination initiation (days) is shown on the x-axis. Data is presented as corrected values (test article - background). The dotted line (27 SFC/10^6^ PBMC) represents the limit of detection for the validated ELISPOT assay and the red arrows denote the timing of the vaccination series.
(0.10 MB PDF)

Figure S3 Gating strategy applied to the multifunctional flow cytometric analyses. A representative sample is shown for the gating strategy used for viable, CD3+ lymphocyte identification and subsequent subdivision into CD4+ and CD8+ T cells (panel A). A functional positive response is shown for both CD4+ T cells (IL-2 and TNFα) and CD8+ T cells (CD107a and MIP-1β) in response to the CM235 Env peptide pool (panel B).
(0.22 MB PDF)

Protocol S1 Trial Protocol.
(0.75 MB PDF)

Checklist S1 CONSORT Checklist.
(0.23 MB DOC)
