# Supplementary figures and images for: Phase I Safety and Immunogenicity Evaluation of MVA-CMDR, a Multigenic, Recombinant Modified Vaccinia Ankara-HIV-1 Vaccine Candidate
Source: PLoS One. 2010 Nov 15;5(11):e13983. doi: 10.1371/journal.pone.0013983 (PMC2981570; doi:10.1371/journal.pone.0013983)

## A Any Insert-Specific CD8 CTL Response

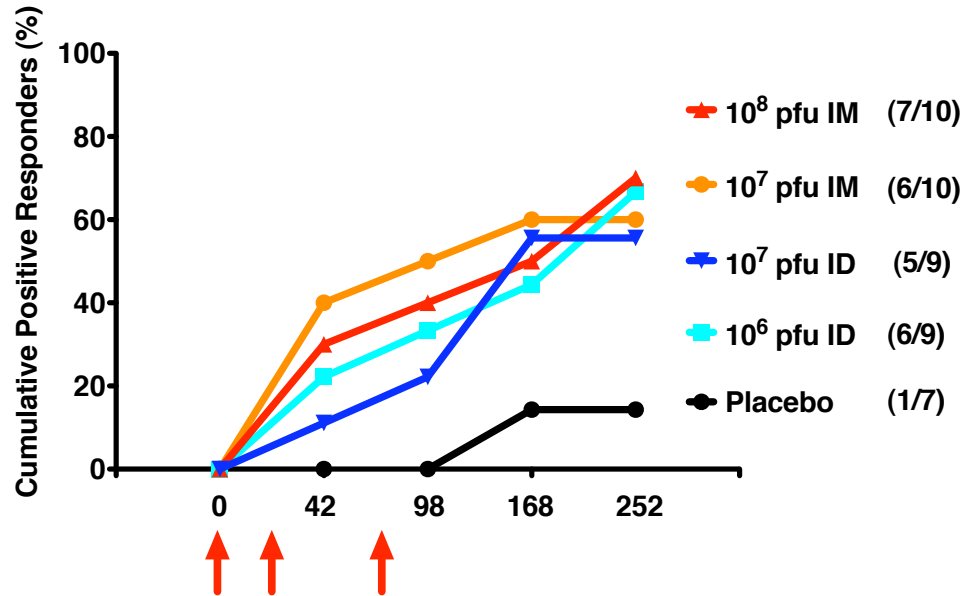

## B MVA-specific CD8 CTL Response

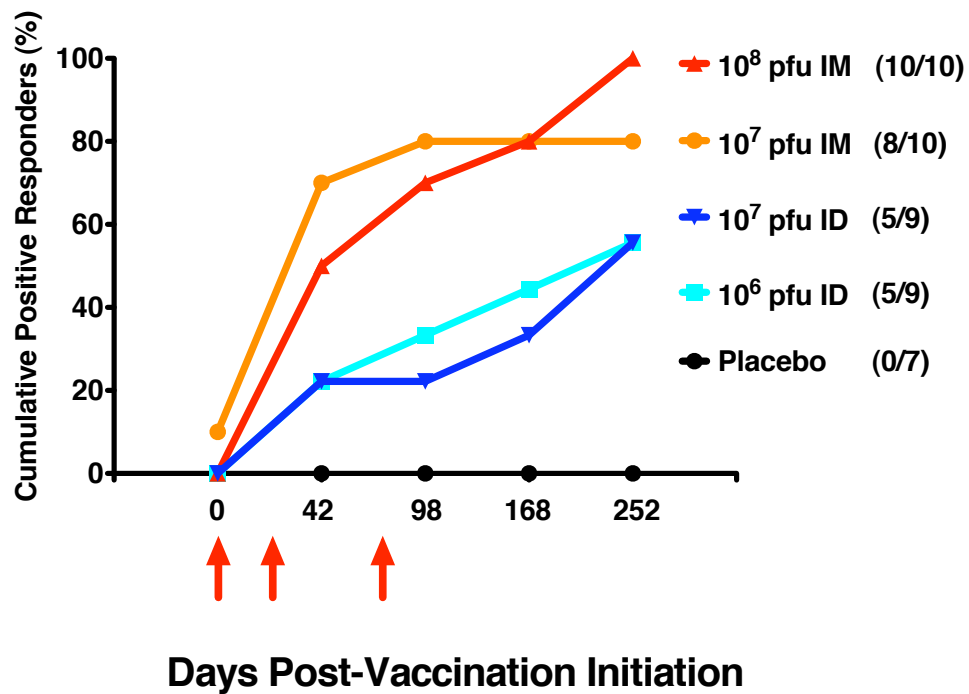

Supplement: Figure S1 — Cumulative CD8-dependent CTL responses. Cumulative responses determined by the 51Cr-release assay are shown for HIV-specific (any Env/Gag/Pol) responses (panel A) and for vector-specific (MVA) responses (panel B). The y-axis represents the cumulative response rate in percentage for each route and dose, while the time post-vaccination initiation (days) is shown on the x-axis. Red arrows denote the timing of the vaccination series. (0.03 MB PDF) [file pone.0013983.s001.pdf]

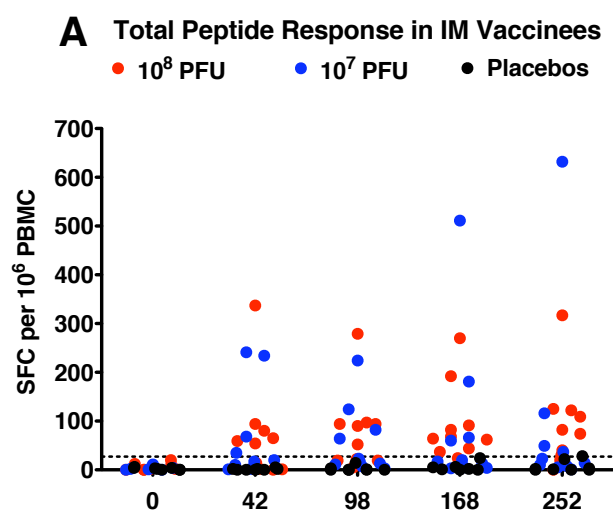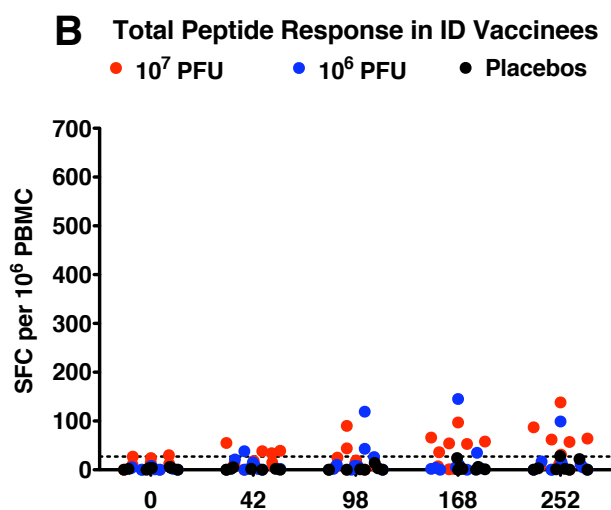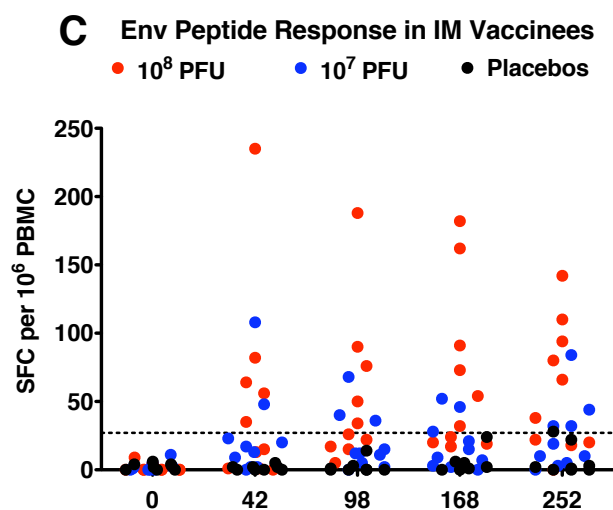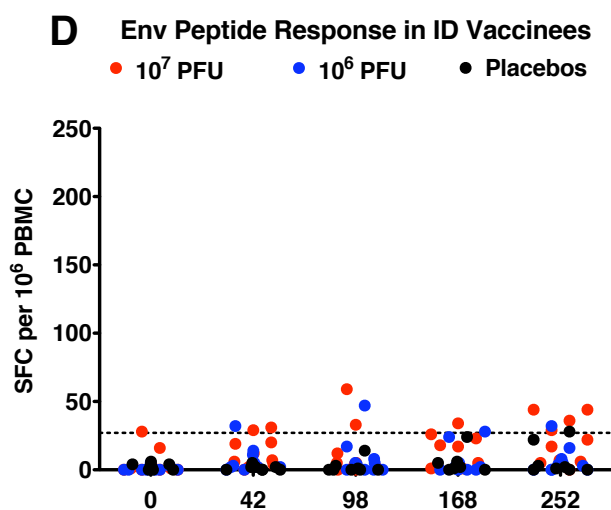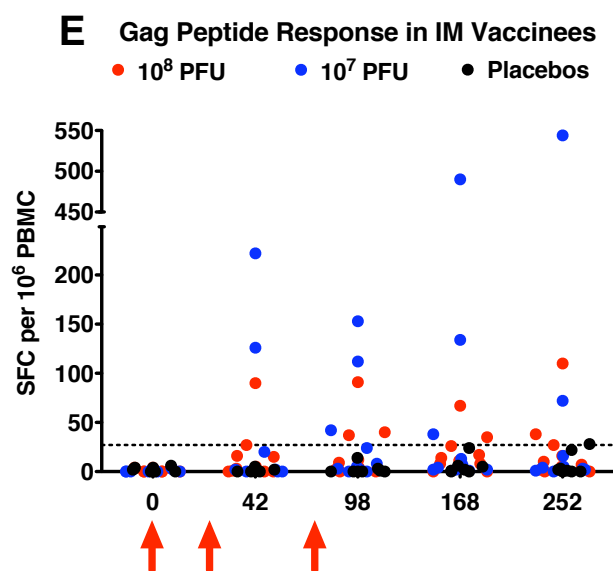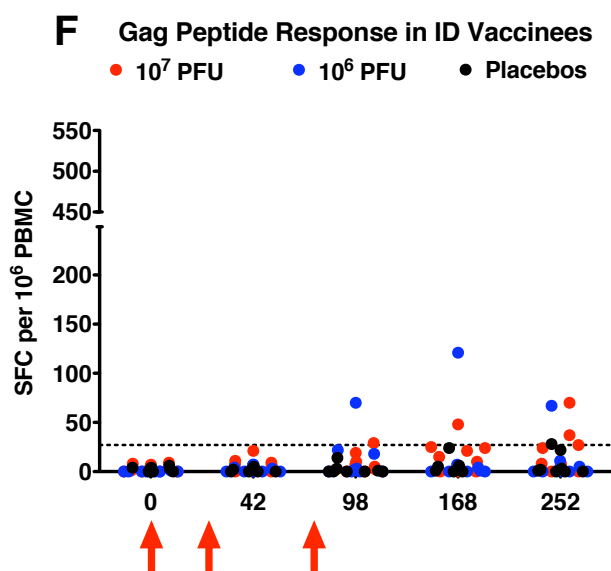

Days Post-Vaccination Initiation

Supplement: Figure S2 — Quantitative IFNγ Elispot responses for all doses and routes. Elispot counts are shown for the total peptide response (Env plus Gag plus Pol responses; panels A and B), for the Env peptide response (panels C and D) and the Gag peptide response (panels E and F). Panels A, C and E show the intra-muscular vaccination responses, while panels B, D and F show the intra-dermal vaccination responses. The y-axis represents the magnitude of the response (IFNγ SFC/106 PBMC), while the time post-vaccination initiation (days) is shown on the x-axis. Data is presented as corrected values (test article - background). The dotted line (27 SFC/106 PBMC) represents the limit of detection for the validated ELISPOT assay and the red arrows denote the timing of the vaccination series. (0.10 MB PDF) [file pone.0013983.s002.pdf]
