# Supplementary material for: Phase I Safety and Immunogenicity Evaluation of MVA-CMDR, a Multigenic, Recombinant Modified Vaccinia Ankara-HIV-1 Vaccine Candidate
Source: PLoS One. 2010 Nov 15;5(11):e13983. doi: 10.1371/journal.pone.0013983 (PMC2981570; doi:10.1371/journal.pone.0013983)

A. Gating strategy for light scatter/viability/dump channel for identifying CD3⁺ lymphocytes

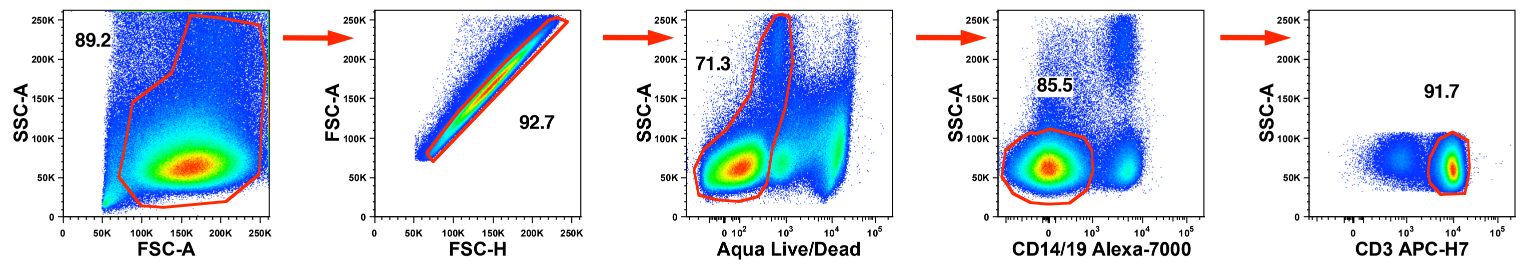

B. CD3⁺ T cell analysis

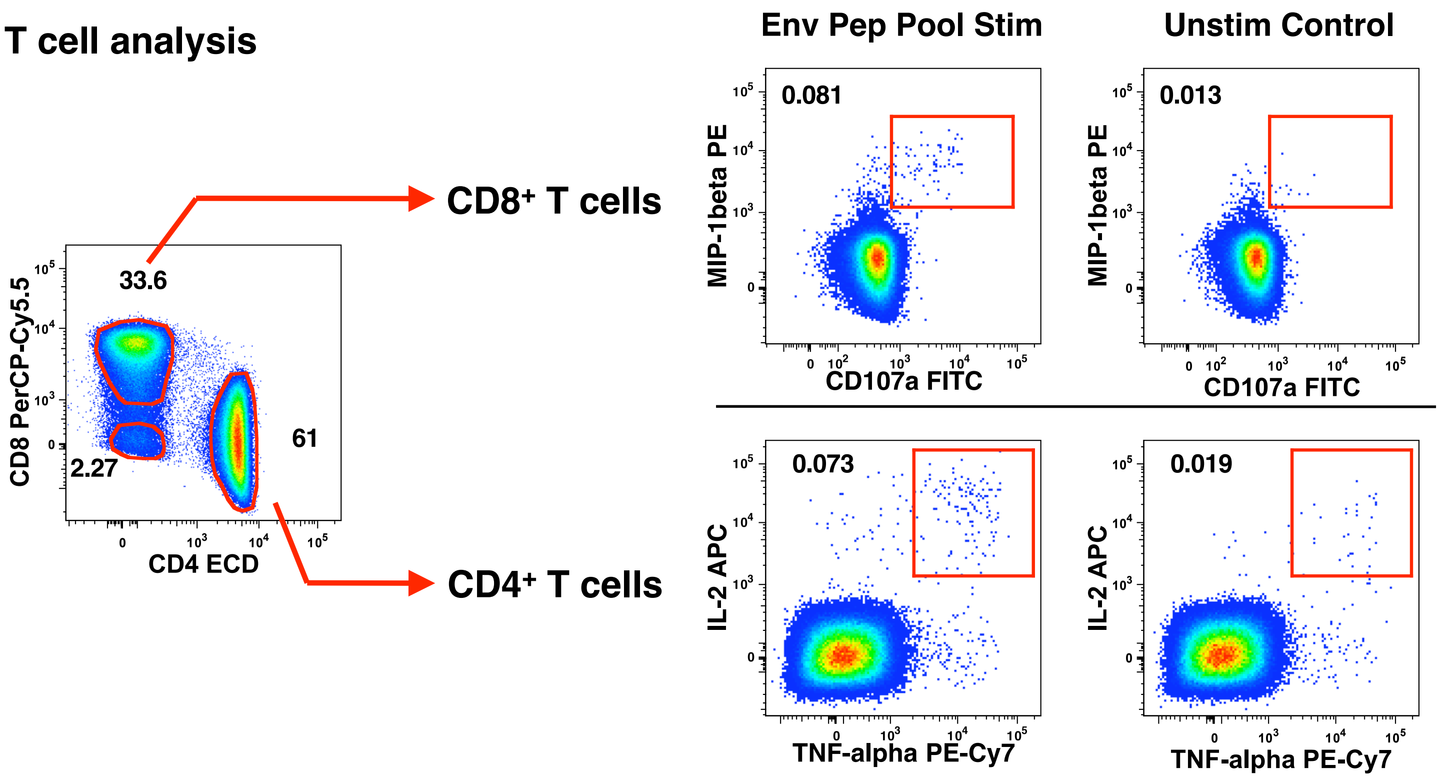

Supplement: Figure S3 — Gating strategy applied to the multifunctional flow cytometric analyses. A representative sample is shown for the gating strategy used for viable, CD3+ lymphocyte identification and subsequent subdivision into CD4+ and CD8+ T cells (panel A). A functional positive response is shown for both CD4+ T cells (IL-2 and TNFα) and CD8+ T cells (CD107a and MIP-1β) in response to the CM235 Env peptide pool (panel B). (0.22 MB PDF) [file pone.0013983.s003.pdf]
