# Supplementary material for: Phase I Safety and Immunogenicity Evaluation of MVA-CMDR, a Multigenic, Recombinant Modified Vaccinia Ankara-HIV-1 Vaccine Candidate
Source: PLoS One. 2010 Nov 15;5(11):e13983. doi: 10.1371/journal.pone.0013983 (PMC2981570; doi:10.1371/journal.pone.0013983)
Supplement: Protocol S1 — Trial Protocol. (0.75 MB PDF) [file pone.0013983.s004.pdf]

Version 2.2(WD)
10 MARCH 2008

Protocol RV 158

A Phase I Double-Blind, Randomized, Dose Escalating, Placebo-Controlled, Study of Safety and Immunogenicity of WRAIR/NIH Live Recombinant MVA-CMDR (HIV-1 CM235 env/ CM240 gag/pol) Administered by Intramuscular (IM) or Intradermal (ID) Route In HIV-Uninfected Adults (WRAIR Protocol # 1143)

Vaccines Provided by:
Walter Reed Army Institute of Research (WRAIR)
Forest Glen Section, Building 501
Department of Biologics Research
The Pilot Bioproduction Facility
Silver Spring, MD 20910

Vaccine study conducted by:
U.S. Military HIV Research Program

IND Sponsored by:
Office of the Surgeon-General
Department of the Army
U.S. Army Medical Materiel Development Activity
622 Neiman Street
Fort Detrick, MD 21702
BB IND # 12267

Principal Investigator: Mary A. Marovich, M.D., DTM&H

Thai Investigators: Prasert Thongcharoen, M.D., Somchai Sriplienchan, M.D. Jintanat Anaworanich,M.D.

WRAIR Investigators: Viseth Ngauy, M.D., Otha Myles M.D.

Henry M. Jackson Foundation (HMJF) Investigator: Merlin Robb, MD

U.S. Medical Monitor: Paul Scott, M.D, MPH (WRAIR)

Thai Medical Monitors: Krisada Jongsakul, M.D. (AFRIMS) and

Winai Ratanasuban, M.D. (Siriraj)

Division of AIDS (DAIDS), NIAID, Medical Officer: Massimo Cardinali, M.D.

TAVEG Protocol Steering Committee: Chair, Professor Prasert Thongcharoen Members: COL Sorachai Nitayaphan, M.D., PhD; LTC Jerome H. Kim, M.D.; Assoc. Professor Punnee Pitisuttithum, MBBS

SIGNATURE PAGE

A Phase I Double-Blind, Randomized, Dose Escalating, Placebo-Controlled, Study of Safety and Immunogenicity of WRAIR/NIH Live Recombinant MVA-CMDR (HIV-1 CM235 env/ CM240 gag/pol) Administered by Intramuscular (IM) or Intradermal (ID) Route In HIV-Uninfected Adults

I have read the foregoing protocol and agree to conduct the study as outlined. In addition, I agree to conduct the study in compliance with all applicable regulations and guidelines as stated in the protocol and other information supplied to me.

____

Signature and printed name of Principal Investigator **Date**

Contact Information:
Principal Investigator:
Mary A. Marovich, M.D., DTM&H
USMHRP, Rockville MD
Phone: 301-251-8337
Fax: 301-762-4177
Email: mmarovich@hivresearch.org

Clinical Investigators:
Merlin Robb, M.D.
USMHRP, Rockville MD, 20850
Phone: 301-251-8302
Fax: 301-762-7460
Email: mrobb@hivresearch.org

MAJ Viseth Ngauy, USAF, MC
Division of Retrovirology
Walter Reed Army Institute of Research
13 Taft Court
Rockville, MD 20850
Phone: 301-251-8328
FAX: 301-762-4177
E-mail: vngauy@hivresearch.org

MAJ Otha Myles, M.D.
WRAIR, Rockville, MD 20850
Phone: 301-251-8361
Fax: 301-251-8321
Email: omyles@hivresearch.org

Somchai Sriplienchan, M.D.
10/336 Junyanivej, Jangwatana Road
Pakklet, Nonthaburi 11120 Thailand
Phone: 6608814321400
FAX: 02 644 4824Email:sriplien@loxinfo.co.th

Dr. Jintanat Anaworanich,M.D.
315/6 Rajvithi Raod
Bankok, 10400 Thailand
Phone: 02-644-4888
FAX: : 02 644 4824
Email: jintanata@afrims.org

Prasert Thongcharoen, M.D
Department of Microbiology
Faculty of Medicine Siriraj Hospital
Mahidol University
2 Pran Nok Road
Bangkok Noi, Bangkok Thailand 10700
Phone: 66-2-866-2574
Fax: 66-2- 419-8406
Email: siptc@mahidol.ac.th

Study Coordinators:
Yolanda Lewis, MSN
USMHRP, Rockville MD 20850
Phone: 301-251-8350
Fax: 301-251-8321
Email: yllewis@hivresearch.org

Lei Zhu, RN
USMHRP, Rockville MD 20850
Phone: 301-251-8350
Fax: 301-251-8321
Email: lzhu@hivresearch.org

DAIDS Medical Officer:
Massimo Cardinali, M.D.
NIH/DAIDS, Rockville, MD
Phone: 301-451-2736
Fax: 301-402-3684
Email: mcardinali@niaid.nih.gov

Site And Data Monitoring:
USAMMDA (U.S. Army Medical and Materiel Development Activity) Data Manager:
Mark Milazzo
Data Coordinating and Analysis Center
USMHRP, Rockville, MD 20850
Phone: 301-251-5025
Fax: 301-294-1898

US Medical Monitor:
Paul Scott, M.D, MPH
WRAIR, Rockville MD, 20850
Phone: 301-251-8339
Fax: 301-762-7460
Email: pscott@hivresearch.org

Thai Medical Monitors:
Dr. Krisada Jongskul
Dept. of Immunology & Medicine, AFRIMS
315/6 Rajvithi Road
Bangkok, 10400
Phone: 02644 4888 ext. 2684
Email: kusadaj@afrims.org

Winai Ratanasuwan, MD, MPH
Dept. of Preventative & Social Medicine
Faculty of Medicine, Siriraj Hospital
Bangkok, 10700
Phone: 622-411-3019, 419-7284
FAX: 622-411-5034
Email: srwrt@mahidol.ac.th

Laboratory Investigators:
Jeffrey Currier
USMHRP, Rockville, MD 20850
Phone: 301-251-8311
FAX: 301-762-4177
Email: jcurrier@hivresearch.org

Victoria Polonis, PhD
USMHRP, Rockville, MD 20850
Phone: 301-251-8308
Fax: 301-762-4177
Email: vpolonis@hivresearch.org

Vaccine Manufacturer:
Walter Reed Army Institute of Research
Forest Glen Section, Bld 501
Department of Biologics Research
The Pilot Bioproduction Facility
Silver Spring, MD 20919

Site Pharmacy Technicians:
Christine Bush
John Eggleston
Erik Odom
Lindsay Wieczorek
USMHRP, Rockville MD 20850

USMHRP Regulatory Operations Center (ROC):
Beryl Wessner, Pharm.D.
USMHRP, Rockville, MD 20850
Phone: 301-251-5037
Fax: 301-610-9135
Email: bwessner@hivresearch.org

Participating Laboratories:
Quest Diagnostics
1901 Sulphur Spring Road
Baltimore, MD 21227

HIV Diagnostics Laboratory
13 Taft Ct., Suite 100
Rockville, MD 20850

University of St. Louis
1034 S. Brentwood Blvd
St. Louis, MO 63117

Department of Retrovirology,
Armed Forces Research Institute of Medical Sciences,
315/6 Rajvithi Road, Bangkok, Thailand 10400

Bumrungrad International Laboratory
33 Sukhumvit 3, Bangkok, Thailand 10110

Bangkok RIA Lab Co. Ltd.
6 Ladpraw Rd, Bangkok 10310

TABLE OF CONTENTS

NUMBER
PAGE

LIST OF ABBREVIATIONS AND DEFINITIONS OF TERMS 7

PRÉCIS 11

1 INTRODUCTION AND RATIONALE 14

1.1 GENERAL BACKGROUND 14
1.2 THE MODIFIED VACCINIA ANKARA (MVA) 15
1.3 PREVIOUS STUDIES WITH MVA CONSTRUCTS 16
1.3.1 Animal Studies 16
1.3.2 Human Experience with MVA 21
1.4. MVA-CMDR BACKGROUND AND DEVELOPMENT 22
1.4.1. Origin of MVA 23
1.4.2. Origin of HIV GAG/POL and ENV Genes 23
1.4.3. Production of rMVA Viruses 24
1.4.3.1. Preparation of Bulk Lot, Final Product, and Diluent/Placebo 25
1.4.3.2 In Vitro Tissue Culture Safety (#AA64KV.003000.BSV) 27
1.5 RATIONALE FOR A CLINICAL TRIAL WITH MVA ENV/GAP/POL SUBTYPE E/A 31
1.6 RATIONALE FOR THE VACCINATION SCHEDULE AND STUDY GROUPS 32
1.7 HUMAN EXPERIENCE TO DATE 34

2 PURPOSE OF THE STUDY 34

2.1 PRIMARY OBJECTIVE 34
2.2 SECONDARY OBJECTIVE 34

3 STUDY DESIGN 35

3.1 STUDY POPULATION 36
3.2 INCLUSION CRITERIA 38
3.3 EXCLUSION CRITERIA 39
3.4 CONCOMITANT MEDICATIONS 40
3.5 PRECAUTIONARY MEDICATIONS AND PROCEDURES 40
3.6 SCHEDULE OF CLINICAL PROCEDURES AND LABORATORY ASSAYS 41
3.6.1 Screening 41
3.6.2 Day 0 Through Day 252 Follow-Up 43
3.6.3 Day –90 to -14 44
3.6.4 Day –28 to –3 45
3.6.5 Vaccinations (Days 0, 28, and 84) 45
3.6.6 Twenty-four to Forty-eight Hour Contact Post Each Vaccination: 46
3.6.7 Safety Evaluations 46
3.6.8 Day 168 (+/-14 days): 46
3.6.9 Study Termination Visit, Day 252 (+/- 14 days) 47
3.7 LABORATORY RESULT FOLLOW-UP VISIT 11, DAY 280 (+/- 7 DAYS) 47

4 STUDY MANAGEMENT 47
4.1 MONITORING POST-VACCINATION EXPERIENCES THAT ARE POSSIBLE REACTIONS TO VACCINE/PLACEBO VACCINATION 47
4.1.1 Post-vaccination Reactions Occurring Immediately After Vaccination 48
4.1.2 Local Reactions Within Seven Days Post-vaccination 48
(Study Days 0 to 6) 48
4.1.3 Systemic Reactions Within 7 Days Post-vaccination 48
4.1.4 Post-vaccination Contact 49
4.1.5 Instructions to Volunteers Regarding Unusual or Severe Signs or Symptoms 49
4.2 MONITORING FOR HIV INFECTION 49
4.3 MANAGEMENT OF VOLUNTEERS WHO BECOME HIV-INFECTED DURING THE STUDY 50
4.4 CRITERIA FOR WITHDRAWAL OF VOLUNTEERS FROM VACCINATION SCHEDULE 50
4.5 OBTAINING REPLACEMENT VIAL ASSIGNMENT 51
5 PHARMACOVIGILENCE, SAFETY AND ADVERSE EXPERIENCE REPORTING 52
5.1 PHARMACOVIGILENCE AND CRITERIA FOR STOPPING STUDY 52
5.2 ADVERSE EXPERIENCES 55
5.3 SERIOUS ADVERSE EXPERIENCES 56
5.4 ADVERSE EXPERIENCE REPORTING 57
5.5 SERIOUS ADVERSE EXPERIENCE REPORTING 57
5.5.1 Regardless of assessed relationship to study agent, report 58
5.5.2 Up to six months after last dose of study agent, report 58
5.6 ADVERSE EXPERIENCE REPORTING TO THE INSTITUTIONAL REVIEW BOARD 58
5.6.1 Local Medical Monitor 58
5.6.2 Reporting Serious and Unexpected Adverse Experiences to the US Army IRB 59
6 STATISTICAL CONSIDERATIONS 60
6.1 STUDY OVERVIEW 60
6.2 PRIMARY AND SECONDARY ENDPOINTS 61
6.2.1 Primary Endpoints, Safety 61
6.2.2 Secondary Endpoints, Immunogenicity 61
6.2.2.1.1 Humoral 61
6.2.2.1.2 Cellular 61
6.3 SAMPLE SIZE, ACCRUAL, AND DOSE-ESCALATION GUIDELINES 61
6.4 STATISTICAL PLAN: DESIGN & ANALYSIS 63
6.5 SAFETY 63
6.5.1 Post vaccination Reactions (Local and Systemic) 63
6.5.2 Other Adverse Experiences 64
6.5.3 Clinical Laboratory 64
6.5.4 Immunogenicity 64
6.6 DATA MONITORING 65
7 PHARMACY PROCEDURES 66
7.1 PROTOCOL SCHEMA 66
7.2 VACCINE DESCRIPTION AND PREPARATION 66
7.3 PLACEBO DESCRIPTION AND PREPARATION 68
7.4 STUDY PRODUCT ACQUISITION/DISPOSITION 68

7.5 PHARMACY RECORDS 69
7.6 PROCEDURES TO PRESERVE BLINDING 69

8 HUMAN SUBJECT PROTECTIONS AND ETHICAL OBLIGATIONS 71

8.1 INFORMED CONSENT 71
8.2 RISKS AND BENEFITS 72
8.2.1 Risks 72
8.2.2 Benefits 73
8.3 SUBJECT CONFIDENTIALITY 74
8.4 VOLUNTEER REGISTRY DATABASE 75
8.5 PARTICIPATION OF CHILDREN 75
8.6 COMPENSATION 75

9 ADMINISTRATIVE AND LEGAL OBLIGATIONS 76

9.1 DESIGNATION OF ROLES AND RESPONSIBILITIES 76
9.2 PROTOCOL AMENDMENTS AND STUDY TERMINATION 76
9.3 STUDY DOCUMENTATION AND STORAGE 77
9.4 STUDY MONITORING AND DATA COLLECTION 78
9.5 POLICY REGARDING RESEARCH-RELATED INJURIES 79
9.6 USE OF INFORMATION AND PUBLICATION 79
9.7 CONDUCT OF THE RESEARCH STUDY 80

10 REFERENCES 80

ATTACHMENT I INFORMED CONSENT FORM
ATTACHMENT II VOLUNTEER REGISTRY DATA SHEET
ATTACHMENT III TEST OF UNDERSTANDING
ATTACHMENT IV SCHEDULE OF EVALUATION
ATTACHMENT V TABLE FOR GRADING SEVERITY OF ADVERSE EXPERIENCES
ATTACHMENT VI ASSESSMENT OF VOLUNTEER RISK
ATTACHMENT VII INFORMATION AND BRIEFING SESSIONS
ATTACHMENT VIII EVENT SCHEDULE
ATTACHMENT IX LETTER OF PARTICIPATION
ATTACHMENT X ADVERTISEMENT
ATTACHMENT XI HSRRB REQUESTED INFORMATION

TABLE 1. LIST OF ABBREVIATIONS AND DEFINITIONS OF TERMS

ABBREVIATION/ACRONYM	DEFINITION
°C	Centigrade or Celsius
°F	Fahrenheit
Ab	Antibody
ACD	Acid citrate dextrose
AE	Adverse experience
AI	Associate Investigator
AIDS	Acquired immunodeficiency syndrome
ALT/AST	Alanine aminotranferease and aspartate aminotransferase liver enzymes
ALVAC-HIV	Canary pox Avipox vectored HIV vaccine candidate (ALVAC-HIV)
ANOVA	Analysis of variance
CBC	Complete blood count
CBER	Center for Biologics Evaluation and Research (FDA)
CCID50	Cell Culture Infective Dose (50% of cells infected)
CD4+	Helper T-Cells that heighten the production of antibodies by B-Cells and regulate the activities of all effector cells. A functional subclass of T-Cells that helps to generate $CD8^+$ Cells and cooperate with B-Cells in the production of antibody-mediated responses
CD8+	Cytotoxic T-Cells that destroy host cells, which have become infected by viruses or other intracellular pathogens
CEFC	Chick embryo fibroblast cells
CFR	Code of Federal Regulations
cGMP	Current Good Manufacturing Practices
CIOMS	Council of International Organization of Medical Sciences
CPE	Cytopathic effects
CPK	Phosphocreatine kinase
CPK-MB	Phosphocreatine kinase- Muscle-Brain
CPM	Counts per minute
CRF	Case report form
CRP	C Reactive Protein
CTL	Cytotoxic T lymphocyte

ABBREVIATION/ACRONYM	DEFINITION
DAIDS	U.S. Division of AIDS, NIAID, NIH
DCAC	Data Coordinating and Analysis Center
DNA	Deoxyribonucleic acid
DSMB	Data and Safety Monitoring Board
EBV	Epstein barr virus
ECG	Electrocardiogram
EDTA	Ethylenediaminotetraacetic acid
ELISA	Enzyme linked immunosorbent assay
ELISPOT	Enzyme-Linked Immunospot
FDA	U.S. Food and Drug Administration
FTA	Fluorescent treponemal antibody
GCP	Good Clinical Practices
GGT	Gamma-Glutamyl Transpeptidase
GLP	Good Laboratory Practices
GMT	Geometric mean titer
H&P	History and physical
HA	Hemagglutination
HAD	Hemadsorption
HBsAg	Hepatitis B surface antigen
HIV and HIV-1	Human immunodeficiency virus, type 1
HLA	Human leukocyte antigen
HMJF	Henry M. Jackson Foundation for the Advancement of Military Medicine
HSRRB	Human Subjects Research Review Board
HURC	Human Use Review Committee
IB	Investigator's Brochure
ICH	International Conference on Harmonization
ICS	Intracellular cytokine staining
ID	Intradermal injection
IgG	Immunoglobulin G
IM	Intramuscular injection
IN	Intranasal injection
INF-γ	Interferon gamma
INS	Inhibitory nucleotide sequences
IP	Intraperitoneal injection
IRB	Institutional Review Board
IVS	*in vitro* stimulation
LPA	Lymphocyte proliferation assay
LSI	Lymphocyte stimulation index
MeDRA	Medical Dictionary for Regulatory Activities; regulatory terminology for coding safety reports

ABBREVIATION/ACRONYM	DEFINITION
MHC	Major histocompatibility complex
mL	Milliliter
MO	Medical Officer
MVA-CMDR	Modified Vaccinia Ankara- Chiang Mai Double Recombinant
NAb	Neutralizing antibody
NIAID	National Institutes of Allergy and Infectious Diseases
NIH	National Institutes of Health
NYVAC	New York Vaccinia Virus Strain
ORP	Office of Research Protections
OTSG	Office of the Surgeon General
PBMC	Peripheral blood mononuclear cells
PBS	Phosphate buffered saline
PCR	Polymerase chain reaction
pfu	Plaque forming units (equivalent to International Units)
PI	Principal investigator
PIN	Personal identification number
RA	U.S. Army Medical Materiel Development Activity Office of Regulatory Affairs
RBC	Red blood cell count
rMVA	Recombinant MVA
RNA	Ribonucleic acid
RPR	Rapid plasma reagent
rVV	Recombinant Vaccina Virus
SAE	Serious adverse experience
SC	Subcutaneous injection
SCID	Severe combined immunodeficiency
SIV	Simian immunodeficiency virus
SP	Site Pharmacist
TAA	Thymidine-adenine-adenine
TAVEG	Thai AIDS Vaccine Evaluation Group
TB	Tuberculosis
TBD	To be determined
TCLA	Tissue culture laboratory adapted
U.S.	United States
UA	Urinalysis
USAMMDA	U.S. Army Medical Materiel Development Activity
USAMRMC	U.S. Army Medical Research and Materiel Command

ABBREVIATION/ACRONYM	DEFINITION
USMHRP-ROC	U.S. Military HIV Research Program-Regulatory Operations Center
VCRC	Vaccine Clinical Research Center
VPW	Vaccine Preparation Worksheet
VV	Vaccinia Vaccine
WB	Western blot
WBC	White blood cell count
WFI	Water for injection
WRAIR	Walter Reed Army Institute of Research

PRÉCIS

Protocol Title: A Phase I Double-Blind Randomized Dose Escalating, Placebo-Controlled, Study of Safety and Immunogenicity of WRAIR/NIH Live Recombinant MVA-CMDR (HIV-1 CM235 env/ CM240 gag/pol) Administered by Intramuscular (IM) or Intradermal (ID) Route In HIV-Uninfected Adults

Study Objectives: Primary: To evaluate the safety and tolerability of MVA-CMDR (HIV-1 CM235 ENV/ CM240 GAG/POL) administered by IM or ID injection to HIV-uninfected adult volunteers.

Secondary: To evaluate the ability of MVA-CMDR (HIV-1 CM235 ENV/ CM240 GAG/POL) to induce HIV antigen specific cellular and humoral immune responses.

Study Design:

Phase I double-blind, randomized, dose-escalating, placebo-controlled, study.

Product Description: MVA-CMDR, produced by WRAIR/NIH, is a live recombinant poxvirus vector (Modified Vaccinia Ankara) vaccine that has been genetically engineered to express the following HIV-1 genes: gp160 (Subtype E, CM235) and gag and pol (integrase-deleted and reverse transcriptase non-functional, Subtype A, CM240).

MVA-CMDR is formulated in a liquid form in vials containing either 10^7 (Lot # 0966) or 10^9 pfu (Lot # 0965) per 1 mL. The product will be administered either at a dose of 10^6, 10^7 or 10^8 pfu by intramuscular or intradermal routes following appropriate dilutions when needed.

Subjects: Healthy HIV-uninfected adult volunteers (18 to 40 years old at the time of enrollment).

Study Plan: Volunteers will be enrolled up to 90 days prior to the first vaccination. Volunteers will be randomized to vaccine or placebo in a 5:1 ratio (see Schema). For safety reasons, volunteers will be enrolled in a staggered fashion, as follows:

- Part A, Groups I and II receiving the lower doses will be enrolled first.
- Part B, Groups III and IV, receiving the higher doses will be enrolled after the first two vaccinations of Part A are shown to be safe.

For both Parts, vaccinations will be on Days 0, 28, and 84 of each volunteer's schedule; each vaccination will be followed with a phone call (within 24 to 48 hours) and a safety visit (within 14 days); and each subject will be followed up for approximately 40 weeks after the third vaccination.

TABLE 2. Study Schema

**Part A**	**Subjects**	**Vaccination Schedule**		
**Group**	**Vaccine/Placebo**[1]	**Day of the Study in Part A**		
		**0**	**28**	**84**
I	10/2	10^7 pfu IM, 1.0 mL into deltoid muscle		
II	10/2	10^6 pfu ID, 0.1 mL into the volar aspect of the forearm to produce a wheal		
**Review of Safety Data for First and Second Vaccinations of Part A Groups**				
**Part B**	**Subjects**	**Vaccination Schedule**		
**Group**	**Vaccine/Placebo**	**Day of the Study in Part B**		
		**0**	**28**	**84**
III	10/2	10^8 pfu IM, 1.0 mL into deltoid muscle		
IV	10/2	10^7 pfu ID, 0.1 mL into the volar aspect of the forearm to produce a wheal		

[1]Vaccine: MVA-CMDR ; Placebo: vaccine diluent consists of PBS (without Ca^{2+} and Mg^{2+}), with 7.5% lactose , pH 7.4.

Study Duration: 52 weeks for each participant.

Study Sites: US Military HIV Vaccine Development,
Vaccine Clinical Research Center
1600 E. Gude Drive, Rockville MD 20850.

Department of Microbiology
Faculty of Medicine, Siriraj Hospital, Mahidol University; and AFRIMS Clinical Trial Center, Bangkok, Thailand

Study Endpoints: Primary: Safety and tolerability. Tabulation of adverse effects and laboratory abnormalities; immediate local and systemic reactions (30 minutes post vaccine/placebo vaccinations); local and systemic reactions recorded on subjects' diaries (Six days post vaccine/placebo vaccination); routine hematology, serum chemistry, liver function tests, and urinalysis (14 days post vaccine/placebo vaccination); and follow-up period data (Six months post last vaccine/placebo vaccination). The last study visit is scheduled 8 months post last vaccination and will be laboratory follow up only. No assessments will be conducted.

Secondary: Immune responses in both compartments.

- Cellular: CTL responses against Subtype E and B env, gag, and pol targets expressed in (or loaded onto) EBV transformed autologous B cell lines; IFN-γ ELISPOT and intracellular cytokine (ICS) responses using a panel of env and gag E and B clade-derived peptides; and lymphocyte proliferation (LPA) to relevant clade A, E and B HIV antigens (env, gag and pol).

- Humoral: Serum binding (ELISA) antibodies to HIV-1 Subtype E and B envelope antigens, and serum binding (ELISA) antibodies Subtype A and B p24 antigens.

1 INTRODUCTION AND RATIONALE

1.1 General Background

The global pandemic of HIV/AIDS in 2004 remains out of control, most strikingly in sub-Saharan Africa, South and Southeast Asia, and in more recently developing epidemic zones including India, China, and Russia and the Former Soviet Union/Eastern Europe. Despite some successes in controlled prevention trials, recent advances in anti-retroviral therapy, and evidence of some epidemic control in at least two countries, Thailand and Uganda, new HIV-1 infections occur at a rate of approximately 15,000 per day. Ninety percent of these new infections occur in developing countries, as only a few of these countries have successfully implemented effective national programs to control ongoing HIV spread. The currently available prevention technologies have thus far not succeeded in achieving control in most of the world's most affected regions. With the adult population prevalence of HIV infection reaching over 30% in at least four African countries, and many more over 5%, it is clear that the HIV epidemic presents the most serious infectious disease threat to many populations worldwide. HIV preventive vaccines are needed to contain the global pandemic, especially in resource limited areas of the world[1, 2].

Several vaccine approaches have already been tested in animals and in humans. While subunit vaccines such as gp120 or gp160 antigens are able to elicit a strong humoral response, they have not usually induced an anti-HIV $CD8^+$ specific CTL response. In contrast, live recombinant pox vectors including vaccinia and canary pox virus constructs encoding HIV-1 genes can infect mammalian cells causing them to express HIV-1 proteins and cell-mediated immunity[3,4,65-71]. The combination of both approaches in prime-boost protocols was able to induce both humoral and cell-mediated responses including neutralizing antibodies against TCLA HIV-1 strains, lymphoproliferation and HIV-1 specific cytotoxic T-lymphocyte activity[5,6]. However, vaccinia virus is infectious for humans and can, although in rare cases, cause a severe disseminated vaccinia syndrome in immunocompromised subjects[7]. Its use in the laboratory and as a public health vaccine has been affected by safety and regulatory concerns. Low and high doses of other non-replicative live recombinant vectors such as canary pox Avipox vectored HIV vaccine candidate (ALVAC-HIV) constructs administered by intramuscular route have been shown to produce both antibody and lymphoproliferative responses in almost all subjects whereas CTL responses were detected in only 35% of HIV-uninfected volunteers[6].

These cellular responses were of limited duration and were intermittently detected using standard laboratory immunologic assays. These results have prompted researchers to develop new vaccine concepts and strategies, especially new safe live recombinant vectors that could elicit strong and durable CTL activity in humans.

The USMHRP is currently completing study RV138, #BB-9419, which compared alternate delivery routes of an ALVAC-HIV construct, i.e. intramuscular, intradermal and autologous vaccine loaded dendritic cells. For general vector applications, health risks would be lessened by the adoption of a highly attenuated vaccinia virus strain. Several such strains were developed for use as safer smallpox vaccines. One of these strains is the Modified Vaccinia Ankara or MVA.

1.2 The Modified Vaccinia Ankara (MVA)

The Modified Vaccinia Ankara (MVA) was derived from vaccinia virus strain Ankara (WT) by over 570 serial passages in chick embryo fibroblast cells (CEFC)[8-10]. The genome of MVA is 178 kb in length, significantly smaller than that of the vaccinia Copenhagen genome that is 192 kb. The 193 open reading frames mapped in the MVA genome probably correspond to 177 genes, 25 of which are split and/or have suffered mutations resulting in truncated proteins. The resulting MVA strain lost the capacity to productively infect mammalian cells and suffered seven major deletions of DNA, including at least two host-range genes (K1L and C7L)[11,12]. Host ranges of MVA and the parental Ankara strain were compared in CEF and 15 permanent cell lines[13]. For MVA, the permissive category consisted of primary CEF, a quail cell line derived from the QT6, and the Syrian hamster cell line BHK-21[14]. Only in BHK-21 cells did the virus yield approach that occurring in primary CEF. The semi-permissive category included two African green monkey cell lines. The nonpermissive category consisted in three human cell lines, HeLa, 293, SW 839, one rhesus monkey cell line, two Chinese Hamster Ovary cell lines, CHO and CHL, one pig cell line and three rabbit cell lines. The grouping for MVA with a restored K1L host range gene was similar except for one rabbit cell line. The permissive BHK-21 cell line was shown to be competent for constructing and propagating rMVA, providing an alternative to primary CEF. In HeLa and 293 cells, whereas the wild type vaccinia Ankara increased 10,000 fold in titer, MVA increase could not be detected. However, in a recent study, it was shown that MVA could replicate, albeit poorly, in transformed human cells (HeLa and tk^{-}143B), but not in primary human fibroblasts although there is limited cell-to-cell spread[15].

When MVA was tested in a variety of animal species, it proved to be avirulent even in the immunosuppressed animals. Contrary to expectations, the expression of late (six to 12 hours after infection), as well as early, viral genes was unimpaired in human cells despite the inability of MVA to produce infectious progeny. Despite the differences in the abilities of WT and MVA to multiply in human cells, viral DNA replication proceeds similarly. The block in virus assembly occurs after DNA replication. This desirable result was not predicted since a large number of open reading frames, including the K1L host-range gene, are impaired in MVA. Such a deletion in the Copenhagen strain of vaccinia virus resulted in abortive expression of early viral genes followed by a rapid inhibition of further viral and cellular protein synthesis. In addition, viral DNA replication was inhibited. Viral DNA replication was also severely inhibited in human cells infected with New York Attenuated Vaccinia Virus strain (NYVAC), a genetically engineered virus derived for recombinant vaccine purposes from the Copenhagen strain by deletion of multiple open reading frames including the K1L and a second host-range gene C7L[16]. Furthermore, naturally host range-restricted avipoxviruses cannot carry out DNA replication in mammalian cells. The results obtained with MVA are not totally unprecedented since some host-range white pock deletion mutants of rabbit poxvirus support DNA replication and expression of late protein in nonpermissive pig kidney cells. The rabbit poxvirus mutants, however, exhibited quantitatively decreased late protein synthesis in nonpermissive cells and were blocked at a stage after the proteolytic clevage stage.

1.3 Previous Studies with MVA Constructs

1.3.1 Animal Studies

Several animal studies have been conducted with various recombinant MVA constructs expressing non-retroviral (influenza, parainfluenza 3, respiratory syncytial virus, malaria, Japanese encephalitis virus, equine herpes virus type 1, tumor associated) and retroviral (SIV, HIV) antigens [(26, 29, 31-37).] The murine studies collectively show that both protective antibody and CTL activity are induced after rMVA vaccination by various routes: intranasally, subcutaneously, intramuscularly, intravenously; 10^3 to 10^8 infectious units[21] and protection is conferred to both infectious and tumor challenges. While all routes were immunogenic, not all routes were equally protective in all animal model systems. Nuances of immunogenicity variability due to route differences will likely have to be sorted out in humans, as animal model routes do not always convey to humans. Nonetheless, pre-clinical studies provide abundant evidence for MVA as candidate vaccine vector. More details regarding pre-clinical studies are summarized in the investigator’s brochure.

Primate studies

The dynamics of plasma viremia were explored in SIV-infected rhesus macaques (SIVsm E660) that had received prior vaccination with either control MVA or rMVA expressing SIV gag-pol, env (im, 5 x 10^8 CCID50), or rVV expressing SIV gag-pol, env (id, 10^8 pfu/mL) at 0, 12, 20 and 28 weeks. Three of four macaques immunized with rMVA showed low levels of primary plasma viremia with maintenance of normal lymphocyte subsets and intact lymphoid architecture as compared with other groups, which showed high levels of plasma viremia and CD4 T-cell decline. These data link the dynamics and extent of virus replication to disease course and suggest that sustained suppression of virus promotes long-term, asymptomatic survival of SIV-infected macaques[29].

The utility of modified vaccinia virus Ankara (MVA) as a vector for eliciting virus-specific cytotoxic T-lymphocytes (CTL) was explored in the simian immunodeficiency virus (SIV)/rhesus monkey model. After two intramuscular vaccinations (0 and 13 weeks, 10^8 pfu) with recombinant MVA-SIV-SM expressing gag and pol, three of the four Mamu-A*01 monkeys developed a gag epitope-specific CTL response readily detected in peripheral blood lymphocytes after the first vaccination and in all macaques after boosting. The elicited CTL response could be boosted with repeated MVA-SIV vaccinations. This result suggests that immunity to the vector was insufficient to limit viral protein expression on repeated injections. Moreover, those vaccinations also elicited a population of CD8+ T -lymphocytes in the peripheral blood that bound a specific major histocompatibility complex class I / peptide tetramer. These gag epitope-specific CD8+ T lymphocytes also were demonstrated by using both functional and tetramer-binding assays in lymph nodes of the immunized monkeys. These observations suggest that MVA may prove a useful vector for an HIV-1 vaccine and that tetramer staining may be a useful technology for monitoring CTL generation in vaccine trials in nonhuman primates and in humans[31].

More recently, by using a multicytotoxic T-lymphocyte (CTL) epitope gene and a DNA prime-MVA boost vaccination regimen, high levels of CTLs specific for a single simian immunodeficiency virus (SIV) gag-derived epitope were elicited in rhesus macaques. These vaccine-induced CTLs were capable of killing SIV-infected cells in vitro. Flow cytometric analysis using soluble tetrameric major histocompatibility complex-peptide complexes showed that the vaccinated animals had 1 to 5% circulating CD8 (+) lymphocytes specific for the vaccine epitope, frequencies comparable to those in SIV-infected monkeys. Upon intrarectal challenge with pathogenic SIVmac251, no evidence for protection was observed in at least two of the three vaccinated animals. This study does not attempt to define correlates of protective immunity nor design a protective vaccine against immunodeficiency viruses, but it demonstrates clearly that the DNA prime-MVA boost regimen is an effective protocol for induction of CTLs in macaques.

It also shows that powerful tools for studying the role of CTLs in the control of SIV and HIV infections are now available[32].

A multi-epitope gene termed H derived from HIV-1 (Subtype B) and SIV, and containing 20 human (restricted by 12 different HLA molecules), one murine, and three macaque epitopes was constructed. Vaccinations in mice used this H polyepitope expressed from a DNA vector alone or coupled to Plasmodium-derived epitopes (also containing a murine epitope) designed HM. In the polypeptide HM, the H epitopes were coupled at their C-terminus to malaria epitopes that included one murine epitope of *Plasmodium berghei* and eight human epitopes of *Plasmodium falciparum*. BALB/c mice were immunized either IM or IV with 10^6 pfu of MVA. The expression of recombinant polyepitopes from MVA.H and MVA.HM was assessed *in vitro*. Following infection of CEF cells, HM but not H could be detected by Western blot analysis using a specific MAb. No expression of either H or HM polypeptides was detected in COS-1 and 293 human T-cells lines, which are non-permissive for MVA replication, even at a multiplication of infection (m.o.i.) as high as 100. A single vaccination dose of 10^6 pfu administered IM or IV elicited CTL responses against both murine epitopes present in the immunogen, the IV route being more efficient in CTL induction than the IM route. Both MVA.H and MVA.HM induced similar levels of CTL activity against the HIV epitope. The *Plasmodium* epitope in MVA.HM immunized mice elicited consistently lower CTL activities and frequencies of peptide-specific IFN-γ-producing cells than the HIV epitope. A good correlation between CTL activity and the number of cells producing IFN-γ upon peptide stimulation was found for the HIV, but not the *Plasmodium* epitope. Finally, the construction of MVA.HM demonstrated the principle and ease of building complex poly-CTL-epitope vaccines against multiple pathogens[33].

The immunogenicity and protective efficacy of a modified vaccinia virus Ankara (MVA) recombinant expressing the simian immunodeficiency virus (SIV) Gag-Pol proteins (MVA-gag-pol) was explored in rhesus monkeys expressing the major histocompatibility complex (MHC) class I allele, Macaque mulatta (Mamu) A*01.

Macaques received four sequential intramuscular vaccinations with the MVA-gag-pol recombinant virus or nonrecombinant MVA as a control. Gag-specific cytotoxic T-lymphocyte (CTL) responses were detected in all MVA-gag-pol-immunized macaques by both functional assays and flow cytometric analyses of CD8+ T cells that bound a specific MHC complex class I-peptide tetramer, with levels peaking after the second vaccination. Following challenge with uncloned SIVsmE660, all macaques became infected; however, viral load set points were lower in MVA-gag-pol-immunized macaques than in the MVA-immunized control macaques. MVA-gag-pol-immunized macaques exhibited a rapid and substantial anamnestic CTL response specific for the p11C, C-M Gag epitope.

The level at which CTL stabilized after resolution of primary viremia correlated inversely with plasma viral load set point ($p = 0.03$). Most importantly, the magnitude of reduction in viremia in the vaccinees was predicted by the magnitude of the vaccine-elicited CTL response prior to SIV challenge [35].

Prior studies demonstrated that vaccination of macaques with simian immunodeficiency virus (SIV) Gag-Pol and Env recombinants of the attenuated poxvirus modified vaccinia virus Ankara (MVA) provided protection from high levels of viremia and AIDS following challenge with a pathogenic strain of SIV.

This MVA-SIV recombinant expressed relatively low levels of the Gag-Pol portion of the vaccine. To optimize protection, second-generation recombinant MVAs that expressed high levels of either gag-Pol (MVA-gag-pol) or env (MVA-env), alone or in combination (MVA-gag-pol-env), were generated. A cohort of 24 macaques was immunized with recombinant or nonrecombinant MVA (four groups of six animals) and was challenged with 50 times the dose at which 50% of macaques are infected with uncloned pathogenic SIVsmE660. Although all animals became infected post challenge, plasma viremia was significantly reduced in animals that received the MVA-SIV recombinant vaccines as compared with animals that received nonrecombinant MVA ($p = 0.0011$ by repeated-measures analysis of variance). The differences in the degree of virus suppression achieved by the three MVA-SIV vaccines were not significant. Most importantly, the reduction in levels of viremia resulted in a significant increase in median ($p < 0.05$ by Student's t-test) and cumulative ($p = 0.01$ by log rank test) survival. These results suggest that recombinant MVA has considerable potential as a vaccine vector for human AIDS[36].

Neutralizing antibodies were assessed before and after intravenous challenge with pathogenic SIVsmE660 in rhesus macaques that had been immunized with recombinant modified vaccinia virus Ankara expressing one or more simian immunodeficiency virus gene products (MVA-SIV). Animals received either MVA-gag-pol, MVA-env, MVA-gag-pol-env, or non-recombinant MVA.

Although no animals were completely protected from infection with SIV, animals immunized with recombinant MVA-SIV vaccines had lower virus loads and prolonged survival relative to control animals that received nonrecombinant MVA. Titers of neutralizing antibodies measured with the vaccine strain SIVsmH-4 were low in the MVA-env and MVA-gag-pol-env groups of animals and were undetectable in the MVA-gag-pol and nonrecombinant MVA groups of animals on the day of challenge (four weeks after final vaccination). Titers of SIVsmH-4-neutralizing antibodies remained unchanged one week later but increased approximately 100-fold two weeks post-challenge in the MVA-env and

MVA-gag-pol-env groups while the titers remained low or undetectable in the MVA-gag-pol and nonrecombinant MVA groups. This anamnestic neutralizing antibody response was also detected with T-cell-line-adapted stocks of SIVmac251 and SIV/DeltaB670 but not with SIVmac239, as this latter virus resisted neutralization. Most animals in each group had high titers of SIVsmH-4-neutralizing antibodies eight weeks post-challenge. Titers of neutralizing antibodies were low or undetectable until about 12 weeks of infection in all groups of animals and showed little or no evidence of an anamnestic response when measured with SIVsmE660. The results indicate that recombinant MVA is a promising vector to use to prime for an anamnestic neutralizing antibody response following infection with primate lenti viruses that cause AIDS. However, the Env component of the present vaccine needs improvement in order to target a broad spectrum of viral variants, including those that resemble primary isolates[37].

Preclinical studies with the current MVA-HIV env/gag/pol E/A have shown this vector to be immunogenic in mice, eliciting envelope and p24 binding antibodies, proliferative responses, and CTL activity (Earl P, VanCott T et al. unpublished – see Investigator's Brochure). Other rMVA-SIV/SHIV constructs have shown both immunogenicity (cellular and humoral) and boosting with lower viral set points after challenge in our pre-clinical testing (see Investigator's Brochure).

MVA administered in immunosuppressed animals

Neither non-irradiated nor neutron-irradiated (300 rad) rabbits developed any signs of disease after an intravenous injection of MVA (5 x 10^8 pfu). In no case could MVA be recovered from internal organs. The animals showed a delayed but marked antibody response and were protected against a subsequent challenge with the vaccinia virus strain Elstree[38].

MVA was found to be non-virulent for both adult and infant mice after the intracerebral inoculation of high doses. The MVA maintained these properties in immunosuppressed mice. All routes of vaccination were well tolerated in animals including the intradermal route (0.2 mL at 10^6 cell culture infective dose; 50% of cells infected (CCID50)/mL) in rabbits and monkeys[39].

Severe combined immunodeficiency (SCID) mice remained healthy for 133 days of observation when inoculated with MVA at 1000 times the lethal dose of vaccinia virus derived from the licensed Dryvax vaccine seed whereas all mice succumbed to the 3-log lower dose of the Wyeth vaccine seed strain within 30 days[72]. Mice with B cell deficiencies or CD8 T cell deficiencies could be immunized with rMVA and were protected upon vaccinia challenge. However, mice with CD4 T- cell or MHC Class II deficiencies were not protected after rMVA vaccination.

MVA safety has also been studied in immune-suppressed macaques (total body irradiation-TBI, anti thymocyte globulin-ATG, or measles infection). Macaques were inoculated with high doses of MVA (10^9 pfu) via various routes: intradermal (ID), intranasal (IN), and intramuscular (IM). The vaccinations were well tolerated and no clinical, hematological or pathological abnormalities were observed. No MVA was cultivated from their tissues (using CEF culture system) when tested 13 days after inoculation[73].

1.3.2 Human Experience with MVA

A large clinical experience was acquired when non-recombinant MVA was used for primary vaccination against smallpox by ID subcutaneous, and IM routes of over 120,000 humans against smallpox in southern Germany and Turkey. The original idea of using an attenuated vector such as MVA was to sequentially immunize first with MVA and boost with the classical vaccinia vaccine (VV) to offer protection against smallpox with fewer complications. During these extensive field studies, including high-risk patients, no major side effects were associated with the use of the MVA vaccine [40-42].

Four schedules were tested in vaccinia naïve individuals who received a MVA dose at 10^6 CCID50 /mL:

- <u>MVA ID followed by VV scarification</u>: 120,000 subjects were immunized with MVA (0.2 mL, ID) and received the second shot (VV) 14 days later (range five days to six months). The reactions to the first MVA vaccination were a red papule; very few subjects (unspecified in report) had fever >38°C and most of them experienced mild systemic symptoms. The reactions to the second VV vaccination consisted of accelerated mild pustular reaction and no fever. Primary reactions typically observed following VV administration were not observed in this study when VV vaccination was preceded by an MVA priming.

- <u>MVA SC followed by VV scarification</u>: Performed in toddlers (0.2 mL, SC). Palpable nodules four to five days post MVA. Same local reactions post VV as described above.

- <u>MVA IM followed by VV scarification</u>: The lower MVA dose (0.2 mL, IM) was probably too low since all subjects developed primary responses to subsequent VV vaccination. However a higher dose (0.5 mL) was well tolerated with no local or systemic reactions (N=193) and prevented the primary reaction to a second shot with VV as seen in Parts A and B.

- MVA IM followed by MVA IM: Two 0.5 mL MVA IM injections (N = 10 subjects) were given 14 days apart (N = 10). Two of 10 subjects developed a small local infiltrate. No systemic reactions were reported. Four weeks after the second vaccination, all raised their hemaglutination antibodies.

More recently, two British groups continue to test in humans two different rMVA constructs: one expressing malaria genes and the other expressing HIV-1 genes. The malaria-MVA study was recently published showing remarkable safety and tolerability of intradermal vaccination [62] and the induction of immune responses in a prime-boost strategy[63]. The HIV MVA vaccine studies are ongoing in both the UK and Uganda and Kenya (IAVI 003,005,008, and 009). These latter studies are part of a DNA prime MVA boost strategy. Recent work suggests that the rMVA–HIV clade A was well tolerated when delivered intradermally and immunogenic, stimulating HIV-specific T-cell responses in the majority of volunteers[64].

Another group based in Germany is giving a recombinant MVA-HIV (Bavaria Nordic) to HIV infected patients in an effort to enhance their immune responses (i.e. therapeutic vaccination). The volunteer sare tolerating the injections well and enhanced immune responses are developing (personal communication Dr. Thomas Harrar, Erlangen, Germany). This study in seropositives will yield very important results regarding MVA-HIV safety because the volunteers are immunosuppressed, yet appear to tolerate the attenuated vaccine quite well.

1.4. MVA-CMDR Background and Development

MVA-CMDR is a live recombinant Modified Vaccinia Ankara vector vaccine that has been genetically engineered to express Subtype E HIV-1 gp160 (CM235), and HIV-1 gag and pol (protease, non-functional reverse transcriptase, integrase deleted) (CM240) and is grown in chick embryo fibroblasts (CEF) derived from pathogen-free chicken eggs. The harvested cells are pelleted by centrifugation, suspended in culture medium, and disrupted by sonication. Cell debris is largely removed by centrifugation and through a sucrose cushion. The vaccine preparation is supplied in a liquid form at two different concentrations, vials containing either 10^9 or 10^7 pfu/mL in a volume of 0.4 and 1.2 mL, respectively and stored at –80 +/-10 ^{o}C. The vaccines are administered in a volume of 1.0 mL by intramuscular or in 0.1 mL by intradermal route.

1.4.1. Origin of MVA

MVA is originated from the Dermovaccinia strain CVA. The early history of both CVA and MVA is given in Mayr et al. Infection 3:6-14 (1975). In summary from that article, CVA was retained for many years (Ankara Vaccination Station) via donkey-calf-donkey passages. In 1953, Mayr et. al.- purified it and passaged it twice through cattle. In 1954-55, the MVA strain was transferred to the State Institution, Bayerische Landesimpfantalt, and used by the Federal Republic of Germany as a smallpox vaccine. No adverse effects were documented in the 120,000 vaccinated individuals.

In 1958, attenuation experiments with CVA were begun in the laboratory of Dr. Anton Mayr (Institute for Microbiology and Infectious Diseases of Animals, University of Munich, Munich, Germany) by serial passage of terminal dilutions in chicken embryo fibroblasts (CEF). After the 360th passage, the virus was cloned by three successive plaque purifications and maintained in CEF through approximately 570 passages in Dr. Mayr's laboratory. At passage 516, and after its clinical examination in humans, the virus was named "Modified Vaccinia Virus Ankara" or "MVA" Stock to distinguish it from other attenuated vaccinia strains. Dr. Gerd Sutter, working in the laboratory of Dr. Anton Mayr, amplified MVA P572 (2/22/74) to P574 in CEF at which time he plaque purified it by limiting dilution three times in CEF. The first large stock preparation of this further plaque purified virus is P580 8/15/88 (this virus is also referred to as the "F6 clone").

Lyophilized MVA P580 8/15188 was brought by Dr. Gerd Sutter in December 1990 to the laboratory of Dr. Bernard Moss, Laboratory of Viral Diseases, National Institutes of Allergy and Infectious Diseases, National Institutes of Health, Bethesda, and used in the creation of MVA-CMDR.

1.4.2. Origin of HIV GAG/POL and ENV Genes

A plasmid containing the CM235 envelope gene (Subtype E) in the vector pBluescriptSK was received from Dr. Francine McCutchan (Henry M. Jackson Foundation) in 1994. A plasmid containing the CM240 gag/pol gene (Subtype A) in pNOTA was obtained from Dr. Jean Carr (Henry M. Jackson Foundation) in 1997. Both HIV-1 strains, CM235 and CM240, were isolated in 1990 from young man in Northern Thailand randomly selected for national service and who had tested HIV positive. The sequence characteristics for these HIV genes have been published [74-76].

The CM235 envelope gene was altered at nucleotide positions 536-543 and 1156-1162 to eliminate two vaccinia termination translation sites. The strategy used did not alter the correct amino acid coding sequence. Following mutagenesis, the mutations were confirmed by DNA sequencing (position 536-543) or restriction digestion (position 1156-1162). At the latter position, a new restriction exzyme obtained from *E. coli* bacteria (EcoRI) site was generated by the oligonucleotide mutagenesis. The envelope gene was amplified by polymerase chain reaction (PCR) and a thymidine-adenine-adenine (TAA) stop codon was inserted after nucleotide 2217 to create a truncation of 113 amino acids at the C-terminus of gp4l, thus eliminating the cytoplasmic tail of gp 41. The 2.2 kb Smal fragment generated by PCR was ligated into the shuttle vector pLW17 at the Sma siteI. pLW17 contains the modified H5 promoter and directs recombination into deletion II of MVA. *E. coli* strain (DH5α)was transformed with plasmid DNA and individual colonies were picked and were analyzed for the presence and orientation of the insert. The plasmid was named pLW17CM235envt (#19).

The CM240 gag/pol gene was modified by site directed mutagenesis to alter two sites in the reverse transcriptase gene to eliminate reverse transcriptase activity. The CM240 gag/pol gene was amplified by PCR using oligonucleotides that generated a DNA fragment that contains a Hpal site at the 5'end, and a TAA stop codon and a BamHI site at the 3' end of the pol gene.

This cloning strategy deleted the integrase gene, which is located 3' to pol. The gag/pol gene was ligated into the MVA shuttle plasmid pLW9 that contains the modified H5 promoter and directs recombination into MVA deletion III.

DH5α strain STBL2 was transformed and plasmid DNA from individual colonies was analyzed for the presence of the insert. The plasmid was named Plasmid pLW9CM240gag pol.

1.4.3. Production of rMVA Viruses

CEF cells were infected with MVA passage 580 and the cells were transfected with plasmid pLW9CM240 gag/pol. After two days, the cells were lysed and the lysate was used to infect CEF cells. Recombinant virus was identified by immunostaining viral plaques for gag/pol expression and individual positive foci were picked and expanded in CEF cells. CEF cells were then infected with MVA-CM240gag/pol and transfected with plasmid pLW17-CM235envt#19. Virus was isolated from infected cells, and recombinants expressing env were identified by immunostaining plaques with anti-env antibodies. Six successful rounds of plaque purification were performed. The final isolate was named MVA-CMDR (Chang Mai Double Recombinant).

1.4.3.1. Preparation of Bulk Lot, Final Product, and Diluent/Placebo

MVA-CMDR Bulk Lot, Final Product and Diluent were manufactured by the Pilot Bioproduction Facility (PBF) located at the Forest Glen Annex of WRAIR (Walter Reed Army Institute of Research) in Silver Spring, MD. The PBF operates under cGMP and it is fully compliant in all aspects of the GMP regulations as specified in 21CFR210 & 211. MVA-CMDR is manufactured by amplification of the virus in CEF cells derived from certified pathogen-free chicken eggs. The harvested cells are pelleted by centrifugation, suspended in culture medium, and disrupted by sonication. Cell debris is largely removed by centrifugation through a sucrose cushion.

MVA-CMDR is formulated as a sterile liquid in 2 mL vials containing either 10^7 or 10^9 pfu/mL. The MVA-CMDR vaccine contains the following excipients: PBS (without Ca^{2+} and Mg^{2+}), with 7.5% lactose, pH 7.4. The Diluent/placebo consists of PBS (without Ca^{2+} and Mg^{2+}), with 7.5% lactose, pH 7.4, which was also manufactured by the WRAIR PBF under cGMP manufacturing conditions.

TABLE 3. SUMMARY OF PRE-CLINICAL STUDIES MVA-CMDR (VIALED PRODUCT)

Vaccine	Study	Route	Dose/ # Doses/<br>Study Duration	Animals (N)	Study Type/Result
MVA-CMDR<br>(Drug Substance lot # 0957)	In Vitro Tissue Culture Safety (#AA64KV.0030 00.BSV)	Cell Culture Media	Viral Fluids<br><br>2 doses<br>28 Days	Human, Monkey, & Chicken Cells	Safety Study<br><br>No adventitious agents detected
MVA-CMDR<br>(Drug Substance lot 0957)<br><br>Unclarified Viral Fluids	Animal Safety (# AA64KV.0050 42.BSV)	IP 0.5 mL 1 Dose<br>PO 0.05 mL<br>IN 0.05 mL 28 Days<br>IC 0.03 mL		Adult Mice (5M/5F per injection route)	Safety Study<br><br>No adventitious agents detected. All animals remained healthy
		IP 5.0 mL 1 Dose<br>IC 0.1 mL 42 Days		Guinea Pigs (3M/3F per injection route)	
		IP 0.1 mL 1st Dose<br>PO 0.01 mL 14 Days/<br>IC 0.01 mL 2nd Dose/<br>14 Days		Suckling mice (8 per 1st injection/ 6 per 2nd injection)	
MVA-CMDR<br>(drug product lot 0965 & 0966	General Safety-21 CFR 610:11 (AA63YH.509 000.BSV)	IP	5 x 10^7 pfu<br>5 x 10^9 pfu<br><br>1 injection<br>7 days	Guinea Pig<br>(2F)	Safety Study<br><br>No evidence of toxicity-test animals did not demonstrate overt signs of ill health and gained weight over test period
		IP	0.5 x 10^7 pfu<br>0.5 x 10^9 pfu<br><br>1 injection<br>7 days	Mice<br>(4M)	
MVA-CMDR<br>(drug product lot 0965 & 0966	GLP Rabbit Toxicity (#1419-101)	IM<br>ID	10^6, 10^7, & 10^8 pfu<br><br>4 injections<br>78 Days	Rabbit<br><br>(8M & 8F)	Toxicity Study<br><br>No test related changes in any study parameter. Only toxicity observed was injection site reaction with ID injection (10^8 pfu only)
MVA-CMDR<br>(drug product lot 0965 & 0966	Mouse Potency Assay (HIPM20C)	IP	10^8, 10^7, 10^6, & 10^5 pfu<br><br>2 injections<br>(Day 1 and 21)<br>6 Weeks	Mice<br>(10 mice/group with 6 groups)	Immunogenicity Study<br><br>HIV-1 p24, ogp 140, and MVA specific antibodies detected.<br><br>HIV-1 p24 CD8+ T cells detected (intracellular cytokine, tetramer, & ELISPOT)

IP=Intraperitoneal, IM=Intramuscular, ID=Intradermal, IN=Intranasal, IC=Intracranial, PO=Per os (by mouth)

1.4.3.2 In Vitro Tissue Culture Safety (#AA64KV.003000.BSV)

The objective of this test is to determine whether adventitious viral contaminants are present in the MVA-CMDR master seed and concentrated vaccine by injection on three indicator cells (human MRC-5 cells, Monkey Vero cells, and Chicken SL-29 cells). The cells are observed for cytopathic effects (CPE), hemadsorption (HAD), and hemagglutination (HA). Viral Cytopathic effects were not observed in any of the indicator cell cultures inoculated with MVA-CMDR. No evidence of hemadsorption or hemagglutination was observed in the MVA-CMDR inoculated cultures.

Animal Safety (# AA64KV.005042.BSV) The purpose of this assay is to detect viruses that do not cause a discernable effect in cell culture systems. The experimental design is based upon The FDA Center of Biologics Evaluation and Research 1993 guidance document "Points to Consider in the Characterization of Cells Lines Used to Produce Biologicals". Adult mice are included in the assay to detect possible contamination of the vaccine with neurotropic or other viruses such as lymphocytic choriomeningitis virus. Suckling mice are utilized to detect Coxackie or other viruses which would cause the mice to become sick and die. Guinea pigs are sensitive to a variety of viral infections. Adult mice are injected by intraperitoneal, per os (mouth), intranasal, and intracranial routes. Guinea pigs are injected by intraperitoneal and inracrananial routes. Suckling mice are injected by intraperitoneal, per OS, and intracranial routes. The suckling mouse portion of the assay included a sub passage of homogenized tissue after 14 days into a new group of suckling mice followed by an additional 14-day observation period.

All adult mice injected with the MVA-CMDR appeared normal and healthy for the twenty-eight day observation period. Fifteen of the sixteen suckling mice injected with the test article and fifteen of the sixteen suckling mice injected with the negative control article appeared normal and healthy after 14 days. One of the suckling mice in the MVA-CMDR and negative control were missing and presumed cannibalized on Day 6 post-vaccination. The surviving mice of each group were homogenized and the homogenate of each group was passaged into a new group of suckling mice. All suckling mice injected with the homogenate appeared normal and healthy after 14 days. All of the guinea pigs injected with MVA-CMDR remained normal and healthy during the forty-two day test period. Guinea pig rectal temperatures were taken starting on day 21 and remained within normal ranges.

General Safety-21 CFR 610:11 (#AA63YH.509000.BSV)

This study is designed to determine the toxicity of the vialed MVA-CMDR vaccine (lot 0965 & lot 0966) and Diluent (lot 0958) by intraperitoneal injection of mice and guinea pigs. Toxicity is determined by overt signs of ill health, death, or weight loss during the test period. The assay is performed as described in the Code of Federal Regulations, Title 21, Section 610.11. All mice and guinea pigs inoculated with the vaccine or diluent remained normal and healthy throughout the test period. No overt signs of ill health or unusual responses were observed. All guinea pigs weighed less than 400 grams each prior to the injection and gained weight over the seven-day test period. All mice weighed less than 22 grams each prior to injection and gained weight over the seven-day test period.

GLP Rabbit Toxicity (#1419-101)

A repeat dose toxicity study was conducted in New Zealand white rabbits by Therimmune Research Corporation/ Gene Logic, Inc. The rabbits were selected as the test organism based upon recommendations from the FDA Office of Vaccine Research and Review concerning repeat dose toxicity studies for vaccines to support Phase I clinical trials. The intramuscular and intradermal routes of injection and the doses for this study were selected to deliver up to the highest human dose to be used in the Phase I clinical vaccine trial. An additional MVA-CMDR injection was administered to rabbits based upon the FDA guidance concerning the "N+1" rule to support the safety of vaccines for Phase I trials. The study design for vaccination of each rabbit group is shown in the table below. Vaccinations were given to the animals over a nine-week period (study days 0, 22, 43 and 64). Half of the animals were sacrificed and necropsied on Day 65 and the remaining animals were sacrificed on Day 78.

There were N=72 rabbits in total and 36 animals/sex, of approximately 13 weeks of age at study onset. They were randomly divided into six groups:

TABLE 4. Summary of Rabbit Vaccination Protocol

Group	Treatment	Dose (pfu/dose)	Route	Dose Volume (mL)	Males	Females
					N	N
1	MVA-CMDR	0	IM	1.0	6	6
2	Diluent	0	ID	0.1	6	6
3	MVA-CMDR	$1X10^6$			6	6
4	Lot #0966	$1X10^7$	IM	1.0	4	4
5	MVA-CMDR	$1X10^8$			8	8
6	Lot# 0965	$1X10^9$	ID	0.1	8	8

Briefly, this study determined the effects of vaccination on mortality, clinical observations, body weights and changes, food consumption, ophthalmology, organ weights and ratios, clinical pathology and histopathology, clinical chemistries, hematology and coagulation parameters. All three humans doses (10^6, 10^7 and 10^8pfu) were tested. No test article related changes in mortality, cage side observations, clinical observations, body weights, body weight gains, food consummation, ophthalmology, dermal Draize observations, clinical pathology, clinical chemistry, immunology, organ weights, organ to body weight ratios, histopathology, or gross pathology were observed. Significant Draize findings were evident at the intradermal injection site following administration of the 10^8 pfu MVA-CMDR HIV and remained present during the course of the study, following a consistent peak and recovery pattern after each injection. Due to the intradermal injection site Draize findings, it is quite possible that histopathology findings of inflammation at intradermal injection sites are test article related. Additionally, some changes in clinical pathology parameters are possibly test article related but not considered to have any toxicological significance. Therefore, under these study conditions, repeat IM administration of 1 x 10^6, 1 x 10^7, or 1 x 10^8 pfu MVA-CMDR HIV vaccine to New Zealand White Rabbits did not exhibit any signs of local or systemic toxicity with possible histopathological correlate of inflammation, but no obvious signs of systemic toxicity The results of this study are described in detail in the Investigator's Brochure (IB) that is submitted separately for review. Anti-HIV p24 specific antibody titers were detected in serum collect from rabbits.

Immunogenicity of MVA-CMDR in mice (#HIPM20C)

To assess the immunogenicity of the HIV expressed antigens, mice were immunized with MVA-CMDR according to the study design shown in the table below. There were a total of six groups of mice with ten mice per group. Half of the mice (N = 5) in each group received a single intraperitoneal (IP) vaccination (0.2 mL volume in the inguinal region). Three weeks after this single vaccination, these mice were removed from the study and spleens were removed and processed for evaluation of cell-mediated immunity. The other half of the mice in each group (N = 5) received a second IP vaccination (0.2 mL) at Week 3. Three weeks after this second vaccination (Week 6), the mice were removed from the study and spleens were removed and processed for evaluation of cell-mediated immunity.

TABLE 5. Summary of Mouse Vaccination Protocol

Group	Vaccine[1, 2]	Lot #	Number of Mice	Dose (pfu/dose)
1	MVA-CMDR	research	10	$1x10^7$
2	Saline	-	10	0
3	MVA-CMDR	0965	10	$1x10^8$
4	MVA-CMDR	0965	10	$1x10^7$
5	MVA-CMDR	0965	10	$1x10^6$
6	MVA-CMDR	0966	10	$1x10^5$

[1] No adjuvant was used.
[2] IP immunizations were administered at Day 0 and Week 3 in a 0.2 mL volume per dose.

Mice immunized with MVA-CMDR elicit serum IgG responses directed against HIV p24, ogp140, and the MVA vector. Mice administered MVA-CMDR at the three higher doses seroconverted to HIV p24 after the second injection. There was a dose response with groups receiving the higher dose of MVA-CMDR giving the strongest response. Mice immunized with the highest dose (10^8 pfu) seroconverted to p24 after a single vaccination. Mice administered MVA-CMDR at the higher doses also seroconverted to HIV ogp140 after the second injection with the groups receiving the two higher doses (10^8 and 10^7 pfu) seroconverting after a single injection. There again was a dose response with groups receiving the higher dose of MVA- CMDR giving the strongest response. The two higher dose groups seroconverted to MVA proteins after a single vaccination and groups receiving all four doses had detectable MVA-specific responses after a second vaccination. However, these responses were weak in the 10^5 pfu dose group (titer peaked at 200) in contrast to the findings in the rabbit study for the same vaccine lot# (See Section 1.4.3.5).

To evaluate CD8+ responses splenocytes were collected from mice three weeks after the first vaccination for intracellular cytokine staining and ELISPOT. For tetramer staining spelnocytes were collected three weeks after the first and second vaccination. Intercellular cytokine analysis was performed after a 7-day in-vitro stimulation of splenocytes with MVA-CMDR. Intracellular IFN-g production was stimulated by MVA infected cells in each of the groups, with the vaccine group receiving the lowest dose (10^5) having the weakest, but detectable response. HIV gag-specific CD8 T cell responses were detected from groups immunized with the three higher MVA-doses (10^8, 10^7, and 10^6 pfu). MVA-specific CD8 T cell responses were detected at all doses used, though the response at the lowest dose (10^5) was the weakest. Splenocytes were also stained for HIV gag-specific CD8 T cell responses using the H-2K(d)/AMQMLKETI gag tetramer both pre and post MVA-CMDR *in vitro* stimulation (IVS). Pre-IVS gag-specific responses were weak after the first vaccination but were positive (0.4 to 1.6%) after the second vaccination. Responses were enhanced after one week *in-vitro* simulation (IVS) with MVA-CMDR infected P815 cells both after the first and second vaccination. These data demonstrate detectable CD8 T cells or responses specific for the gag epitope from the three highest MVA-CMDR dose groups. HIV p24-specific IFN-γ spot forming cells elicited by MVA-CMDR vaccination were also evaluated by ELISPOT. Splenocytes from the three highest dose groups (10^8, 10^7 and 10^6 pfu) had gag-specific CD8 T cells as detected by this assay. Splenocytes from the lowest dose group (10^5 pfu) had detectable MVA-specific CD8 T cells, though approximately half the number of the three higher dose groups. Overall these three assays support that vaccination of mice with MVA-CMDR generates anti-HIV CD8+ T cell responses.

1.5 Rationale for a Clinical Trial with MVA ENV/GAP/POL Subtype E/A

HIV-1 exists as multiple genetic subtypes (currently designated A-I, O)[43]. In the United States, the vast majority of HIV-1 strains are Subtype B, whereas in Thailand the majority of new infections are with Subtype E[44]. The circulating Subtype E HIV-1 is characterized as a recombinant virus; the env gene derived from Subtype E and the gag/pol genes derived from Subtype A[45-47]. Data from antibody cross-reactivity studies demonstrate that binding and neutralizing antibodies from Subtype B and E infected subjects react preferentially with viruses from the same subtype [48,49]. Conversely, several studies in HIV-infected subjects and also in vaccinees have shown some cross-subtype CTL activities, limited in breadth by the HLA polymorphism of individuals [50,51].

These studies along with the genetic and antigenic variability of HIV-1, the lack of specific knowledge of immune correlates of protection, and the lack of a predictive HIV-1 animal model illustrate the technical limitations of the formal approach to the development of HIV-1 vaccines.

A historically valid approach would be to empirically identify and test products that elicit strong humoral and cellular immune responses to viral strains prevalent within the region where the candidate vaccine will be evaluated. Such an approach is currently being tested in Thailand, i.e. producing and evaluating a recombinant MVA that incorporates genes/proteins isolated from individuals infected with HIV-1 Subtype E/A from Thailand [52-54]. Although, this MVA-HIV construct does not match the HIV-1 Subtype B circulating in North America, it will be tested initially in the US healthy in HIV-uninfected volunteers for safety and immunogenicity.

This is a conservative approach to perform early safety studies in the US before moving products into the international arena. A further rationale for this approach is the extensive deployment of US Armed Forces in South East Asia, and the opportunity of studying cross-subtype CTL in a North American HLA context. If safety and immunogenicity data are compelling, this particular vaccine candidate could then be tested in HIV-uninfected Thai subjects either alone or in prime-boost protocols (DNA prime and MVA boost or MVA prime and subunit boost). A recent clade A MVA-HIV vaccine study suggested that the MVA-HIV vaccine delivered intradermally was immunogenic when given alone and when used as a boost for prior DNA vaccinations, it was better than DNA alone[58]. In this small study, the MVA-HIV clade A vaccine given via the ID route induced HIV specific T-cell responses in the majority of vaccinees. This small data set raises the possibility that rMVA products could be considered as stand alone products. It seems advisable to perform early human studies with single products to help attribute any effects to a single agent. Other possibilities include the use of heterologous vectored products with identical or similar inserts. Of note, there are very few clade E products in development while there remain many clade E endemic areas in the world.

1.6 Rationale for the Vaccination Schedule and Study Groups

Low (10^6 CCID50) and high (10^{7-8} CCID50) doses of other non-replicative live vectors such as canary pox ALVAC-HIV constructs administered by intramuscular injection have been shown to produce both antibody and lymphoproliferative responses in almost all subjects, whereas CTL responses were detected in only 25-35 % of HIV-uninfected volunteers. These cytolytic responses were inconsistently detected using current assay techniques. Intradermal routes are currently being tested in humans. Our experience shows that while the intradermal route induces more local reactogenicity, it is generally mild (erythema, induration and itchiness), well tolerated and does not interefere with the volunteer's daily activities. Furthermore, these local reactions seem to diminish over time and are less visible after the third injection[56]. Long vaccination schedules ranging from six to 12 months have been tested extensively with ALVAC-HIV constructs.

A shorter vaccination schedule is proposed here with three injections over 12 weeks with the expectation to induce some CTL responses, most likely at the higher doses. The hypothesis is that three vaccinations given at over a 12-week interval will induce some relevant HIV immune responses. It is likely that anti-vector responses will also develop, both cellular and humoral. Vaccinees will be tested for the presence of MVA antibody during the study. In order to minimize confounding effects with pre-existing vaccinia immunity, only vaccinia naïve subjects will be recruited for the study.

Efficient and durable CTL responses require endogenous antigen synthesis and processing. HIV-1 internal structural and enzymatic proteins contain conserved domains that preserve their functions. Because these proteins exhibit less antigenic diversity they may elicit effective CTL responses[32]. Vaccine delivery techniques include vaccination with live attenuated viruses, inactivated recombinant virus infection [33], or plasmid DNA expression vectors. A major obstacle in the induction of CTL responses with naked DNA or recombinant virus during development of an HIV vaccine is that the expression of the HIV-1 structural and enzymatic genes is tightly regulated by the virus itself. In recombinant vectors, the expression of these proteins is heavily dependent upon the presence of the Rev-responsive element (RRE) of HIV-1[34,35]. Poor expression is caused by the presence of adenosine and thymidine (AT)-rich inhibitory nucleotide sequences (INS) in the *gag*, *pol* and *env* genes, which inhibit the nuclear export and efficient expression of unspliced HIV-1 messenger RNAs. Early studies of DNA vaccination against HIV in mice required the inclusion of *rev* in their expression vectors[36-39] but modification of INS has been shown to facilitate Rev-independent expression of HIV-1 Gag[28,39] allowing detectable humoral and CTL responses against this protein [39]. These modified HIV-1 *gag* genes produced viral-like particles of the expected density and morphology and induced an immune response to HIV-1 Gag after DNA vaccination in mice[28]. The *nef* region has been incorporated into the plasmid because it has been shown to elicit high levels of $CD8^+$ CTL responses in long-term nonprogressors as well as other HIV-1 infected patients [40,41]. A modified HIV-1 *env* gene has been included that has been shown to produce a gene product that can elicit antibody and CTL responses in mice and guinea pigs against the HIV-1 envelope when administered by DNA vaccination [42].

1.7 Human Experience to Date

Several MVA vaccines (e.g. MVA-malaria, MVA-HIV) are currently in Phase I international trials. Such as a HVTN 055 [A Phase I Trial to Evaluate the Safety and Immunogenicity of rMVA-HIV (rMVA-HIV env/gag+rMVA-HIV tat/rev/nef-RT) and rFPV-HIV (rFPV-HIV env/gag+rFPV-HIV tat/rev/nef-RT) Vaccines, Alone or in Combination, in Healthy, Vaccinia-Naïve HIV-1 Negative Participants] which is assessing the safety and immunogenicity of the simultaneous administration of two modified vaccinia Ankara (MVA)-vectored HIV vaccines as priming doses (one containing *env/gag* and the other containing *tat/rev/nef-RT*), followed by boosting doses of two fowlpox-vectored HIV vaccines containing identical inserts.

RV 158 will be the first study using MVA-CMDR in humans.

The MVA vaccine used in this study is related to the vaccine to prevent smallpox infection (vaccinia virus). In the recent experience with the smallpox vaccine campaign, an uncommon occurrence was myo/pericarditis in vaccine recipients (96 cases/666,712 vaccinations). The RV 158 Team does not anticipate myo/pericarditis complications with the use of this MVA vaccine, a highly attenuated, non-replicating virus. In fact, myo/pericarditis HAS NOT been reported with previous MVA use. However, volunteer safety is of utmost importance; thus, a safe and conservative approach is to monitor for cardiac effects in any related vaccine. Efforts to enhance cardiac monitoring (ECGs and cardiac enzyme testing) have been added to this protocol. These procedures are listed in the study procedure section and explained in the informed consent document (See also IB).

2 PURPOSE OF THE STUDY

2.1 Primary Objective

To evaluate the safety and tolerability of MVA-CMDR (HIV-1 CM235 ENV/ CM240 GAG/POL) administered by IM or ID injection to HIV-uninfected adult volunteers.

2.2 Secondary Objective

To evaluate the ability of MVA-CMDR (HIV-1 CM235 ENV/ CM240 GAG/POL) to induce HIV antigen specific cellular and humoral immune responses.

3 STUDY DESIGN

This is a Phase I study in 48 healthy, HIV-Uninfected volunteers. Volunteers will be screened approximately three months (90 days) prior to the first vaccination. If volunteers meet inclusion criteria, they will be enrolled, and then randomized and blinded (vaccine or placebo) in each group. A consent form will be signed and a test of understanding will be administered to all volunteers prior to enrollment in the study. This study will provide new, preliminary data regarding the safety and immunogenicity of an intramuscular and intradermal MVA-HIV recombinant vaccine candidate.

Two routes of vaccinations will be tested in this study: the traditional IM route, and the ID, which could target resident antigen presenting cells (i.e. interstitial dendritic cells) in the dermis. In the malaria model, intradermal MVA boosting conferred higher protection rates than intramuscular boosting against a pathogenic *Plasmodium* challenge in mice. Three doses of MVA-CMDR will be tested, 10^6, 10^7 and 10^8 pfu. These doses were selected on the basis of previous animal experiments (range 10^7-10^8 pfu) that induced significant immune responses and/or conferred protection against a challenge, and on doses used in humans (10^6 $TCID_{50}$ in the German smallpox, vaccination experience, and 10^7 and 10^8 pfu of rMVA currently tested in Europe, Africa and the United States). Finally, this dosing is compatible with large-scale batch vaccine production. Large-scale production of this rMVA-CMDR-HIV is straightforward and demonstrates high titer and retained insert stability over time. High titer vaccine lots were produced in the 10^{10} pfu/mL range using roller bottle technology. The present study will be one of the first human studies conducted in healthy volunteers in the USA with a live recombinant MVA-HIV construct.

Volunteers will undergo screening (Visit 1, Day-90 to -14), after the informed consent is signed, and then, if eligible, return for follow up visit (Visit 2, Day28 to -3) during which safety labs will be reviewed and repeated. Additionally, a baseline ECG and a blood test for troponin I (cardiac enzyme indicative of cardiac tissue injury) will be performed. Eligible volunteers will be enrolled and then vaccinated a total of 3 times on Days 0, 28, and 84 (weeks 0, 4, and 12). Each vaccination will be followed with a phone call (within 24-48 h) and a safety visit (within 14 days). Two other immunogenicity visits, and follow-up visit for laboratory results only will complete the trial schedule of 11 total visits over a 12-month period.

Volunteers will be observed for a minimum of 30 minutes following injection for acute adverse experiences and will be contacted within 48 hours following the injection for a brief adverse reaction interview. In addition, volunteers will complete diaries for 6 days following each vaccination.

MVA-CMDR will be delivered IM in the deltoid muscle or ID in the volar aspect of the arm at the intervals shown below. For safety reasons, the volunteers will be enrolled into

Parts A and B in a staggered fashion. The Part A. Groups I and II receiving the lower doses (10^6 and 10^7 pfu, respectively) will be enrolled first. The Part B. Groups III and IV, receiving the higher dose (10^7 and 10^8 pfu) will be enrolled after ALL the safety visits are completed after the second injection at the lower dose. All low dose safety data will be reviewed by the Protocol Safety Team: PI, AI, DAIDS MO, and medical monitor. Additional participants could include co-investigators and senior clinical research nursing staff to decide if it is safe to proceed to the higher doses. Only volunteers from the US will be enrolled until completion of the review of low-dose safety data. A third vaccination will be given at 12 weeks.

TABLE 6. Study Schema

Part A	Subjects	Vaccination Schedule		
Group	Vaccine/Placebo[1]	Day of the Study in Part A		
		0	28	84
I	10/2	10^7 pfu IM, 1.0 mL into deltoid muscle		
II	10/2	10^6 pfu ID, 0.1 mL into the volar aspect of the forearm to produce a wheal		
Review of Safety Data for First and Second Vaccinations of Part A Groups				
Part B	Subjects	Vaccination Schedule		
Group	Vaccine/Placebo	Day of the Study in Part B		
		0	28	84
III	10/2	10^8 pfu IM, 1.0 mL into deltoid muscle		
IV	10/2	10^7 pfu ID, 0.1 mL into the volar aspect of the forearm to produce a wheal		

[1]Vaccine: MVA-CMDR ; Placebo: vaccine diluent consists of PBS (without Ca^{2+} and Mg^{2+}), with 7.5% lactose , pH 7.4.

3.1 Study Population

The total study population consists of 48 healthy, HIV-uninfected adults from the United States and Thailand. Subjects will be vaccinia naïve, 18 to 40 years of age at the time of study enrollment. Because of current FDA recommendations for Phase I studies of new vaccines, in this Phase I study, which is designed to establish safety of the vaccine in healthy adults, enrollment will be limited to persons at least 18 years of age, and no older than 40 years of age[1].

[1] Volunteers must be 40 at the time of enrollment. Persons who exceed to the age window during study participation will be allowed to continue with study participation on the study schedule and receive vaccinations.

Within this age range, the majority of volunteers will be naturally vaccinia naïve, the last vaccinia vaccinations having been given in most cases in 1967 (1976 in Thailand), except in the military and other volunteers involved in emergency preparedness plans. The vaccinia status of the volunteers will be confirmed by checking for a scar from prior vaccination, confirmatory laboratory testing, and by medical and vaccination history of each individual.

Volunteers who are healthy, non-military personnel, between the ages of 18 and 40, will be recruited by the principal investigator, associate investigators, and other study team members from the clinical site. IRB approved advertisements will be made available to potential volunteers to inform them of the study, and study participation requirements. The Siriraj site located in Bangkok Noi, Bangkok, Thailand will mainly recruit by announcement of this project to previous clinical trial volunteers (about 150 people), and ask for their help/assistance to invite friends who are potentially interested in this project to contact us either by phone call or meet at the clinic at the Microbiology building. Any questions will be answered by the clinical trial nurses or physician. Some posters will be posted in the Siriraj hospital.

At the AFRIMS facilities located in Wattana, Bangkok and information about the trial such as IRB approved flyers or brochures will be provided in various places such as hospitals, office buildings, or temples. The study team (research assistant, clinical research nurses) will receive the calls and respond to potential subject inquires and set up appointments for prospective volunteers at the clinic.

Volunteers must not be engaging in high-risk behavior for HIV (i.e., injection drug use or sex with HIV positive partner). Both U.S. and Thai military personnel will be excluded from participation in this study due to the potential for a positive HIV test result. Standardized HIV testing is done by the military and could result in adverse personnel action for the participant rendering them no longer eligible for deployment. In addition, due the increase in deployment, most U.S. military personnel recently received the smallpox vaccine, making a large number of personnel not eligible for study participation. Women will agree to practice effective contraception (defined in the informed consent document) for 60 days prior to study entry and for 3 months after the third vaccination. The screening and enrollment period will be approximately 12 weeks for each individual volunteer. Subjects will be followed for six months after the final vaccination (duration of study 12 months).

Volunteers who experience a "breakthrough" HIV-1 infection during the trial will be referred for medical treatment/evaluation and may be referred to an appropriate trial to evaluate the "natural history" of HIV-1 occurring in vaccine recipients, if eligible.

3.2 Inclusion Criteria:

A participant must meet all of the following criteria:

1) Low risk for HIV infection defined as in Attachment VI
2) 18 to 40 years at the time of enrollment and vaccinia naive
3) Good health (determined by medical history, physical examination, and clinical judgment)
4) Availability for 12 months of participation.
5) Successful completion of the Test of Understanding (maximum of three attempts to pass the test).
6) Literate individuals able and willing to give informed consent.

Laboratory Criteria within 45 days prior to enrollment:

7) Hematocrit: Women: 35 % to 45 %; Men 36 % to 49 %
8) White cell count: 3,000 to 11,000 cells/mm^3
9) Platelets: 125,000 to 450,000 per mm^3
10) Normal cardiac enzyme level at second Screening Visit (Day –28 to -3) (troponin I level within the institutional upper limit of normal)
11) Urinalysis (UA) for protein and blood: negative or trace. If either is $\geq$1+, repeat the UA. If evidence of blood or protein on repeat UA (repeat microscopic) is confirmed, the volunteer is ineligible and will be appropriately referred for diagnosis and treatment.
12) Normal liver function tests to include ALT/AST, alkaline phosphatase, GGT ($\leqq$1.25x institutional upper limits of normal), CPK ($\leq$480 ng/mL), and creatinine ($\leqq$1.25x institutional upper limits of normal)
13) Negative serology for HIV infection (ELISA test, WB), and HIV RNA below detection limits of FDA approved viral load diagnostic assay.

Female-Specific Criteria:

14) Any female volunteer must have a negative pregnancy[2] test at the screening visit as well as immediately prior to each vaccine/placebo vaccination, as well as verbal assurance that adequate birth control measures have been followed for 60 days prior to the first vaccine/placebo vaccination and will continue to be followed for at least 3 months after the final vaccine/placebo vaccination. This means using any of the following methods:
    - Birth control drugs that prevent pregnancy given by pills, shots or placed under the skin
    - Male or female condoms with or without a cream or gel that kills sperm
    - Diaphragm or cervical cap with a cream or gel that kills sperm
    - Abstinence

[2] Urine pregnancy tests only will be performed at the participating Thai site.

3.3 Exclusion Criteria:

A volunteer will be excluded if one or more of the following conditions apply.

A woman who:
1) Is pregnant.
2) Is breast-feeding.

Anyone who:

1. Is U.S. or Thai military personnel.
2. Acknowledges engaging in highest-risk behavior within six months of study entry: (e.g., active injecting drug use or sexual intercourse with a known HIV-1 infected partner).
3. Has active tuberculosis or other systemic infectious process by review of systems and physical examination.
4. Has history of or known cardiac disease including any of the following:
    - prior myocardial infarction (heart attack)
    - angina pectoris
    - congestive heart failure
    - conduction disturbances
    - repolarization (ST segment or T wave) abnormalities
    - serious cardiac arrhythmias (ventricular tachycardia or ventricular fibrillation)
    - cardiomyopathy
    - pericarditis, stroke or transient ischemic attack
    - chest pain or shortness of breath with activity (e.g. climbing stairs)
    - valvular heart disease including mitral valve prolapse
    - other heart conditions under the care of a doctor

5. Has ECG on Screening Visit 2 (Day -28) with clinical significant findings, or features that would interfere with the assessment of myo/pericarditis (as determined by the contract ECG Lab) including any of the following:
    - conduction disturbance (atrioventricular or intraventricular condition, left or right bundle branch block, AB block of any degree or QTc prolongation)
    - repolarization (ST segment or T wave) abnormality
    - significant atrial or ventricular arrhythmia
    - frequent atrial or ventricular ectopy (e.g. frequent premature atrial contractions, 2 premature ventricular contractions in a row)
    - ST elevation consistent with ischemia
    - Evidence of past or evolving myocardial infarction

6. Has history of seizure disorder, immunodeficiency, chronic illness, autoimmune disease, diabetes mellitus active malignancy or use of immunosuppressive medications.
7. Has evidence of psychiatric, medical and/or substance abuse problems during the past six months that the investigator believes would adversely affect the volunteer's ability to participate in the trial.
8. Has occupational or other responsibilities that would prevent completion of participation in the study.
9. Has received any live attenuated vaccine within 60 days of study entry.
10. Has used experimental therapeutic agents within 30 days of study entry.
11. Has received blood products or immunoglobulins in the past three months.
12. Has history of anaphylaxis or other serious adverse reactions to vaccines.
13. Has previously received an HIV vaccine or an MVA or vaccinia vaccine.
14. Has chronic or active Hepatitis B or Hepatitis C virus infection or active syphilis (positive RPR and FTA).
15. Has had an immediate type hypersensitivity reaction to eggs, egg products or neomycin/streptomycin (used to prepare MVA vaccine).
16. Is a study site employee.

3.4 Concomitant Medications

Concomitant medications are recorded for 90 days prior to first vaccination and at every study visit. The restrictions for concomitant medications stated by the inclusion/exclusion criteria for the first vaccination are applicable for the second and third vaccinations. If an enrolled subject:

- Develops the need for a medication that is prohibited by the eligibility criteria, then further study vaccinations will be discontinued.

- Receives at least one study vaccination, clinical and laboratory evaluations specified by the study through the 52 weeks of the study should be completed, if there is no change in safety considerations.

3.5 Precautionary Medications and Procedures

Medically indicated vaccines (e.g., influenza, tetanus, Hepatitis A, Hepatitis B or rabies) are not exclusionary but should, if possible, be given at least two weeks before or two weeks after MVA-CMDR/placebo vaccination to avoid potential confusion of adverse reactions. However, if rabies or tetanus vaccine is indicated in the post-exposure setting, it must take priority over the study vaccine.

In the event that a rabies vaccination series is medically indicated, the volunteer will not undergo further vaccinations with the vaccine/placebo or this study.

Volunteers participating in RV 158 are discouraged from donating blood during the time of the study due to the potential confusion with a positive HIV screening test (a false positive) at blood banks. Furthermore, blood banks will exclude RV 158 volunteers directly from donating blood upon disclosure of their participation in a live viral vectored vaccine trial.

3.6 Schedule of Clinical Procedures and Laboratory Assays

Evaluation of the safety of this vaccine will include laboratory studies, physical assessment by clinical staff and subject reports on signs and symptoms following vaccinations. Potential adverse reactions will be further evaluated prior to continuing the vaccination schedule. Listed below are the tests to be performed during this study. The schedule for these tests is attached in Attachment IV. Total blood volume drawn from each subject will not exceed the NIH Clinical Center Guidelines of 450 mL in any six-week period.

Laboratory assays for screening and safety will be performed at the AFRIMS Clinical Laboratory at Bumrungrad hospital or at the main research building of AFRIMS depending upon the assay. Immunogenicity studies requiring fresh specimens will be done at AFRIMS. Immunogenicity assays utilizing frozen specimens will be shipped to USMHRP in Rockville.

All study specimens will be archived at the AFRIMS Specimen processing lab at Bumrungrad hospital, co-located with AFRIMS Clinical Trial Center.

3.6.1 Screening

A qualified study team member such as the PI, AI, or Research Nurse will inform each volunteer of the study, provide him/her with any study details and informed consent to aid him/her in his/her choice for voluntary participation. After all information has been provided the subject will be given the necessary time to make an informed decision as to whether or not to participate. Study Volunteers will receive a briefing from the PI Principal Investigator (PI) or designee during which the study will be explained and participation requirements outlined. The content of the briefing is provided as shown in Attachment I.

The briefing will be followed by an opportunity for questions from the volunteers. Research Nurses will then review the consent form with potential volunteers and answer any questions. After review with the study nurse, an Informed Consent Form (Attachment I) will be signed, and Test of Understanding (Attachment III) will be completed by all volunteers, prior to enrollment in the study.

The test of understanding is reviewed "one-on-one" with the volunteer and a member of the study team.

To determine eligibility, volunteers who have given written informed consent and have passed the test of understanding, will undergo a complete medical history and physical examination and screening laboratory assessments as defined in Section 3.2, "Inclusion Criteria." During this evaluation, additional questions or concerns will be elicited from the volunteer.

Initial screening for this study will include:
- Medical history
- Physical exam
- CBC with differential
- Serum AST/ALT, CPK, GGT, alkaline phosphatase, and creatinine.
- RPR
- FDA approved HIV ELISA/Western Blot
- HIV RNA PCR with Roche 1.5
- Hepatitis serology
- Pregnancy test for all females[3]
- Urinalysis, if urine protein or blood is $\geq$1+, repeat the UA. If evidence of blood or proteinuria on repeat UA (repeat microscopic) is confirmed, the subject is ineligible and will be appropriately referred for diagnosis and treatment.
- Questions regarding sexual behavior and other practices.
- Risk status for HIV infection will be determined by a series of questions designed to identify risk factors.
- Samples collected for storage of peripheral blood mononuclear cells (PBMCs), plasma and serum[4]
- General eligibility for clinical trials will be dependent on results of laboratory tests and answers to the interview questions.
- Informed consent documents for vaccine trials will be reviewed, a test of understanding completed.

Counseling related to the potential risks of becoming pregnant during this trial and avoiding HIV infection will be provided. Pre-HIV test counseling and post-HIV test counseling will be provided to all volunteers (and throughout the study as indicated). Study volunteers who qualify for inclusion based on the history and physical, and lab results will be scheduled a second Screening Visit. This approach will allow study staff to evaluate the reliability of volunteers, increase coordination for the vaccine visits, re-enforce the need for compliance on the designated vaccination days and review eligibility test results in person.

[3] Urine pregnancy tests only will be performed at the participating Thai site.

[4] All study specimens will be archived at the AFRIMS Specimen processing lab at Bumrungrad hospital, co-located with AFRIMS Clinical Trial Center. Subject case report forms and source documents for Thai participants will be stored onsite in a secure location with limited access by study staff.

At this second Screening Visit, volunteers will have an ECG and blood samples taken for cardiac troponin I, CBC with differential, chemistries and a urinalysis. The ECGs will be read by a contract ECG lab to evaluate for clinically significant findings or features that would interfere with the assessment for myo/pericarditis. Eligible

and willing volunteers will be scheduled for an appointment for enrollment and the initial vaccination visit within four weeks.

3.6.2 Day 0 Through Day 252 Follow-Up

Day 0 is defined as the day of the first vaccination and the day of study enrollment. The protocol specific eligibility is reviewed on Day 0 and the evaluations used for screening must have been completed no more than 45 days prior to the Day 0, Visit 3.

However, if clinical assessment on Day 0 suggests significant changes may have occurred since the Screening Visit, then the physical examination, hematology tests, blood chemistries and urinalysis done on Day 0 must be used for eligibility. Laboratory results will be available within approximately two days, results are sent by fax directly to the clinic.

Awaiting review of the repeat tests could delay the vaccination for 3 days or more depending on the timing and health status of the volunteer. These labs are flagged by the clinic research coordinators and tracked closely for availability. Pregnancy test results for women of reproductive potential must be obtained on each vaccination day prior to the vaccinations. Day 0 evaluations prior to the first vaccination are the baseline for subsequent safety assessments. Refer to the itemized list in this section and the table in Attachment IV for details on when each type of evaluation must be completed.

The schedule of study agent administration is Day 0, 28 ± 7 days and 84 ± 14 days (with at least 21 and 42 days respectively between vaccination days):

- Following study vaccinations, subjects will be observed for a minimum of 30 minutes.
- Vital signs (temperature, blood pressure, pulse and respiratory rate) will be taken at 30-45 minutes post-vaccination.

- The injection site will also be examined. If erythema or induration are present, the diameters will be measured and the largest diameter recorded.
- Volunteers will be instructed to maintain a diary of local and systemic reactions for seven days post-vaccination. They will be provided with a ruler to measure erythema, induration or other observable reactions.

In addition, volunteers will be provided with a thermometer to assess temperature on a daily basis. The study team will review the procedures with volunteers to ensure accuracy and completion of the diary card and will be given emergency contact information prior to volunteer departure from clinic.

- Follow-up on subject well-being will be assessed by a phone call within 48 hours following each vaccination.
- A formal Safety Visit will occur 14 days ±5 days after each vaccination. Long-term follow-up visits are at Day 168 ±14 days and Day 252 ±14 days.
- Volunteers will be encouraged to report any adverse experiences at any time and may be asked to come into the clinic for evaluation by the research medical team.
- All adverse experiences will be recorded, and the relationship to the test product will be assigned by the PI and reviewed by the Local Medical Monitor. All adverse experiences will be tabulated by relationship to study product, organ system, severity and temporal relation to vaccination.

At intervals throughout the study subjects will also have blood drawn for immunologic assays. Any cells, serum or plasma not used will be stored for future virological and immunological assays. Additional study visits may be required if in the PI's opinion, any lab value warrants repeating OR if the specimen was lost or not run by the contract lab for any reason OR if the clinic did not obtain the specimen for any reason.

3.6.3 Day –90 to -14

- Obtain informed consent
- Assign PIN and screening number
- Obtain and record medical history, including allergy history, with particular emphasis given to inclusion and exclusion criteria
- Perform physical exam including weight, height and vital signs
- Provide pre-HIV test counseling
- Perform Extended risk assessment
- Provide Risk behavior counseling
- Collect samples for HIV diagnostics
- Collect sample for CBC with differential
- Collect blood for EBV transformation of B cells and archival plasma, serum and cells specimens
- Collect sample for RPR, HBsAg, Hep C and sample for chemistries to include: serum ALT/AST, alkaline phosphatase, GGT, CPK, and creatinine
- Collect samples for archiving
- Perform pregnancy test (qualitative HCG assay) for female volunteers

- Urinalysis, if urine protein or blood is $\geq$1+, repeat the UA. If evidence of blood or proteinuria on repeat UA (repeat microscopic) is confirmed, the Subject is ineligible and will be appropriately referred for diagnosis and treatment.
- Successful completion of the Test of Understanding
- Give subject a copy of vaccine informed consent to review at home
- Complete the Volunteer Registry Data Base Sheet

3.6.4 Day –28 to –3

- Perform interim history and physical and abbreviated risk assessment
- ECG
- Provide post-HIV test counseling for test results from Day -90.
- Safety lab panel including CBC with differential, ALT/AST, alkaline phosphatase, GGT, CPK, creatinine and troponin I.
- Collect blood for PBMC and serum archives
- Urinalysis, if urine protein or blood is $\geq$1+, repeat the UA. If evidence of blood or proteinuria on repeat UA (repeat microscopic) is confirmed, the subject is ineligible and will be appropriately referred for diagnosis and treatment.

3.6.5 Vaccinations (Days 0, 28, and 84)

Day 0: Verify eligibility and informed consent documentation ("enroll" volunteer into study). Vaccinations will be administered by a qualified study team member such as the Principal or Associate Investigator or Research Nurse.

Days 0, 28, and 84 (+/- 14 days):

- Obtain interim H&P, including vital signs, and abbreviated risk assessment.
- Provide Risk behavior counseling
- Perform pregnancy test for women immediately before vaccination[5]. **RESULTS MUST BE KNOWN PRIOR TO VACCINATION.**
- Collect samples for immunogenicity/archive: binding and neutralizing antibodies, LPA, CTL, ELISPOT IFN-γ, ICS, CBC, and Chemistries (ONLY at Day 0).
- Urinalysis (Day 0)

[5] Urine pregnancy tests only will be performed at the participating Thai site.

- Administer vaccination in the deltoid muscle as deep intramuscular (IM, 1 mL) or as intradermal injection (ID, 0.1 mL) to the volar aspect of the forearm to produce a wheal.
- Examine the injection sites for local reactions at 30 minutes post-vaccination and instruct subject in the evaluation of these local reactions; measure pulse, blood pressure, and respirations, observe and instruct while the subject takes temperature (as a guide for subsequent evaluations); enter findings on appropriate source documents and CRF.
- Subjects will record daily symptoms for seven days post-vaccination (including the day of vaccination), and will be instructed to contact the investigator if any untoward effects occur.

3.6.6 Twenty-four to Forty-eight Hour Contact Post Each Vaccination:

Subjects will be contacted by staff within 24 to 48 hours post-vaccination (by telephone or other prearranged mechanism) to determine whether adverse effects have occurred. Subjects will be queried using a predetermined review of systems/reactogenicity worksheet. If the adverse effects experienced by the volunteer are deemed "moderate", he/she will be asked to return to clinic for evaluation by the investigator. Additional contacts will be arranged as indicated.

3.6.7 Safety Evaluations

Days 14, 42, and 98 (+/- 5 days):

- Collect and review diary, obtain interim history and physical.
- ECG
- Provide pre-HIV test counseling for today's test (ONLY on Days 42 and 98).
- Draw HIV serology/diagnostics (ONLY on Days 42 and 98)
- Provide post-HIV test counseling for test results from Day 42 (ONLY on Day 98).
- Collect sample for CBC with differential
- Collect sample for serum ALT/AST, alkaline phosphatase, GGT, CPK, creatinine and troponin I.
- Urinalysis
- Collect samples for immunogenicity: binding and neutralizing antibodies, LPA, CTL, ELISPOT IFN-γ, ICS (ONLY on Day 42 an 98).
- Collect archival blood specimen

3.6.8 Day 168 (+/-14 days):

- Perform history and interim physical exam.
- Perform abbreviated risk assessment and provide post-HIV test counseling for test results from Day 98.
- Collect samples for immunogenicity/archive: binding and neutralizing antibodies, LPA, CTL, ELISPOT IFN-γ, ICS

- Collect archival blood specimen
- Archive cells, plasma, and serum

3.6.9 Study Termination Visit, Day 252 (+/- 14 days)

Final Visit for clinical and immunological follow-up:

- Obtain complete history and physical exam including vital signs.
- Review overall study plan and progress with volunteer.
- Perform extended risk assessment and provide risk behavior counseling.
- Provide pre-HIV test counseling*.
- Collect HIV serology/diagnostics, and serum ALT/AST, alkaline phosphatase, GGT, CPK, CBC with differential, and creatinine.
- Urinalysis
- Collect samples for immunogenicity: binding and neutralizing antibodies, LPA, CTL, ELISPOT IFN-γ, ICS
- Collect archival blood specimen

*results with post-test-counseling will be provided to the volunteer

3.7 Laboratory Result Follow-Up Visit 11, Day 280 (+/- 7 days)

Participants will be asked to return to the clinical for a final review of the HIV and laboratory tests completed during the study termination visit. No assessments will be conducted or further blood samples drawn. If a volunteer has a positive HIV result or abnormal laboratory results they will be referred to their primary physician for treatment.

4 STUDY MANAGEMENT

4.1 Monitoring Post-Vaccination Experiences that are Possible Reactions to Vaccine/Placebo Vaccination

Selected local and systemic adverse experiences are routinely monitored in vaccine clinical trials as indicators of vaccine reactogenicity. It is recognized that each of these experiences, and particularly those of a systemic nature, may under some circumstances, in any individual subject, have a cause that is unrelated to

study vaccines. However, as a matter of convenience and in accordance with common clinical practice, all such experiences occurring within seven days of vaccination (including the day of vaccination) are herein termed "post-vaccination reactions."

4.1.1 Post-vaccination Reactions Occurring Immediately After Vaccination

Following each vaccine administration, volunteers will be observed in the clinic for minimum of 30 minutes. Qualified study personnel will then evaluate the volunteer for any signs or symptoms of local or systemic reactions. These will be noted in the case report form. Possible local reactions that should be recorded include erythema and induration (measured using the ruler indicated on the diary card that will subsequently be provided to the subject), and pain/tenderness, or warmth at the injection site. Specific symptoms to be noted include fever (>38°C), fatigue, headache, myalgia, arthralgia, chills, nausea/vomiting, shortness of breath, chest pains, and rashes. For each vaccination, oral temperature will be recorded prior to vaccination and for a minimum of 30 minutes after vaccination. In addition, the pulse, blood pressure, and respirations will be recorded.

4.1.2 Local Reactions Within Seven Days Post-vaccination (Study Days 0 to 6)

The subject will be asked to note occurrences of erythema and induration (measured using the ruler included on the diary card), and pain/tenderness, itching, swelling, or warmth at the injection sites daily for seven days including the day of vaccination. These occurrences should be recorded on the diary card provided to serve as a reminder to the subject for the next clinic visit. The original diary card will be kept by the study site.

4.1.3 Systemic Reactions Within 7 Days Post-vaccination

The subject will also be asked to record the following occurrences for seven days on the diary card:

- fever (>38°C)
- fatigue
- headache
- chest pains
- heart palpitations
- dizziness
- shortness of breath
- myalgia
- arthralgia
- chills
- nausea/vomiting
- rashes
- loss of appetite
- itching (general and local)
- diarrhea

- Additionally, volunteers should record oral temperature six hours post vaccination, and then daily for the next six days.

4.1.4 Post-vaccination Contact

Between 24 and 48 hours after each vaccination, the subject will be contacted by the investigator or study nurse to obtain local and systemic reaction data and to assess clinical status.

4.1.5 Instructions to Volunteers Regarding Unusual or Severe Signs or Symptoms

Volunteers will be instructed to call the specified study personnel immediately if any unusual or severe sign or symptom appears after vaccination. Volunteers with unusual, moderate, or severe sign and/or symptoms post vaccination will be asked to return to the clinic for evaluation. They will be followed up clinically until resolution of symptoms. Contacts will be arranged as needed. If a subject is unable to return to the clinic for evaluation they will be instructed to contact emergency services, with contact information located on the study emergency card provided.

If a subject develops symptoms or findings consistent with the new onset of myo/pericarditis (such as chest pain, dyspnea, palpitations, congestive heart failure or ECG abnormalities or an elevated troponin I) an ECG, echocardiography, cardiac enzymes (both CPK-MB and troponin I), ESR or CRP, cardiology consultation, and follow-up for a minimum of one year will be done.

4.2 Monitoring for HIV Infection

Diagnostic HIV testing will be carried out using an algorithm as described below. Information to the clinical staff of each vaccine trial site will not include the results of specific tests, but will rather state only ”positive" or "negative". This system will allow timely HIV testing without compromising the double-blind nature of the trial.

An HIV-1 ELISA assay, WB, and Roche Amplicor version 1.5 (all tests FDA-approved) will be performed four times throughout the course of the protocol (days -90, 42, 98, and 252).

If Amplicor is positive:

a) If the patient is suspected of being HIV infected and will be called back within 2 weeks. A verification blood specimen will be obtained for HIV RNA PCR.
b) If Amplicor test indicates that the sample is below the limit of detection, then the person is considered HIV uninfected.

c) If the repeat test is positive by the Amplicor assay, a diagnosis of HIV infection is established.
d) If diagnosed as infected with HIV, the volunteer will receive a referral for medical evaluation in their post-test counseling.

4.3 Management of Volunteers Who Become HIV-Infected During the Study

Volunteers who become HIV-infected during the trial:

- will be excluded from further vaccinations
- will be referred for medical treatment. The staff of the designated treatment clinic will be provided with the test results upon which the diagnosis of HIV infection is based.
- if eligible, will be offered enrollment in a protocol studying the natural history of HIV infection in volunteers who become infected on study or after study completion.

Management of HIV-infected Individuals (both Thai sites):
Those volunteers who are determined to be HIV-infected will be counseled and referred for evaluation on the clinical status of their infection, and receive standard treatment and care based on the most recent "National Guidelines for Clinical Management of HIV/AIDS in Adults and Children" published by the Thai Ministry of Public Health. Treatment with potent, combination antiretroviral drugs is widely available in Thailand. Infected individuals will receive private one-on-one counseling regarding HIV infection, how to protect themselves and how to protect their partners. They will be encouraged to notify their partner(s) of the positive test results and have their partner(s) come in for counseling and testing. Those persons, who are not eligible to enroll because of HIV infection, will be counseled on social harm reduction methods, so that their friends and family do not become aware of their HIV status based on their subsequent non-participation in the trial.

Management of laboratory test results other than HIV (both Thai sites):
Volunteer will be informed any abnormal test results, and all tests upon request. The physician-investigator will treat any abnormality as appropriate or refer the volunteer for further evaluation and care. A copy of their test results will be provided to the volunteer upon their request.

All vaccine recipients will also be offered diagnostic HIV testing and counseling upon request after completion of the study but will remain blinded until study completion.

4.4 Criteria for Withdrawal of Volunteers from Vaccination Schedule

Under certain circumstances, a subject will be terminated from participating in further vaccinations. These specific experiences include:

- Investigator's clinical judgment is that it is in the best interest of the subject
- Confirmed HIV infection.
- Pregnancy.
- Type 1 hypersensitivity associated with vaccination.
- Serious intercurrent illness that is not expected to resolve prior to the next scheduled vaccination.
- Treatment with systemic glucocorticoids (e.g. prednisone) or other immunomodulators other than NSAIDs for any reason.
- Need for concomitant vaccine that requires discontinuation.
- Repeated failure to comply with protocol requirements.
- The study sponsor or the Principal Investigator decides to stop or cancel the study.
- WRAIR HURC and HSRRB or the FDA request that the study be stopped. Thai IRBs may request that the study be stopped at the Thai site.
- Volunteer's request.

Additionally, any subject who experiences a clinical and/or biological vaccine related Grade ≥ 3 adverse experience will not receive further study vaccinations until his/her toxicity findings are discussed by the Protocol Safety Team. This committee will decide, depending on the nature of the adverse experience, if continuing with study vaccinations is advisable or if study termination is granted.

Study vaccinations will not be given until values return to normal.

Wherever possible, the tests and evaluations listed for the termination visit should be carried out if the subject refuses follow-up according to the protocol visit schedule. The sponsor should be notified of all study withdrawals within 24 hours. Volunteers who withdraw from the study will be replaced only as long as the study is still open for enrollment. No replacement will be allowed when the enrollment is closed.

Participants who miss an vaccination by more than 15 days will have no further vaccinations performed but will continue to be followed.

Participants who are discontinued from additional study vaccinations will continue to be followed according to the schedule to further evaluate safety and monitor adverse experiences.

4.5 Obtaining Replacement Vial Assignment

If a subject withdraws from the study, the next available volunteer will be placed in the vacated slot. Replacement will only be permitted during the active enrollment Phase.

All volunteers who receive at least one vaccination will be included in the safety analysis. Only volunteers who have received three vaccinations and who have had the visit for blood draws after two weeks post-third vaccination will be included in the final immunogenicity analysis. However, immunogenicity data may be used in the analysis of earlier time points.

Additionally, if the subject:

- Misses a vaccination by more than 15 days, no further vaccinations will be performed, but the subject will continue to be followed according to the protocol visit schedule.
- Misses a non-vaccination visit, vaccination will continue as planned.
- Does not complete the vaccination schedule secondary to a serious adverse experience or toxicity, he or she will continue to be followed according to the protocol visit schedule, and, at a minimum, until the adverse experience/toxicity is resolved and/or the cause is identified.
- Becomes pregnant before completing the vaccine series; then, the subject will have no further vaccinations regardless of outcome. However, she will be followed for all remaining scheduled visits according to the schedule evaluations. A Pregnancy CRF will be completed. The site will maintain contact with pregnant volunteers to obtain pregnancy outcome information for the Pregnancy Follow-up CRF.

NOTE:

A genuine effort will be made to determine the reason(s) why a subject fails to return for the necessary visits. If the subject is unreachable by telephone, a registered letter, at the minimum, will be sent to the subject requesting contact with the clinic. This information will be recorded on the appropriate source document.

5 PHARMACOVIGILENCE, SAFETY AND ADVERSE EXPERIENCE REPORTING

5.1 Pharmacovigilence and Criteria for Stopping Study

The PI, co-investigators and clinical research team will exercise due diligence in ascertaining, accurately recording and promptly reporting as needed all reportable adverse experiences (AE) including serious adverse experiences (SAE) during the study. Case report forms (CRFs) will be completed by the research staff on a daily basis as the data become available from the clinic or laboratory. All recorded AE and lab data will be coded for severity using the standard toxicity grading scale included in Attachment V.

The Principal Investigator and/or co-investigators will monitor and analyze study data including all AE and lab data on a daily basis and will make determinations regarding the severity of adverse experiences and their relation to study product. The entire clinic team will be trained in recognition of AEs, use of toxicity grading scales and the list of reportable AEs (SAEs and AEs which trigger study pause rules). The clinic team will be assisted in this responsibility by the Protocol Safety Team which will review AE/SAEs on a regular basis and ad hoc as needed. This team includes the following: PI, AI, DAIDS MO, and medical monitor. Additional participants could include co-investigators and senior clinical research nursing staff.

To insure that all reportable AEs are captured in a timely manner, CRFs will be entered daily by two separate operators (double key entry), reconciled and subjected to daily analysis to identify reportable AE and the advent of constellations of AE which may invoke study pause rules.

Automated email messages will be issued as an output from this analysis that will notify the members of the Protocol Safety Team of the reportable AE(s). A report form will be completed by the PI or designated associate investigator for reportable AE/SAE and submitted via fax to the USAMMDA Regulatory Affairs Office of Research Protections (ORP) (RA) and the USMHRP Regulatory Operations Center (ROC) for distribution to the safety team. A face-to-face meeting with teleconference (when needed) between responsible parties will be arranged to assess and manage the advent of potential study pause or termination.

In addition, the Protocol Safety Team will review aggregate safety data reports in a weekly meeting that will include participating investigators from Thailand Safety data reports and changes in study status are submitted to the IRB. The sponsor is responsible for reporting to the FDA. All Grade ≥3 AE with the exception of Grade 3 subjective reactogenicity symptoms should be reported immediately and all others within 24 hours. Initial contact should be made by fax contact to the USAMMDA Regulatory Affairs (RA) with email follow-up to the entire Protocol Safety Team.

TABLE 7. STUDY PAUSE RULES

	Symptom or Adverse Experience (AE)	Toxicity Grade	Criteria	Actions Required[1, 2]
1	Any local or systemic symptom, lab toxicity or adverse experience	Grade 4	One vaccine-related symptom at specified grade	Immediate notification of Protocol Safety Team, FDA, USMHRP-ROC, MRMC HSRRB and USAMMDA-RA<br>All study vaccinations are held until further notice<br>Follow-up and resolution<br>SAE report
2	Lab toxicities, fever, vomiting, or other clinical adverse experiences (exception: subjective local or systemic symptoms as listed below in item 5)	Grade 3		
3	Lab toxicities, fever, vomiting, or other clinical adverse experiences (exception: subjective local or systemic symptoms as listed below in item 5)	Grade 2	Two or more participants experience the same vaccine-related symptom at specified grade or higher	Prompt notification of Protocol Safety Team, USMHRP-ROC, MRMC HSRRB and USAMMDA-RA<br>All study vaccinations are held until further notice<br>Follow-up and resolution
4	Erythema or induration	Grade 2 or 3	One or more of the same vaccine-related symptom at specified grade	
5	Subjective local or systemic symptoms, which include pain, tenderness, malaise and/or fatigue, headache, chills, nausea, myalgia, arthralgia.	Grade 3	Two or more participants report the same vaccine-related symptoms at specified grade or higher	Prompt notification of PI via email or phone, Protocol Safety Team, USMHRP-ROC, MRMC HSRRB and USAMMDA-RA<br>All study vaccinations are held until further notice<br>Follow-up and resolution

[1]Site is responsible for submitting to their IRB (per local IRB requirements) IND Safety Reports, SAE reports, and notifications of vaccination holds due to safety pause rules.
[2] Actions Required are defined as follows:

Immediate Notification:
Site notifies Protocol Safety Team, USMHRP-ROC, MRMC HSRRB and USAMMDA-RA immediately. All study vaccinations are held until further notice. USAMMDA notifies FDA within 1 working day of receiving notice of event.

Prompt Notification:
Site notifies Protocol Safety Team, USMHRP-ROC, MRMC HSRRB and USAMMDA-RA within one working day of receiving notice of event. All study vaccinations are held until further notice.

Follow-Up and Resolution:
Protocol Safety Team performs review of safety data and determines if permanent discontinuation of vaccination is appropriate. Consult FDA, if needed. USAMMDA-RA notifies FDA of decision.

Toxicity grades are taken from *DAIDS Tables for Grading the Severity of Adult Adverse Experiences* (revised September 2002). To meet the pause criteria described below, all symptoms and adverse experiences must be *vaccine related*. *Vaccine related* means the experience is judged to be *possibly, probably* or *definitely related* to the study vaccination.

The study vaccinations and enrollments would resume only if review of the adverse experiences by the FDA and IND sponsor resulted in a recommendation to permit further study vaccinations and study enrollments.

5.2 Adverse Experiences

An adverse experience is any unfavorable or unintended change in body structure, body function or laboratory result associated temporally with the use of study treatment, whether or not considered related to the study treatment. Each adverse experience will be graded according to the Table for Grading Severity of Adverse Experiences in Attachment V. In general, DAIDS guidelines and procedures will be utilized. However, an additional category for relating the vaccine to an AE has been added.

If the experience is not listed the following guidelines will be applied:

TABLE 8. GENERAL TOXICITY GRADING GUIDELINES

**Mild**	(Grade 1)	Transient or mild discomfort.<br>No limitation in normal daily activity.
**Moderate**	(Grade 2)	Some limitation in normal daily activity.
**Severe**	(Grade 3)	Unable to perform normal daily activity.
**Serious**	(Grade 4)	Life threatening.
**Death**	(Grade 5)	

The **relationship of an adverse experience (AE) to vaccination** will also be determined by the investigator, based on the following definitions:

Not Related:
- AE obviously explained by another cause; ***OR***
- The time of occurrence of AE is not reasonably related to vaccination.

Remotely Related:
- AE more likely explained by causes other than vaccination.

Possibly Related:
- Vaccine administration and AE occurrence reasonably related in time; ***AND***
- AE explained equally well by causes other than vaccination.

Probably Related:
- Vaccine administration and AE occurrence reasonably related in time; ***AND***
- AE more likely explained by vaccination than by other mechanisms.

Definitely Related:
- Vaccine administration and AE occurrence reasonably related in time; ***AND***
- Vaccination most likely explains the AE; ***AND*** AE is consistent with pattern of vaccine-related experiences.

Adverse experiences will be managed in accord with good medical practice. All patients experiencing adverse experiences, regardless of severity, will be followed until satisfactory resolution, return to baseline, or until the toxicity is presumed to be irreversible.

5.3 Serious Adverse Experiences

The term "Serious Adverse Drug Experience (or event) (SAE)" is defined in 21 CFR 312.32 as follows: "Any adverse drug experience occurring at any dose that results in any of the following outcomes: Death, a life-threatening adverse drug experience, inpatient hospitalization or prolongation of existing hospitalization, a persistent or significant disability/incapacity, or a congenital anomaly/birth defect."

Important medical experiences that may not result in death, be life-threatening, or require hospitalization may be considered a serious adverse drug experience when, based upon appropriate medical judgment, they may jeopardize the subject or require medical or surgical intervention to prevent one of the outcomes listed in this definition.
Examples of such medical experiences include allergic bronchospasm requiring intensive treatment in an emergency room or at home, blood dyscrasias or convulsions that do not result in inpatient hospitalization, or the development of drug dependency or drug abuse.

A SAE that is "life threatening" refers to an adverse experience that at occurrence represented an immediate risk of death to the subject. An experience which may have caused death had it occurred in a more severe form is not considered life threatening. Similarly, a hospital admission for an elective procedure is not considered a Serious Adverse Experience.

5.4 Adverse Experience Reporting

Information on adverse experiences is collected by the Research Nurse and other clinic staff and entered into a computer database. These data are reviewed on an ongoing basis by the study coordinator and the Principal investigator. In addition, regular weekly meetings are held in the clinic amongst members of the protocol safety team [composed of the PI, AI, DAIDS MO, and medical monitor. Additional participants could include co-investigators and senior clinical research nursing staff. A quorum of three members of the safety team will be required for study modification.

A reportable AE is an experience, which will require modification of the study (i.e. fulfills a stopping rule), or is any Grade 3 or 4 experience, and is possibly, probably or definitely related to study product or is a SAE. This team will review all SAE, all reportable AE and summary safety data. In addition, the clinical database will run an automated program to review all available data on a daily basis to notify the protocol safety team via email of any potential reportable adverse experiences that were not recognized by the investigative team and to identify experiences that should trigger the pause rules. Notification of reportable AE by email or telephone is required as soon as possible after recognition of the experience.

5.5 Serious Adverse Experience Reporting

MRMC HSRRB requires expedited reporting for all adverse experiences, which are both serious and unexpected as defined in the International Conference for Harmonization (ICH) guidelines. This requirement should be fulfilled through the reporting pattern noted above. However, if an experience arises which fulfills the criteria of unexpected (not in the investigators brochure) and is serious, the PI will provide a report to USMHRP-ROC as if it was a reportable AE. USMHRP-ROC will forward the report to HSRRB, WRAIR HURC, and the Sponsor Representative. SAE reports on Thai participants will also be forwarded to the applicable Thai IRBs.

Unanticipated problems involving risk to volunteers or others, serious adverse events related to participation in the study, and all subject deaths should be promptly reported by telephone (301-619-2165), by email (hsrrb@amedd.army.mil), or by facsimile (301-619-7803) to the Army Surgeon General's Human Subjects Research Review Board (HSRRB). A complete written report should follow the initial notification. In addition to the methods above, the complete report can be sent to the U.S. Army Medical Research and Materiel Command, ATTN: MCMR-ZB-PH, 504 Scott Street, Fort Detrick, Maryland 21702-5012.

Briefly summarized, experiences requiring completion of an SAE report form include the following:

5.5.1 Regardless of assessed relationship to study agent, report:

- Death
- Life threatening experience (i.e. the subject was in the opinion of the investigator, at immediate risk of death from the experience)
- Congenital anomaly/birth defect or spontaneous abortion
- Permanent disability/incapacity
- An experience that requires or prolongs inpatient hospitalization (defined as greater than 24 hours)
- An experience that is important and significant medically based up one's appropriate medical judgment, that may endanger the subject and may require medical or surgical intervention to prevent the other outcomes defined above.

5.5.2 Up to six months after last dose of study agent, report:

- Grade 3 or 4 adverse experiences if the relationship to study agent is assessed as definitely, probably or possibly related to study agent.

- Any experience, regardless of grade, which in the judgment of a site investigator represents a serious adverse experience, may be reported.

5.6 Adverse Experience Reporting to the Institutional Review Board

5.6.1 Local Medical Monitor:

The WRAIR HURC and HSRRB regulations require that a local medical monitor must be assigned to this study. The role and qualifications of the medical monitor are identified as follows:

The name and *curriculum vitae* of the medical monitor will be provided to the HURC and HSRRB. This individual should be a qualified physician, other than the Principal Investigator, not associated with this particular protocol, able to provide medical care to research volunteers for conditions that may arise during the conduct of the study, and who will monitor the volunteers during the conduct of the study. The medical monitor is required to review all serious and unexpected adverse experiences (per ICH definitions) associated with the protocol and provide an unbiased written report of the experience within 10 calendar days of the initial report. At a minimum, the medical monitor should comment on the outcomes of the adverse experience (AE) and relationship of the AE to the test article. The medical monitor should also indicate whether he/she concurs with the details of the report provided by the study investigator.

5.6.2 Reporting Serious and Unexpected Adverse Experiences to the US Army IRB:

Reporting requirements for this institution and the local WRAIR IRB conform to ICH terminology and guidance. The USMHRP ROC is responsible for fulfilling adverse experience reporting requirements.

Adverse events that are both serious and unexpected will be immediately reported by telephone to the USMHRP ROC and they in turn will report to the U.S. Army Medical Research and Materiel Command (USAMRMC) Office of Research Protections. All unanticipated problems involving risk to subjects or others, serious adverse events related to participation in the study, and subject deaths related to participation in the study should be promptly reported by telephone (301-619-2165), by e-mail (hsrrb@amedd.army.mil), or by facsimile (301-619-7803) to the USAMRMC, Office of Research Protections, Human Research Protection Office. A complete written report will follow the initial notification. In addition to the methods above, the complete report will be sent to the U.S. Army Medical Research and Materiel Command, ATTN: MCMR-ZB-PH, 504 Scott Street, Ft. Detrick, MD 21702-5012.

Follow-up information should be provided when available. All serious adverse event reports should include the study title and HSRRB Log No., description of event, subject status, any actions taken in response to the event, and the name of the individual making the report and his/her relationship to the study. Telephonic reporting must be followed by a written report within 24 hours to the Local Medical Monitor and forwarded to the Office of Research Protection.

Serious and unexpected adverse events may be categorized as likely related to the test article (possibly, probably, or definitely related) or unlikely to be related (denoted as remotely related or “probably not related” by the DAIDS Manual) or unrelated.
All AEs that are both serious and unexpected and related to the product are reported as described above. Serious and unexpected adverse events which are unlikely to be related to the test article and are NOT within the expected life experience of the general population will be listed at regular intervals, reviewed by the local medical monitor and reported to the ORP/HSRRB, and the sponsor.

A written report by the Principal Investigator and a written concurrence and/or commentary by the Local Medical Monitor to include the study title and HSRRB Log No, description of events, subject status, any actions taken in response to the event and their relationship to the study will be submitted annually.

USA Local Medical Monitor:
Paul Scott, M.D. MPH
WRAIR
One Taft Court
Rockville, MD 20850 USA
Phone: 301-251-8339
Email: pscott@hivresearch.org

Thai Local Medical Monitors:
Dr. Krisada Jongskul
Dept. of Immunology & Medicine, AFRIMS
315/6 Rajvithi Road
Bangkok, 10400
Phone: 02644 4888 ext. 2684
Email: kusadaj@afrims.org

Winai Ratanasuwan, MD, MPH
Dept. of Preventative & Social Medicine
Faculty of Medicine, Siriraj Hospital
Bangkok, 10700
Phone: 622-411-3019, 419-7284
FAX: 622-411-5034

6 STATISTICAL CONSIDERATIONS

6.1 Study Overview

The primary aim of this phase I double-blinded, randomized study is to assess Safety and Immunogenicity of WRAIR/NIH Live Recombinant MVA-CMDR (HIV-1 CM235 env/ CM240 gag/pol) administered by the Intramuscular (IM) or Intradermal (ID) Route in healthy, HIV-uninfected Adults.

All data from enrolled volunteers will be analyzed according to the initial randomization assignment regardless of how many study vaccinations they receive.

The Statistical and Data Management Center for the study will be the Data Coordinating and Analysis Center (DCAC) at the USMHRP site in Rockville, MD. Data management quality control will be performed by DCAC and final study analysis will be performed jointly.

6.2 Primary and Secondary Endpoints

6.2.1 Primary Endpoints, Safety:

Safety assessments will be made throughout the trial to evaluate local toxicity and systemic reactions by means of clinical evaluation and laboratory tests (hematology, blood chemistry, and urinalysis).

Safety endpoints will include the following:

- Severe local toxicity at injection site
- Severe systemic symptoms judged to be probably or definitely related to the vaccine.

6.2.2 Secondary Endpoints, Immunogenicity:

6.2.2.1.1 Humoral

- Serum Binding (ELISA) antibodies to HIV-1 Subtype E and B envelope antigens
- Serum Binding (ELISA) antibodies Subtype A and B p24 antigens.

6.2.2.1.2 Cellular

- CTL responses to Subtype E and B envelopes, gag, and pol targets expressed in (or loaded onto) EBV transformed autologous B cell lines.
- IFN-ϒ ELISPOT, and intracellular cytokine (ICC) responses using a panel of env and gag E and B clade-derived peptides.
- Lymphocyte proliferation to relevant clade A, E, and B HIV antigens (env, gag and pol).

6.3 Sample Size, Accrual, and Dose-Escalation Guidelines

Recruitment will target 48 healthy, low-risk, HIV-1 uninfected adults. Volunteers will be enrolled within three months (90 days) prior to the first vaccination for the screening process.

Each volunteer meeting inclusion criteria will be randomized to vaccine or placebo in a 5:1 ratio. Participants will be assigned to Part A and B sequentially as they are enrolled into the study. Part A and B are similar in design that there is a 50/50 chance of getting either route (IM or ID).

With respect to route there is a lower dose, (Part A) and a higher dose (Part B) component of the study. Half will be enrolled into the lower dose and half to the higher dose (with respect to route) depending on the timing of enrollment. Randomization is assigned by means of a randomization list generated by the study statistician and provided to the site pharmacist. For safety reasons, the volunteers will be enrolled into Parts A and B in a staggered fashion.

- In Part A, Groups I and II (12 in each group) receiving the lower doses (10^6 and 10^7 pfu or either vaccine or placebo) will be enrolled first. Ten volunteers in each group will receive vaccine and two will receive placebo.

- In Part B, Groups III and IV (12 in each group), receiving the higher dose (10^7 and 10^8 pfu of either vaccine or placebo) will be enrolled after the first two vaccinations of the Part A Groups are completed and shown to be safe and well-tolerated. Ten volunteers in each group will receive vaccine and two will receive placebo.

- A third vaccination will be given at Day 84 for all study volunteers.

Each vaccination will be followed with a phone call (within 24-48 hours) and a safety visit (within 14 days). Two other immunogenicity visits and one follow-up for lab results only will complete the trial schedule of eleven visits over a 12-month period. This study will provide new, preliminary data regarding the safety and immunogenicity of intramuscular and intradermal MVA-HIV recombinant vaccine candidates.

Sample size calculation for safety: the following table gives the probability of observing at least one Severe Adverse Experience (SAE) with true toxicity of 5%, 10% or 20% for different sample sizes. For example, if the true rate of adverse experiences is 20% in the population, the probability of observing at least one adverse experience is 0.89 for each of the vaccine arms (N=10) and 0.98 for the combined arms (N=20) of either group A or group B. If, however, the true rate of occurrence of a given toxicity is 5% then the probability that at least one adverse experience is observed is 0.4 for each vaccine arm and .64 for the combined groups of A or B. The probability of observing at least one SAE in the different vaccine arms is adequate.

TABLE 9. Probability of Observing at least one
Severe Adverse Experience (SAE) with True Toxicity of 5%, 10% or 20%

True toxicity	N=5	N=10	N=20
5%	.23	.40	.64
10%	.41	.65	.87
20%	.67	.89	.98

6.4 Statistical Plan: Design & Analysis

The data will be analyzed by DCAC. Any data analyses carried out independently by investigators should be submitted to the DCAC before publication or presentation. The primary safety measurements include data from the clinical laboratory tests, observed vaccination reactions (local and systemic) and adverse experiences. The immunogenicity data include serum antibody responses to vaccine antigens as evaluated by both ELISA and neutralizing antibody assays. Cellular responses will be assayed by both blastogenesis (lymphocyte proliferation) and CTL, ICC, and ELISPOT assays.

All volunteers receiving at least one vaccination of vaccine will be included in the safety and tolerability analyses. Volunteers will be excluded from immunogenicity analyses at each time point if their blood draw occurs more that 14 days outside a visit window. If the subject visit is more than 14 days outside of the study visit window, volunteers will be excluded, unless an exceptional situation exists (and will be reviewed on a case by case basis). All data exclusions, including premature terminations, will be detailed and tabulated according to vaccine group and site. Data listings will include all volunteers.

Demographic data obtained during the baseline visit will be listed for each subject. Summary statistics will be tabulated for each vaccine group and for each site. Volunteers will be assessed for comparability at baseline. Descriptive statistics will be presented for continuous variables (age, height, weight). Frequency counts and percents will be presented for categorical variables (sex and ethnicity).

Non-parametric tests will be used to evaluate NAb and binding Ab titers. CTL responses will be compared using the chi square analysis, with the presence or absence of cumulative CTL responses being positive or negative.

Statistically significant results will be annotated by their degree (*p<0.05;**p<0.01;***p<0.001). Statistical significance will be considered if $p < 0.05$.

6.5 Safety

All volunteers receiving a vaccination of vaccine will be included in the safety and tolerability analyses.

6.5.1 Post vaccination Reactions (Local and Systemic)

The maximal severity of local and systemic post-vaccination reactions occurring in the seven days following each vaccination and will be tabulated by group. Local reactions tabulated will include: injection site pain, erythema, induration, and temperature.

Systemic reactions reported within seven days post-vaccination will include: chills, nausea, malaise, myalgia, arthralgia, headache, rash and fever (oral temperature greater than 38ºC (100.4ºF). Additionally, pain on injection will be noted. Frequencies and percentages of volunteers experiencing each reaction will be presented for each symptom severity. If a reaction occurs more than once for a subject, the reaction will be classified according to the highest occurring severity and closest vaccine relationship. Summary tables showing the occurrence of any local or systemic reaction overall and at each time point will also be presented.

6.5.2 Other Adverse Experiences

The original terms used by investigators on the CRF will be translated to MeDRA (Medical Dictionary for Regulatory Activities) terms and grouped into body system and presented as frequency tables. For each and any vaccination, the number and percent of volunteers with adverse experiences will by tabulated, by group and by site. When adverse experiences occur more than once, the maximal severity and relationship to vaccine will be counted.

Three summaries will be generated:

- Serious adverse experiences (SAEs)
- Adverse experiences that are definitely, probably or possibly related to vaccination.
- Adverse experiences that are remotely or not related to vaccination.

Data listings of all adverse experiences will be provided by subject, as will subset listings summarizing volunteers withdrawn from the study because of an adverse experience.

6.5.3 Clinical Laboratory

Listings and descriptive statistics will be generated for all laboratory parameters that are abnormal, by group and site. Data listings will be provided by subject summarizing all abnormal values, by parameter (WBC count, hemoglobin, platelet count, ALT, creatinine, U/A).

6.5.4 Immunogenicity

For each group, least squares geometric mean titer (GMTs), associated 95% confidence intervals, and median, minimal and maximal titer will be calculated for ELISA antibody titers to HIV-1 Subtype B gag p24 as well as to env antigens.

Lymphocyte proliferation will be described as a stimulation index (LSI = CPM/Medium Control).

For each group (and site), geometric mean LSI, median minimal and maximal LSI, and associated 95% confidence intervals will be calculated. The primary time point of interest for antibody responses will be two weeks post vaccination.

The following cellular response definitions establish discrete categorical data (response present versus absent) for significance testing:

- >60% of recipients develop an LSI ≥ 5 to HIV-specific vaccine component at two time points post vaccination
- >30% of recipients develop CTL to HIV-specific vaccine components at 1 time point with % specific lysis ≥ 10% at two dilutions at a single time point or separately at two separate time points (cumulative % positive will be calculated as % positive [vaccinees] minus % positive [controls] at baseline).

Chi-square and Fisher's exact tests will be employed. All tests will be conducted as two-sided at a 0.05 significance level. Statistically significant results for humoral responses will be annotated by their degree (*p<0.05; **p<0.01; ***p<0.001). The study is descriptive and exploratory in nature and NOT powered to detect significant differences in cell-mediated immune responses.

The statistical analysis for immunogenicity will employ the as-treated principle wherein fully immunized participants will be analyzed separately from those with incomplete vaccination. In addition, analysis will be performed using the intent-to-treat principle whereby all data from enrolled volunteers will be used according to the initial randomization assignment regardless of how many study vaccinations they receive. The only exception will be to exclude data from HIV-infected participants at or post infection. If a participant's infection time cannot be determined, all data from the participant will be excluded from the analysis. Only participants completing all vaccinations will be included in the immunogenicity analysis.

6.6 Data Monitoring

Data monitoring and related activities will be performed by USAMMDA and USMHRP ROC.

7 PHARMACY PROCEDURES

7.1 Protocol Schema

This is a double-blind safety study. Volunteers in all dose groups will receive three IM or ID injections on Days 0, 28, 84 as outlined below:

TABLE 10. Study Schema

Part A	Subjects	Vaccination Schedule		
Group	Vaccine/Placebo[1]	Day of the Study in Part A		
		0	28	84
I	10/2	10^7 pfu IM, 1.0 mL into deltoid muscle		
II	10/2	10^6 pfu ID, 0.1 mL into the volar aspect of the forearm to produce a wheal		
Review of Safety Data for First and Second Vaccinations of Part A Groups				
Part B	Subjects	Vaccination Schedule		
Group	Vaccine/Placebo	Day of the Study in Part B		
		0	28	84
III	10/2	10^8 pfu IM, 1.0 mL into deltoid muscle		
IV	10/2	10^7 pfu ID, 0.1 mL into the volar aspect of the forearm to produce a wheal		

[1]Vaccine: MVA-CMDR ; Placebo: vaccine diluent consists of PBS (without Ca^{2+} and Mg^{2+}), with 7.5% lactose , pH 7.4.

Volunteers will be screened up to 90 days prior to the first vaccination. Volunteers will be randomized to vaccine or placebo in a 5:1 ratio (see Schema). For safety reasons, volunteers will be enrolled into in a staggered fashion: Part A, Groups I and II receiving the lower doses will be enrolled first; and Part B, Groups III and IV, receiving the higher doses will be enrolled after the first two vaccinations of the Part A are shown to be safe and well-tolerated.[1]

7.2 Vaccine Description and Preparation

MVA-CMDR, produced by WRAIR/NIH, is a live recombinant poxvirus vector (Modified Vaccinia Ankara) vaccine that has been genetically engineered to express the following HIV-1 genes: gp160 (Subtype E, CM235) and gag and pol (integrase deleted and reverse transcriptase rendered non-functional (Subtype A, CM240).

MVA-CMDR (1 x 10^9 IU/mL) 0.4 mL/2 mL vial, Lot # 0965 and MVA-CMDR (1 x 10^7 IU/mL) 1.2 mL/2 mL vial, Lot # 0966. Each vial is for single-dose use and will be given a label containing the following information; vaccination name, dosage, BPR number, lot number, contents, storage directions, caution warning, manufacturer, and date of manufacturing.

Vaccine will be administered by intramuscular (IM, 1 mL) injection into deltoid muscle, or intradermal injection (ID, 0.1 mL) into the volar aspect of the forearm to produce a wheal (See Study Schema).

After consultation of the randomization list and selection of the vial, Site Pharmacist (SP) will record the sequence # (from the randomization list), the group number, and the number of the MVA vial # in the Vaccine Preparation Worksheet (VPW). The following procedure will be used in the preparation of the vaccine doses:

- All work must be done in a Bio Safety Cabinet (BSC).
- Thaw MVA-CMDR vials at room temperature and then stored at 2˚ to 8˚ C in secure refrigerator.
- Mix MVA-CMDR vials vigorously using high-speed vortex for 60 seconds prior to withdrawal of the vaccine into the syringe.
- From MVA-CMDR (10^7 pfu/mL) and using aseptic technique, withdraw
  - 1 mL of the above vaccine into a 1 mL syringe for Group I
  - 0.1 mL into a 1 mL syringe for Group II.

- From MVA-CMDR (10^9 pfu/mL) and using aseptic technique,
  - Withdraw and transfer 0.15 mL of MVA-CMDR (10^9 pfu/mL) into 10 cc mixing vial, add 1.35 mL of diluent and mix for 15 seconds. Withdraw 1 mL of the above vaccine into a 1 mL syringe for Group III.
  - Withdraw and transfer 0.05 mL of MVA-CMDR (10^9 pfu/mL) into 10cc mixing vial, add 0.45 mL of diluent and mix for 15 seconds. Withdraw 0.1 mL into a 1 mL syringe for Group IV.
- Upon completion of the procedure, record the time/date on the VPW and sign the VPW.
- On six syringe labels record the protocol number, subject number/initial, vaccination number, date and time when the syringe was loaded.
- Affix one label on the syringe, one on the transportation bag, two labels in the transportation bag for the PI or designee to affix to the CRF and source
- documentation, the fifth label will be affixed to the Syringe Dispensing Worksheet and the sixth one will be affixed on the used vial bag.
- Place the syringe in the transportation bag and give to the PI or designee.
- Record time/date of dispensing and sign Vaccine Dispensing Worksheet.
- If not used immediately the syringe must be stored at 2 to 8˚ C but must be used within 1 hour of preparation.

7.3 Placebo Description and Preparation

Vaccine diluent consists of PBS (without Ca^{2+} and Mg^{2+}), with 7.5% lactose, pH 7.4, manufactured by the WRAIR PBF under GMP manufacturing conditions. This vaccine diluent will be the placebo for this trial. The product will be stored as indicated on the vial. Each vial is for single-dose use and will be given a label

containing the following information; vaccination name, dosage, BPR number, lot number, contents, storage directions, caution warning, manufacturer, and date of manufacturing. Placebo will be administered by intramuscular (IM, 1 mL) injection into deltoid muscle, or intradermal injection (ID, 0.1 mL) into the volar aspect of the forearm to produce a wheal (See Study Schema).

SP will record the sequence number (from the randomization list) in the VPW. SP will also record the group number and the number of the PBS lot.

The following procedure will be used in the preparation of the placebo:

- On six labels, record the protocol number, subject number/initial, vaccination number, date and time when the syringe was loaded.
- All work must be done in a BSC.
- Placebo vial should be gently swirled prior to withdrawal of the placebo dose.
- Using aseptic technique, withdraw:
    - 1 mL of diluent into a 1 mL syringe for Groups I and III
    - 0.1 mL into a 1 mL syringe for Groups II and IV.
- Upon completion of the procedure, record the completion time/date and sign the VPW.
- Take the six syringe labels and affix one label on the syringe, one on the transportation bag, two labels in the transportation bag for the PI or designee to affix to the CRF and source documentation, the fifth label will be affixed to the Syringe Dispensing Worksheet and the sixth one on the used vial bag.
- Place the syringe in the transportation bag and give to the PI or designee.
- Record time/date of dispensing and sign Vaccine Dispensing Worksheet.
- If not delivered immediately, the syringe must be stored at 2 to 8˚ C but must be used within 1 hour of preparation.

7.4 Study Product Acquisition/Disposition

The SP will be responsible for receiving the investigational product and other study materials, which will be stored in a secure location under specified conditions at the VCRC/WRAIR in Rockville, MD. The SP will file appropriate records of shipment and receipt, must visually inspect all vaccine preparation materials for appearance and integrity and will provide the blinded prepared syringe in the transportation bag to the investigator or designee for administration.

Any materials showing evidence of damage or compromised integrity of the container/closure system will not be used for any vaccine preparation. The suspect vials will be kept in quarantine.

At the conclusion of vaccinations, all supplies (including used, unused or partially used vials and unused or partially used syringes of MVA-CMDR) will be

maintained at the VCRC/WRAIR for destruction. Partially used vials may not be administered to other volunteers or be used for *in vitro* or animal model experimental studies.

The Thailand sites will utilize nurse pharmacists (clinical research nurses trained in preparation of vaccine and not involved with recruitment, enrolment, or conducting the study among volunteers) will be responsible for receiving the investigational product and other study materials at each site. The study product will be shipped to AFRIMS and maintained in a double-locked secure location under specified conditions (-80 C freezer). Upon initiation of the study, vaccine will be delivered on dry ice to the study sites and maintained in a double-locked secure location and maintained in clinical -86 C freezer until preparation. Nurse pharmacists will maintain records of shipment and receipt, visual inspection of all vaccine preparation materials for appearance and integrity, and will provide the blinded prepared syringe in the transportation bag to the investigator or designee for administration. Any materials showing evidence of damage or compromised integrity of the container/closure system will not be used for any vaccine preparation. The suspect vials will be kept in quarantine.

At the conclusion of vaccinations, all supplies (including used, unused or partially used vials and unused or partially used syringes of MVA-CMDR) will be returned back to AFRIMS and maintained until instructions for destruction are received from the study sponsor and PI.

7.5 Pharmacy Records

The SP is required to maintain complete records of all study products received from WRAIR/NIH and subsequently dispensed. The SP is also responsible for maintaining an accurate record of the randomization codes, inventory, and an accountability record of vaccine supplies for this study. The SP will also be responsible for insuring the security of these documents.

7.6 Procedures to Preserve Blinding

The SP/Pharmacy Tech is responsible for maintaining randomization codes for each participant in a confidential and secure manner. The randomization code will be generated by the study statistician and after generation will be held in a controlled fashion in the research pharmacy at clinical site.

Study assignment for each participant will also be contained in individual sealed envelopes labeled with study number only and securely stored by the data SP/ Pharmacy Tech and data manager in a locked file cabinet in a limited access storage area, to be available in case of emergency. Only the SP/ Pharmacy Tech will have a key to the locked file.

Volunteers will be randomized to receive either the investigational vaccine or placebo. The subject, the clinical staff, and the Principal Investigator will be

blinded to treatment allocation. The SP/ Pharmacy Tech with primary responsibility for drug dispensing maintains the randomization code and completes assignments of
participants according to the randomization allocation. Unblinding of volunteers may occur only after all participants have had their last study visit. Blinding will be limited to whether the subject receives the test vaccine or placebo.

The blind will be broken only if, in the opinion of the Principal Investigator or the Sponsor (OTSG), immediate unblinding is necessitated by an acute safety concern. If in the judgment of the Investigator and/or the Sponsor (OTSG), a participant has sustained an SAE that necessitates immediate unblinding, the PI or Sponsor may request unblinding for that subject. Such unblinding is performed for medical management of the SAE. This situation is expected to be extremely rare**.** The subject must not receive any further vaccination but will continue the visit schedule. The Sponsor must then treat the unblinding as a protocol violation and report it in the Pharmacy (Unblinding) logbook.

IMPORTANT NOTE:

THE SITE PERSONNEL SHOULD NEVER KNOW THE BROKEN CODE.

8 HUMAN SUBJECT PROTECTIONS AND ETHICAL OBLIGATIONS

8.1 Informed Consent

A template for the study informed consent is provided in AttachmentI. It describes the investigational product to be used and all aspects involved in protocol participation. A properly executed written site specific informed consent based on the template provided here, in compliance with the Declaration of Helsinki, guidelines of the Council of International Organization of Medical Sciences (CIOMS) and US law 21 CFR 50, must be obtained from each subject prior to entering the subject into trial or prior to performing any unusual or non routine procedure that involves risk to the subject. The investigator must provide a copy of the WRAIR HURC and HSRRB-approved informed consent to the subject and a signed copy must be maintained in the subject's record file. Before a subject's participation in the study, it is the investigator's responsibility to obtain this written informed consent from the subject, after adequate explanation of the aims, methods, anticipated benefits, and potential hazards of the study and before any protocol-specific procedures or study medications are administered.

The informed consent process for the participating Thai site described below will be utilized by both sites to ensure that the same practical standard is applied to the consenting process of potential participants in this study. The volunteer has to understand the purpose, information and procedures associated with the study, and the rights and responsibilities associated with participation.

The study staff (research nurses and/or site investigators) will explain to potential participants about the risks and benefits that may arise from participation in the study and clearly answer any questions the volunteer may have. The volunteer will then complete a test of understanding/comprehension in order to be eligible to participate. These same or similar procedures have been performed in 5 phase I or II HIV vaccine trials conducted by AFRIMS in Thailand, as well as more than 16,000 volunteers who were screened for participation in the phase III HIV vaccine trial on the eastern seaboard of Thailand. The demographic characteristics of some of these volunteers and their reasons for participation have been previously published [1-4]. The volunteer will be given ample time to make the decision to participate or not, an unsigned copy of the consent form may be given to the volunteer to review at home in order to allow adequate time for the volunteer to make his/her decision. If a volunteer agrees to participate in the study, the consent is signed in compliance with ICH E6 GCP guidelines.

Literacy will not be a factor for recruitment of volunteers in Thailand.

8.2 Risks and Benefits

8.2.1 Risks

The potential side effects resulting from intramuscular and intradermal injections include stinging, arm discomfort, or redness of the skin at vaccine injection sites. Study volunteers can receive medications such as acetaminophen, NSAIDs, or antihistamines as required.

Volunteers may exhibit general signs and symptoms associated with administration of a vaccine or placebo vaccination, including fever, chills, rash, aches and pains, nausea, headache, dizziness and fatigue. These side effects will be monitored, but are generally short term and do not require treatment.

Volunteers may have an allergic reaction to the vaccination. An allergic reaction may cause a rash, hives, or even difficulty breathing. While severe reactions are rare, to minimize this risk, those with allergies to or have had an allergic reaction to eggs, egg products, neomycin, or streptomycin will not be allowed to participate in this study. Medications are available in the clinic to treat serious allergic reactions.

Volunteers with a known allergy will be excluded from participation.

The effect of this vaccine on a fetus or nursing baby is unknown, so female volunteers of child bearing potential will be required to agree to use birth control for sexual intercourse beginning 60 days prior to the first vaccination and continuing through the last protocol visit. Women who are pregnant or nursing will be excluded from the study.

The vaccine may cause a positive HIV antibody test using the standard screening test. A positive or indeterminate test may have a negative employment and social impact, as a result a letter of participation will be provided on request (Attachment III).

Volunteers will be discouraged from donating blood during study participation as a result of the potential positive HIV antibody test result. If these test results should occur, Western blot analysis will be augmented with PCR or other required testing to either exclude or confirm HIV infection. Blood donation options for those volunteers who wish to resume blood donation will be explained at the final study follow-up visit.

Volunteers that require vaccination against tetanus, influenza, hepatitis A, hepatitis B and/or rabies during the study should receive vaccinations at least two weeks before or two weeks after the study injections. This is to minimize the risk of confusing a reaction to these vaccines with a reaction to the study vaccine. If

rabies or tetanus vaccination is required after an accident or animal bite, it must take priority over the study vaccination. Should a subject receive a rabies vaccination, they will not be given any more study vaccinations. However, they will continue to take part in study follow-up visits according to the schedule previously described.

Risk of myo/pericarditis
The MVA vaccine used in this study is related to the vaccine to prevent smallpox. A very small number of people who received the smallpox vaccine developed an inflammation of the heart muscle (myocarditis). Some people developed an inflammation of the sac surrounding the heart (pericarditis). The number of people who had these problems was very small (96 people out of 666,712 who received the vaccine). Investigators do not expect these side effects from the MVA vaccine because it is a much weaker virus and it cannot divide or multiply. Myocarditis has NOT been reported with previous MVA use. To further decrease the risk of myocarditis, volunteers will be required to undergo enhanced cardiac monitoring (ECGs and lab tests) during this study.

If any abnormal changes to the heart are found, the subject will not receive any more vaccinations. The study team will make the necessary referrals for diagnosis, treatment, and follow-up. Volunteers will continue to be followed for a minimum of one year after the problem is detected.

Risks from HLA testing
HLA results can be used to provide information on the susceptibility of a subject to certain disease. Used inappropriately this information could be discriminatory. HLA typing can also be used to determine paternity. However, the blood samples that are donated during study participation will not be used for this purpose.

Unknown Risks
There may be other serious risks that are not known.

Blood drawing may cause pain, bruising, and, rarely, infection at the site where the blood is taken.

Volunteers may believe that this vaccine provides protection, and therefore practice riskier behavior. They will receive extensive counseling throughout the study to address this potential problem.

8.2.2 Benefits

Although study volunteers may benefit from clinical testing and physical examination, they may receive no direct benefit from participation. Others may benefit from knowledge gained in this study that may aid in the development of an HIV vaccine.

8.3 Subject Confidentiality

The RV 158 Principal Investigator (Dr. Mary A. Marovich) will maintain research records of volunteer participation at the U.S. site for this study[6]. All volunteers will receive study numbers that are known only to the investigators and clinic staff. Clinical tests will be identified by PIN and study number and the specimen bag will be identified by bar code only. Clinical and research records may be reviewed by the representative of the U.S. Army Medical Research and Materiel Command (USAMRMC), USAMMDA representative, WRAIR HURC (Walter Reed Army Institute of Research Human Use Review Committee), representatives of the Human Subject Research Review Board (HSRRB), representatives of the Food and Drug Administration (FDA), and other regulatory agencies as part of their responsibilities for insuring the protection of research volunteers. Representatives of the Faculty of Medicine, Siriraj Hospital, Mahidol University and the Ministry of Public Health IRBs may review the clinical and research records of Thai participants.

Every effort will be made to keep the records as confidential as possible within the limits of the law. All data and medical information obtained about volunteers, as an individual will be considered privileged and held in confidence. Research and clinical information relating to volunteers will be shared with other investigators and the scientific community through presentation or publication; however, volunteers will NOT be identified by name or social security number.

All volunteers will receive an emergency card indicating their status as a vaccine trial participant. All volunteers will be counseled periodically regarding the potential for testing positive on routine screening tests for HIV-1 as a consequence of participation in this trial and receiving this vaccine product. All volunteers will be offered further confirmatory testing and certification as to the nature of their vaccine trial participation whenever needed to address complications arising at home, at work or in the community, which could arise from routine screening for HIV-1.

[6] The research records for the participating Thai site will be maintained onsite with over site maintained by the local investigator.

8.4 Volunteer Registry Database

It is the policy of the USAMRMC that Volunteer Registry Data Sheets are completed on all volunteers participating in research for entry into the Command's Volunteer Registry Database. The Volunteer Registry Data Sheets will collect the following data on the volunteers: names (first and last name), date of birth, home district, study name and study dates. These are the data to be entered into the Command's confidential Volunteer Registry Database.

Volunteers will be informed that this will be stored at USAMRMC for a minimum of 75 years.

AFRIMS maintains the volunteer registry database be maintained in Thailand in accordance with internal policy and meets the requirements specified AR70-25. This data is collected on a source document and maintained throughout the study and updated. It is also used to contact volunteers after study follow up is completed as required by Thai MoPH regulation. The reason to maintain this database in Thailand is due to the complexity of transliteration of Thai script, the need to maintain accurate records to ensure effective communication, and the sensitivity of maintaining this information outside of Thailand, which has been raised by the Thai MoPH Ethical Review Committee.

8.5 Participation of Children

Children are not eligible to participate in this clinical trial because it does not meet the guidelines for inclusion of children in research. These guidelines (45 CFR 46, Subpart D, 401-409) state the Department of Health and Human Services protections for children who participate in research. Generally, healthy children can be studied when the research is considered as "not greater than minimal risk." Children can be involved in research with greater than minimal risk only when it presents the prospect of direct benefit to the individual child or is likely to yield generalizable knowledge about the child's disorder or condition.

8.6 Compensation

Each volunteer will receive $100.00 after each blood draw, including the last visit, for a total of $1100.00 over a one-year study period as compensation for time and inconvenience.

Volunteers will be compensated for time lost from work (500 baht; the minimum daily wage in Thailand is 200 baht), travel (200 baht), meal expenses (200 baht), and any miscellaneous expenses incurred due to a study visit (e.g., child care, 100 baht) for a total of 1000 baht (approximately $25 USD). Unusual travel expenses may be reimbursed if a receipt is presented.

9 ADMINISTRATIVE AND LEGAL OBLIGATIONS

9.1 Designation of Roles and Responsibilities

Principal Investigator (PI): To promptly report changes or unanticipated problems in a research activity. Normally, changes may not be initiated without OTSG approval, except where necessary to eliminate apparent immediate hazards to the human subject or others, immediately report, by telephone, any serious or unexpected adverse experiences which occur to the human subject or others to the WRAIR Office of Research Management at 301-319-9940 during duty hours and 301-319-9019 after duty hours. The report will also be made to the Office of Research Protections (DSN 343-2165 or 301-619-2165) (non-duty hours call DSN 343-2165 and send information by facsimile to DSN 343-7803 or 301-619-7803). To promptly report any change of investigators. To prepare annual continuing review reports at intervals designated by the WRAIR Human Use Review Committee and HSRRB and a final report in accordance with Title 21, Code of Federal Regulations, Part 312.33. To immediately report to WRAIR HURC and HSRRB knowledge of a pending compliance inspection by the Food and Drug Administration (FDA) or other outside governmental agency concerning clinical investigation or research.

Associate Investigators (AI): To act for the Principal Investigator when this individual is not able to discharge their responsibilities owing to travel, leave, deployment, or other extenuating circumstances. To assist the PI in the all aspects of protocol execution.

Laboratory Investigators: To execute laboratory procedures in compliance with all relevant regulatory guidelines inclusive of specimen accession, processing, aliquoting, distribution and archiving. To execute laboratory testing for TTD and other clinical laboratory parameters.

Research Coordinators: To interface directly with protocol volunteers. To maintain records of contact, inclusive of sample flow sheets. To perform phlebotomy, assess and administer investigational drugs, and report directly to the PI.

9.2 Protocol Amendments and Study Termination

Only the sponsor may modify the protocol. Amendments to the protocol will be made only after consultation and agreement between sponsor and investigator. Amendments to this protocol that increase the risk to subjects or any other substantial changes must be forwarded to the HRPO for review and approval by the HSRRB prior to implementation. The only exception is where the investigator considers that a subject's safety is compromised without immediate action.

In these circumstances, immediate approval of the chairman of the HSRRB and IRB must be sought, and the investigator should inform the sponsor and the full boards within five working days after the emergency occurred.

All amendments will be submitted to the WRAIR ORM for HURC approval through the USMHRP ROC prior to implementation. After submission review and approval WRAIRHURC will forward any amendments to the HSRRB study record for HSRRB approval when necessary, (including but not limited to changes in the Principal investigator, inclusion/exclusion criteria, number of volunteers to be enrolled study sties, or procedures).

Each site PI will submit continuing review reports to their respective IRBs. A copy of the approved continuing review report and the local IRB approval notification must be submitted to the USAMRMC, HRPO as soon as these documents become available. A copy of the approved final study report and local IRB approval notification must be submitted to the USAMRMC, HRPO as soon as these documents become available.

Any deviation to the protocol that may have an effect on the safety of the subject or the integrity of the study must be reported to the USAMRMC, HSRRB, at the Human Research Protection Office (HRPO) as soon as the deviation is identified.

9.3 Study Documentation and Storage

Source documents are original documents, data, and records from which the subject's data are obtained. These include but are not limited to hospital records, clinical and office charts, laboratory and pharmacy records, diaries, microfiches, radiographs, and correspondence.

The investigator and staff are responsible for ensuring maintenance of a comprehensive and centralized filing system of all study-related (essential) documentation, suitable for inspection at any time by representatives from the WRAIR, DAIDS, HSRRB, FDA, and/or applicable regulatory authorities.

Elements include:

- Subject files containing completed informed consent forms, and supporting copies of source documentation (if kept)
- Study files containing the protocol with all amendments, Investigator Brochures, copies of all correspondence with the IRB, DAIDS, and WRAIR.

In addition, all original source documentation must be maintained and be readily available.

Because data from clinical trials sponsored by USAMMDA may be used to support regulatory filings in several countries throughout the world, the policy concerning record retention reflects the most stringent current guidelines (those of the Committee for Proprietary Medicinal Products, or CPMP, in Europe).

To comply with the CPMP guidelines, WRAIR requests that the investigator arrange for the retention of case report forms, source records, and other supporting documentation for a minimum of 15 years.

9.4 Study Monitoring and Data Collection

All aspects of the study will be carefully monitored by the sponsor or authorized

representatives of the sponsor, with respect to current GCP and SOPs for compliance with applicable government regulations. These individuals will have access, both during the trial and after trial completion, to review and audit all records necessary to ensure integrity of the data, and will periodically review progress of the study with the PI.

All safety data from the Thai sites will be collected on Case CRFs and faxed to DCAC upon completion of the study visit. Other CRF's may be monitored, then a duplicate copy removed from the NCR (define) and hand-carried or mailed to Rockville.

Data obtained in the conduct of this study are housed in a secure database maintained by the Data Coordinating and Analysis Center (DCAC), Division of Retrovirology, WRAIR. All research data are entered in a secure database, using a double-data entry process, with standardized quality assurance review procedures in accordance with Good Clinical Practices.

The Data Coordinating and Analysis Center (DCAC) serves as the central data management facility for U.S. Military HIV Research Program (USMHRP) research protocols. Data managed by DCAC are entered into and maintained in a password-protected Oracle database called the Research Support System (RSS).

Data are accessible through RSS only to those DCAC and Information Technology staff authorized to work on the protocol. The RSS is located at the Rockville campus of the USMHRP and is protected by a firewall.

This data does not contain participant names or Social Security but is referenced only by the PIN or other study specific identification code.

Every attempt must be made to follow the protocol and to obtain and record all data requested for each subject at the specified times.

However, ethical reasons may warrant the failure to obtain and record certain data, or to record data at the times specified. If this becomes necessary, the reasons for such must be clearly documented on the case report form.

The Regulatory Office (ROC) considers all protocol departures and deviations protocol violations. Any deviations that impact safety or study integrity are reported promptly. All deviations are captured and included in the CRR and final study report for the HURC and the HSRRB.

Analysis files are created on a periodic basis and made available to the Principal Investigator (PI) and Clinical Research Coordinators (CRC) at the direction of the PI. Other collaborators may be given access to these analysis files, or data gathered from them, at the direction of the PI. Data may be made available as a listing, external file, or through a query program.

9.5 Policy Regarding Research-Related Injuries

U.S. Participants:

Should a volunteer be injured as a direct result of study participation, he/she will be provided medical care for that injury at no cost to the volunteer. The volunteer will only be treated for injuries that are directly caused by the research study. The Army will not pay for transportation to and from the hospital or clinic. However, United States Government will not provide long-term medical care or financial compensation for research related injuries.

Thai Participants:

Should a volunteer be injured as a direct result of study participation, he/she will be provided medical care for that injury at no cost to the volunteer. The volunteer will only be treated for injuries that are directly caused by the research study. The study will not pay for transportation to and from the hospital or clinic. No additional compensation will be available.

9.6 Use of Information and Publication

It is understood by the investigator that the information generated in this study will be used by the sponsor in connection with the development of the product and therefore may be disclosed to government agencies in various countries. To allow for the use of information derived from the study, it is understood that the investigator is obliged to provide the sponsor with complete test results, all study data, and access to all study records.

WRAIR recognizes the importance of communicating medical study data and therefore encourages their publication in reputable scientific journals and at seminars or conferences.

Any results of medical investigations and/or publication/lecture/manuscripts based thereon, shall be exchanged and discussed by the investigator, the manufacturers representative(s) and the U.S. Army Medical Research and Materiel Command 60 days prior to submission for publication or presentation. In cases of publications or presentations of material rising from multi-center clinical investigations, the sponsor is to serve as coordinator and referee. Individual investigators who are part of a multi-center investigation may not publish or present data that is considered common to a multi-center investigation without the consent of the other participating investigators and the prior review of the manufacturers and the U.S. Army Medical Research and Materiel Command. In case of disagreement among the investigators participating in the investigation, WRAIR will be the final arbiter. Results from investigations shall not be made available to any third party by the investigating team outside the publication procedure as outlined previously.

WRAIR will not quote from publications by investigators in its scientific information and/or promotional material without full acknowledgment of the source (i.e., author and reference).

9.7 Conduct of the Research Study

This research study will be conducted in accordance with Good Clinical Practice (GCP), International Conference on Harmonization (ICH) guidelines, and the revised US Code of Federal Regulations.

10 REFERENCES

1. UNAIDS/WHO. Report on the global HIV/AIDS epidemic. December 2002.

2. Quinn TC. Global burden of the HIV pandemic. *Lancet*.1996;348:99-106.

3. Moss B. Genetically engineered poxviruses for recombinant gene expression, vaccination, and safety. PNAS 1996; 93; 11341-8.

4. Tartaglia J, Excler JL, El Habib R, Limbach K, Meignier B, Plotkin S, Klein M. Canarypoxvirus-based vaccines: prime-boost strategies to induce cell-mediated and humoral immunity against HIV. AIDS Res Hum Retroviruses 1998; 14 (supp.3): S291-S298.

5. Girard M, Excler JL. Human Immunodeficiency virus. In Vaccines. Plotkin SA and Mortimer EA Eds. Saunders, Philadelphia, 1999, pp.928-967.

6. Excler J-L, Plotkin S. The prime-boost concept applied to HIV preventive vaccines. AIDS 1997; 11 (suppl A): S127-137.

7. Redfield RR, Wright DC, James WD, Jones TS, Brown C, Burke DS. Disseminated vaccinia in a military recruit with human immunodeficiency virus (HIV) disease. N Engl J Med.1987; 316: 673-676.

8. Sutter G, Moss B. Nonreplicating vaccinia vector efficiently expresses recombinant genes. PNAS 1992; 89: 10847-10851.9.

9. Sutter G, Moss B. Novel vaccinia vector derived from the host range restricted and highly attenuated MVA strain of vaccinia virus. Dev Biol Stand 1995; 84: 195-200.

10. Moss B, Carroll MW, Wyatt LS, Bennink JR, Hirsch VM, Goldstein S, Elkins WR, Fuerst TR, Lifson JD, Piatak M, Restifo NP, Overwijk W, Chamberlain R, Rosenberg SA, Sutter G. Host range restricted, non-replicating vaccinia virus vectors as vaccine candidates. IN Novel strategies in design and production of vaccines Cohen S & Shafferman A Eds., Plenum Press , New York, 1996: pp. 7-13.

11. Meyer H, Sutter G, Mayr A. Mapping of deletions in the genome of the highly attenuated vaccinia virus MVA and their influence on virulence. J Gen Virol 1991; 72: 1031-8.

12. Antoine G, Scheiflinger F, Forner F, Falkner FG. The complete genomic sequence of the modified vaccinia Ankara strain: comparison with other orthopoxviruses. Virology 1998; 244: 365-396.

13. Carroll MW, Moss B. Host range and cytopathogenicity of the highly attenuated MVA strain of vaccinia virus: propagation and generation of recombinant viruses in a nonhuman mammalian cell line. Virology 1997; 238: 198-211.

14. Drexler I, Heller K, Wahren B, Erfle V, Sutter G. Highly attenuated modified vaccinia virus Ankara replicates in baby hamster kidney cells, a potential host for virus propagation, but not in various human transformed and primary cells. J Gen Virol 1998; 79: 347-352.

15. Blanchard TJ, Alcami A, Andrea P, Smith GL. Modified vaccinia virus Ankara undergoes limited replication in human cells and lacks several immunomodulatory proteins: implications for use as a human vaccine. J Gen Virol 1998; 79: 1159-67.

16. Engleka KA, Lewis EW, Howard BH. Mechanisms of replication-deficient vaccinia virus/T7 RNA polymerase hybrid expression: effect of T7 RNA polymerase levels and alpha-amanitin. Virology 1998; 243: 331-339.

17. Symons JA, Alcami A, Smith GL. Vaccinia virus encodes a soluble type I interferon receptor of novel structure and broad species specificity. Cell 1995; 81: 551-560.

18. Alcami A, Smith GL. Vaccinia, cowpox, and camelpox viruses encode soluble gamma interferon receptors with novel broad species specificity. J Virol 1995; 69: 4633-9.

19. Alcami A, Smith GL. A soluble receptor for interleukin-1β encoded by vaccinia virus: a novel mechanism of virus modulation of the host response to infection. Cell 1992; 71: 153-167.

20. Vilsmeier B. Paraimmunity-inducing effects of vacinia strain MVA. Berl Munch Tierarztl Wschr 1999; 112: 329-33.

21. Sutter G, Wyatt LS, Foley PL, Bennink JR, Moss B. A recombinant vector derived from the host range-restricted and highly attenuated MVA strain of vaccinia virus stimulates protective immunity in mice to influenza virus. Vaccine 1994; 12: 1032-40.

22. Bender BS, Rowe CA, Taylor SF, Wyatt LS, Moss B, Small, Jr, PA. Oral immunization with a replication-deficient recombinant vaccinia virus protects mice against influenza. J Virol 1996; 70: 6418-6424.

23. Wyatt LS, Shors ST, Murphy BR, Moss B. Development of a replication-deficient recombinant vaccinia virus vaccine effective against parainfluenza virus 3 infection in an animal model. Vaccine 1996; 14: 1451-1458.

24. Carroll MW, Oveerwijk WW, Chamberlain RS, Rosenberg SA, Moss B, Restifo NP. Highly attenuated modified vaccinia virus Ankara (MVA) as an effective recombinant vector: a murine tumor model. Vaccine 1997; 15: 387-94.

25. Schneider J, Gilbert SC, Blanchard TJ, Hanke T, Robson KJ, Hannan CM, Becker M, Sinden R, Smith GL, Hill AVS. Enhanced immunogenicity from CD8+ T cell induction and complete protective efficacy of malaria DNA vaccination by boosting with modified vaccinia virus Ankara. Nature Medicine 1998; 4: 397-402.

26. Durbin AP, Wyatt LS, Siew J, Moss B, Murphy BR. The immunogenicity and efficacy of intranasally or parenterally administered replication-deficient vaccinia-parainfluenza virus type 3 recombinants in rhesus monkeys. Vaccine 1998;16; 1324-30.

27. Nam JH, Wyatt LS, Chae SL, Cho HW, Park YK, Moss B. Protection against lethal Japanese encephalitis virus infection of mice by immunization with the highly attenuated MVA strain of vaccinia virus expressing JEV prM and E genes. Vaccine 1999; 17: 261-8.

28. Huemer HP, Strobl B, Nowotny N. Use of apathogenic vaccinia virus MVA expressing EHV-1 gC as basis of a combined recombinant MVA/DNA vaccination scheme. Vaccine 2000; 18: 1320-6.

29. Hirsch VM, Fuerst TR, Sutter G, Carroll MW, Yang LC, Goldstein S, Piatak Jr M, Elkins WR, Alvord G, Montefiori DC, Moss B, Lifson JD. Patterns of viral replication correlate with outcome in simian immunodeficiency virus (SIV)-infected macaques: effect of prior immunization with a trivalent SIV vaccine in modified vaccinia virus Ankara. J Virol 1996; 70: 3741-52.

30. Hanke T, Blanchard TJ, Schneider J, Hannan CM, Becker M, Gilbert SC, Hill AVS, Smith GL, McMichael A. Enhancement of MHC class I-restricted peptide-specific T cell induction by a DNA prime/MVA boost vaccination regime. Vaccine 1998; 16: 439-445.

31. Seth A, Ourmanov I, Kuroda MJ, Schmitz JE, Carroll MW, Wyatt LS, Moss B, Forman MA, Hirsch VM, Letvin NL. Recombinant modified vaccinia virus Ankara-simian immunodeficiency virus gag pol elicits cytotoxic T lymphocytes in rhesus monkeys detected by a major histocompatibility complex class I/peptide tetramer. PNAS 1998; 95: 10112-6.

32. Hanke T, Samuel RV, Blanchard TJ, Neumann VC, Allen TM, Boyson JE, Sharpe SA, Cook N, Smith GL, Watkins DI, Cranage MP, McMichael AJ. Effective induction of simian immunodeficiency virus-specific cytotoxic T lymphocytes in macaques by using a multiepitope gene and DNA prime-modified vaccinia virus Ankara boost vaccination regimen. J Virol 1999; 73: 7524-32.

33. Hanke T, Blanchard TJ, Schneider J, Ogg GS, Tan R, Becker M, Gilbert SC, Hill AVS, Smith GL, McMichael A. Immunogenicities of intravenous and intramuscular administrations of modified vaccinia virus Ankara-based multi-CTL epitope vaccine for human immunodeficiency virus type 1 in mice. J Gen Virol 1998; 79: 83-90.

34. Belyakov IM, Wyatt LS, Ahlers JD, Earl P, Pendleton CD, Kelsall BL, Strober W, Moss B, Berzofsky JA. Induction of a mucosal cytotoxic T-lymphocyte response by intrarectal immunization with a replication-deficient recombinant vaccinia virus expressing human immunodeficiency virus 89.6 envelope protein. J Virol 1998; 72: 8264-8272.

35. Seth A, Ourmanov I, Schmitz JE, Kuroda MJ, Lifton MA, Nickerson CE, Wyatt L, Carroll M, Moss B, Venzon D, Letvin NL, Hirsch VM. Immunization with a Modified Vaccinia Virus Expressing Simian Immunodeficiency Virus (SIV) Gag-Pol Primes for an Anamnestic Gag-Specific Cytotoxic T-Lymphocyte Response and Is Associated with Reduction of Viremia after SIV Challenge. J Virol 2000; 74: 2502-9.

36. Ourmanov I, Brown CR, Moss B, Carroll M, Wyatt L, Pletneva L, Goldstein S, Venzon D, Hirsch VM. Comparative Efficacy of recombinant Modified Vaccinia Virus Ankara Expressing Simian Immunodeficiency Virus (SIV) Gag-Pol and/or Env in Macaques Challenged with Pathogenic SIV. J Virol 2000; 74: 2740-51.

37. Ourmanov I, Bilska M, Hirsch VM, Montefiori DC. Recombinant Modified Vaccinia Virus Ankara Expressing the Surface gp120 of Simian Immunodeficiency Virus (SIV) Primes for a Rapid Neutralizing Antibody Response to SIV Infection in Macaques. J Virol 2000; 74: 2960-5.

38. Werner GT, Jentzsch U, Metzger E, Simon J. Studies on poxvirus infections in irradiated aniamLs. Archives of Virology 1980; 64: 247-56.

39. Mayr A, Danner K. Vaccination against pox diseases under immunosuppressive conditions. 15th IABS Congress: Vaccinations in the developing countries, La Guadeloupe 1978. Develop Biol Standard 1978; 41: 225-34.

40. Mayr A, Hochstein-Mintzel V, Stickl H. Abstammung, Eigenschaften und Verwendung des attenuierten Vaccinia-Stammes. Infection 1975; 3: 6-14.

41. Mayr A, Stickl H, Muller HK, Danner K, Singer H. Der Pockenimpfstamm MVA: Marker, genetische Struktur, Erfahrungen mit per parenteralen Schutzimpfung und Verhalten im abwehrgeschwachten Organismus. (The smallpox vaccination strain MVA: marker, genetic structure, experience gained with the parenteral vaccination and behavior in organisms with a debilitated defense mechanism). Zentralbl Bakteriol Parasitenkd Infektionskd Hyg Abt I Orig 1978; 167: 375-90.

42. Mayr A, Danner K. Bedeutung von Tierpocken fur den Menschen nach Aufhebung der Pflichtimpfung gegen Pocken. Berl Muench Tieraerztl Wochenschr 1979; 92: 251-6.

43. McCutchan FE, Salminen MO, Carr JK, Burke DS. HIV-1 genetic diversity. AIDS 1996; 10 (Suppl. 3): S13-S20.

44. Kalish ML, Baldwin A, Raktham S, et al. The evolving molecular epidemiology of HIV-1 envelope subtypes in injecting drug users in Bangkok, Thailand: Implications for HIV vaccine trials. AIDS 1995; 9: 851-7.

45. McCutchan FE, Hegerich PA, Brennan TP, Phanuphak P, Singharaj P, Jugsudee A, Berman PWm Gray AM, Fowler AK and Burke DS. Genetic variants of HIV-1 in Thailand. AIDS Res Hum Retrovir 1992; 8:1887-95.

46. McCutchan FE, Artenstein AW, Sanders-Buell E, Salminen MO, Carr JK, Mascola JR, Yu XF, Nelson KE, Khamboonruang C, Schmitt D, Kieny MP, McNeil JG and Burke DS. Diversity of the envelope glycoprotein among human immunodeficiency virus type 1 isolates of clade E from Asia and Africa. J Virol 1996; 70: 3331-8.

47. Carr JK, Salminen MO, Koch C, Gotte D, Artenstein AW, Hegerich PA, St Louis D, Burke DS and McCutchan FE. 1996. Full length sequence and mosaic structure of a human immunodeficiency virus type 1 isolate from Thailand. J Virol 1996; 70: 5935-43.

48. Mascola JR, Louwagie J, McCutchan FE, et al. Two antigenically distinct subtypes of human immunodeficiency virus type 1: Viral genotype predicts neutralization serotype. J Infect Dis 1994;169: 48-54.

49. VanCott TC, Bethke FR, Artenstein AW, et al. Serotyping international HIV-1 isolates by V3 peptides and whole gp160 proteins using BIAcore. Methods: A Companion to Methods Enzymol 1994; 6: 188-98.

50. Ferrari G, Humphrey W, McElrath MJ, et al. Clade B-based HIV-1 vaccines elicit cross-clade cytotoxic T lymphocyte reactivities in uninfected volunteers. Proc Natl Acad Sci USA.1997; 94: 1396-1401.

51. Lynch JA, DeSouza M, Robb MD, Markowitz L, Nitayaphan S, Sapan CV, Mann DL, Birx DL, Cox JH. Cross-clade cytotoxic T cell response to HIV-1 proteins among HLA disparate North Americans and Thais. 1998. J. Infect. Dis. 178: 1040-1046

52. National AIDS Committee. National Plan for Prevention and Alleviation of HIV/AIDS, 1997-2001. Bangkok, 1997 [ISBN 974-7896-44-3].

53. Nitayaphan S, Brown AE. Preventive HIV vaccine development in Thailand. AIDS 12 (suppl B):S155-161, 1998.

54. Thongcharoen P, et al. Future planning for HIV vaccine evaluation of Thai AIDS vaccine evaluation group (TAVEG) in Thailand. 12th World AIDS Conference, Geneva. Abstract 33222.

55. Belshe RB, Gorse GJ, Mulligan MJ, Evans TG, Keefer MC, Excler JL, Duliege AM, Tartaglia J, Cox WI, McNamara J, Hwang KL, Braadney A, Montefiori D, Weinhold KJ for the NIAID AIDS Vaccine Evaluation Group. Induction of immune responses to HIV-1 by canarypox virus (ALVAC) HIV-1 and gp120 SF2 recombinant vaccines in uninfected volunteers. AIDS 1998; 12: 2407-15.

56. Marovich, M et al. "ALVAC-HIV (vCP205) Loaded Autologous DC as an HIV Vaccine Strategy" *Proc XIIIth Cent Gardes Symp* 2003;127-132.

57. Marovich, M et al. "Preparation of Clinical Grade ALVAC-HIV vaccine loaded human dendritic cells" *J Infect Dis* 2002;186: 1242-52.

58. Mwau, M, et al. A human immunodeficiency virus 1 (HIV-1) clade A vaccine in clinical trials: stimulation of HIV-specific T cell responses by DNA and recombinant modified vaccinia virus Ankara (MVA) vaccines in humans. J Gen Virol 2004 85: 911-919.

59. Ramsburg,E. et al. Highly Effective Control of an AIDS Virus Challenge in Macaques by Using Vesicular Stomatitis Virus and Modified Vaccinia Virus Ankara Vaccine Vectors in a Single-Boost Protocol. JV 2004 78:3930-3940.

60. Earl PL Immunogenicity of a highly attenuated MVA smallpox vaccine and protection against monkeypox. Nature. 2004 Mar 11;428(6979):182-5.

61. Moorthy VS, Pinder M, Reece WH, Watkins K, Atabani S, Hannan C, Bojang K, McAdam KP, Schneider J, Gilbert S, Hill AV.Safety and immunogenicity of DNA/modified vaccinia virus ankara malaria vaccination in African adults. J Infect Dis. 2003 Oct 15;188(8):1239-44.

62. Moorthy VS, McConkey S, Roberts M, Gothard P, Arulanantham N, Degano P, Schneider J, Hannan C, Roy M, Gilbert SC, Peto TE, Hill AV. Safety of DNA and modified vaccinia virus Ankara vaccines against liver-stage P. falciparum malaria in non-immune volunteers.Vaccine. 2003 May 16;21(17-18):1995-2002.

63. McConkey SJ, Reece WH, Moorthy VS, Webster D, Dunachie S, Butcher G, Vuola JM, Blanchard TJ, Gothard P, Watkins K, Hannan CM, Everaere S, Brown K, Kester KE, Cummings J, Williams J, Heppner DG, Pathan A, Flanagan K, Arulanantham N, Roberts MT, Roy M, Smith GL, Schneider J, Peto T, Sinden RE, Gilbert SC, Hill AV. Enhanced T-cell immunogenicity of plasmid DNA vaccines boosted by recombinant modified vaccinia virus Ankara in humans. Nat Med. 2003 Jun;9(6):729-35.

64. Gilbert SC, Schneider J, Hannan CM, Hu JT, Plebanski M, Sinden R, Hill AV. Enhanced CD8 T cell immunogenicity and protective efficacy in a mouse malaria model using a recombinant adenoviral vaccine in heterologous prime-boost immunisation regimes. Vaccine. 2002 Jan 15;20(7-8):1039-45.

65. Amara RR, Villinger F, Staprans SI, Altman JD, Montefiori DC, Kozyr NL, Xu Y, Wyatt LS, Earl PL, Herndon JG, McClure HM, Moss B, Robinson HL. Different patterns of immune responses but similar control of a simian-human immunodeficiency virus 89.6P mucosal challenge by modified vaccinia virus Ankara (MVA) and DNA/MVA vaccines. Virol. 2002 Aug;76 (15):7625-31.

66. Vazquez DQ, Blonquist DM, Rodriguez EG, Buch AM, Cano CA. Modified-vaccinia-virus-Ankara (MVA) priming and fowlpox-virus booster elicit a stronger CD8+ T-cell response in mice against an HIV-1 epitope than does a DNA/poxvirus prime-booster approach. Biotechnol Appl Biochem. 2004 Jun; 39(Pt 3):313-8.

67. Gherardi MM, Perez-Jimenez E, Najera JL, Esteban M. Induction of HIV immunity in the genital tract after intranasal delivery of a MVA vector: enhanced immunogenicity after DNA prime-modified vaccinia virus Ankara boost immunization schedule. J Immunol. 2004 May 15;172 (10):6209-20.

68. Wyatt LS, Earl PL, Liu JY, Smith JM, Montefiori DC, Robinson HL, Moss B. Multiprotein HIV type 1 clade B DNA and MVA vaccines: construction, expression, and immunogenicity in rodents of the MVA component. AIDS Res Hum Retroviruses. 2004 Jun;20 (6):645-53.

69. Didierlaurent A, Ramirez JC, Gherardi M, Zimmerli SC, Graf M, Orbea HA, Pantaleo G, Wagner R, Esteban M, Kraehenbuhl JP, Sirard JC. Attenuated poxviruses expressing a synthetic HIV protein stimulate HLA-A2-restricted cytotoxic T-cell responses. Vaccine. 2004 Sep 3;22 (25-26):3395-403.

70. Franchini G, Gurunathan S, Baglyos L, Plotkin S, Tartaglia J. Poxvirus-based vaccine candidates for HIV: two decades of experience with special emphasis on canarypox vectors. Expert Rev Vaccines. 2004 Aug; 3 Suppl 1:S75-88.

71. Im EJ, Hanke T. MVA as a vector for vaccines against HIV-1. Expert Rev Vaccines. 2004 Aug;3 Suppl 1:S89-97.

72. Wyatt LS, Earl PL, Eller LA, Moss B. Highly attenuated smallpox vaccine protects mice with and without immune deficiencies against pathogenic vaccinia virus challenge. Proc Natl Acad Sci U S A. 2004 Mar 30;101(13):4590-5.

73. Stittelaar KJ, Kuiken T, de Swart RL, van Amerongen G, Vos HW, Niesters HG, van Schalkwijk P, van der Kwast T, Wyatt LS, Moss B, Osterhaus AD.Safety of modified vaccinia virus Ankara (MVA) in immune-suppressed macaques. Vaccine. 2001 Jun 14;19(27):3700-9.

74. Full-length sequence and mosaic structure of a human immunodeficiency virus type 1 isolate from Thailand. Carr JK, Salminen MO, Koch C, Gotte D, Artenstein AW, Hegerich PA, St Louis D, Burke DS, McCutchan FE. J Virol. 1996 Sep; 70(9):5935-43.

75. McCutchan FE, Artenstein AW, Sanders-Buell E, Salminen MO, Carr JK, Mascola JR, Yu XF, Nelson KE, Khamboonruang C, Schmitt D, Kieny MP, McNeil JG, Burke DS. Diversity of the envelope glycoprotein among human immunodeficiency virus type 1 isolates of clade E from Asia and Africa. J Virol. 1996 Jun;70(6): 3331-8.

76. McCutchan FE, Hegerich PA, Brennan TP, Phanuphak P, Singharaj P, Jugsudee A, Berman PW, Gray AM, Fowler AK, Burke DS. Genetic variants of HIV-1 in Thailand. AIDS Res Hum Retroviruses. 1992 Nov;8(11):1887-95.
